# Biomimetic Catalytic Remote Desaturation of Aliphatic Alcohols

**DOI:** 10.1021/acs.orglett.4c03623

**Published:** 2024-12-23

**Authors:** Kaiming Zuo, Jing Zhu, Faral Akhtar, Phong Dam, Luis Miguel Azofra, Osama El-Sepelgy

**Affiliations:** † Leibniz Institute for Catalysis e.V., Albert-Einstein-Str. 29a, 18059 Rostock, Germany; ‡ Instituto de Estudios Ambientales y Recursos Naturales (i-UNAT), Universidad de Las Palmas de Gran Canaria (ULPGC), Campus de Tafira, 35017 Las Palmas de Gran Canaria, Spain

## Abstract

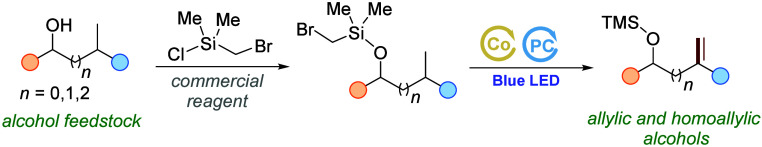

Herein we present photoinduced cobaloxime-catalyzed selective remote desaturation of aliphatic alcohols. This transformation, which proceeds efficiently at room temperature, facilitates the synthesis of valuable cyclic and acyclic allylic and homoallylic alcohols from readily available saturated aliphatic alcohols. Remarkably, this method obviates the need for external oxidants, noble metal catalysts, and phosphine ligands.

Remote desaturation of aliphatic compounds is a valuable transformation in organic chemistry that enables the introduction of unsaturation at positions distant from functional groups or reactive sites within a molecule.^1−3^ This process is particularly challenging due to the inherent stability and inertness of the C–H bonds in aliphatic chains.^4^ We have recently reported cobaloxime-catalyzed remote desaturation of aliphatic amines and amides.^5^ Our next goal involves the further exploration of the catalytic method for the more challenging remote desaturation of aliphatic alcohols.^6,7^

In 2012, Baran introduced an innovative method for remote C–H desaturation of aliphatics using an aryl triazine tether (Scheme 1a).^8^ This approach utilized a tether containing an aryl radical hydrogen abstracting group that, due to its geometric configuration, preferentially facilitates γ-C–H HAT at the tertiary sites.^9^ The reaction involves the formation of a translocated radical^10−12^ followed by radical oxidation using stochiometric oxidant. In 2019, an elegant silicon auxiliary^13,14^ enabled photoexcited Pd-catalyzed remote desaturation of alcohols (Scheme 1b).^15^ The hybrid Pd–radical nature of this protocol enabled the efficient functionalization of different unactivated C–H sites. However, the reaction suffers from the need to use a 10 mol % loading of palladium salt together with expensive phosphine ligand. Importantly, the method also suffers from the necessity to use the highly sensitive and difficult to handle dimethyl(iodomethyl)silane tether for secondary and tertiary alcohols. Inspired by the pioneering work of Gevorgyan,^13,15−17^ Zhang,^18,19^ and Ackermann^20^ and our previous work,^5,21^ we sought on the development of a mild base-metal-catalyzed alcohol desaturation (Scheme 1c).

**Scheme 1:**
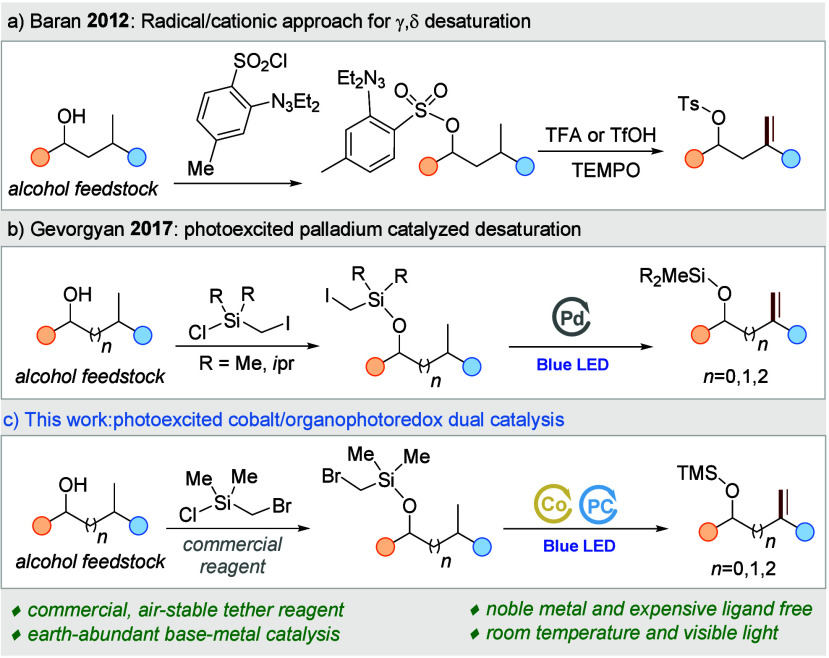
Radical Remote Desaturation of Alcohols

Cobaloxime catalysis,^22^ which mimics the action of vitamin B_12_,^23,24^ offers an intriguing method for the desaturation of aliphatic compounds.^25−27^ This approach leverages its unique ability to convert carbon-centered radicals into the corresponding olefins with high efficiency under mild conditions. These vitamin B_12_ mimics have already demonstrated significant potential in facilitating these challenging desaturation reactions.^21,28−34^

We began our study by using naturally occurring (−)-menthol as a model substrate for desaturation. Initially, we introduced a diisopropyl(iodomethyl)silane tether, commonly employed in Pd-catalyzed transformations. To our delight, using 5 mol % **Co-1** as a single catalyst along with an organic base (condition A) produced the desired product **2a** as a single regioisomer in 70% yield. (Scheme 2) This result suggests a more efficient and sustainable alternative to the Pd/ferrocene-based phosphine catalytic system.^15^ The reaction initiates with the conversion of **Co-1** to the corresponding [Co]^III^–H species under visible-light irradiation, followed by the formation of the supernucleophilic [Co]^I^ species.^21^ The reaction proceeds via an S_N_2 mechanism on the alkyl iodide moiety.

**Scheme 2:**
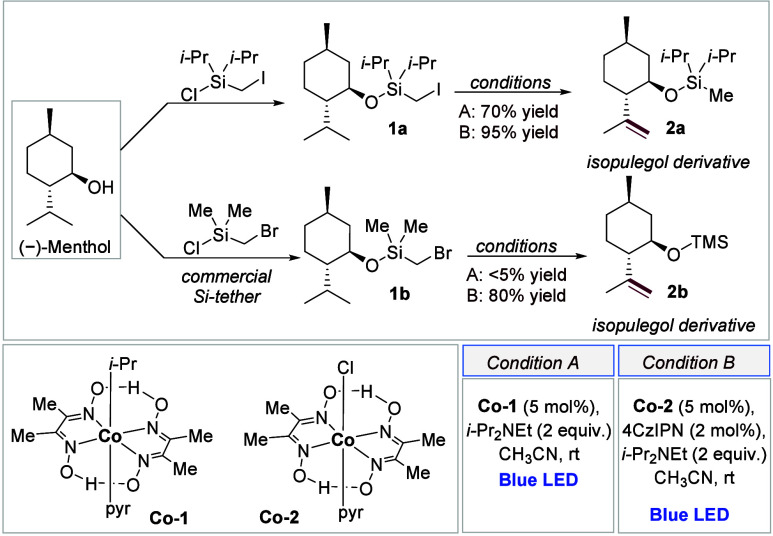
Remote Desaturation of (−)-Menthol

To avoid the multistep synthesis associated with diisopropyl(iodomethyl)silane and handling difficulties of the highly sensitive dimethyl(iodomethyl)silane, we explored using the commercially available and air-stable dimethyl(bromomethyl)silane. However, under condition A, the reaction of **1b** to give **2b** did not proceed due to the lower reactivity of the alkyl bromide **1b** compared to the alkyl iodide substrate **1a** in the S_N_2 pathway. Therefore, we tested the possibility of using an additional organic photocatalyst^35−37^ to generate the silyl methyl radical, which could be trapped by the *in situ*-generated [Co]^II^ species. To our delight, using just a 2 mol % loading of the organic dye 4CzIPN along with **Co-2** (condition B) led to the TMS-protected isoeugenol **2b** as a single regioisomer in 80% yield. Additionally, subjecting the iodo substrate **1a** to reaction condition B afforded the desaturated product **2a** in 95% yield (Scheme 2).

After establishing optimal conditions using naturally occurring menthol, we investigated the generality of the developed catalytic system (Scheme 3). In general, we focused on the use of the commercially available dimethylsilyl tether. However, in some cases we observed slightly higher yields or selectivity when the diisopropylsilyl derivative was used. We began by examining a range of alcohol substrates (**1c**–**1i**) that possess a single tertiary Hγ site and lack of tertiary Hβ or Hδ positions. Various primary and secondary alcohols underwent exclusive γ-/δ-desaturation, yielding their respective homoallylic derivatives in moderate to very good yields (**2c**–**2i**). Notably, the reaction tolerated alcohol derivative **1f**, which contains an additional unsaturation site. Subsequently, to study the competitive selective C–H abstraction and the subsequent cobaloxime-catalyzed desaturation, we tested several challenging substrates with various competitive C–H sites. Desaturation of substrate **1j**, which has both secondary and tertiary Hγ sites, led exclusively to the formation of a tertiary radical and the subsequent γ-/δ-desaturation product **2j** in a good yield. In the case of substrate **1k**, which has different tertiary Hγ sites, a mixture of regioisomers was formed, favoring HAT at the cyclic position. Importantly, competition between 1,5-HAT and 1,6-HAT in alcohol derivatives **1l** and **1m** produced γ-/δ-desaturation products with a very high regioselectivity. Substrates **1n** and **1o**, which have competitive tertiary Hδ sites along with tertiary Hγ sites, reacted exclusively at the γ-C–H sites, yielding **2n** and **2o** in good yields (56–70%). Additionally, tertiary alcohol **1p** was found to be compatible with our desaturation method.

**Scheme 3:**
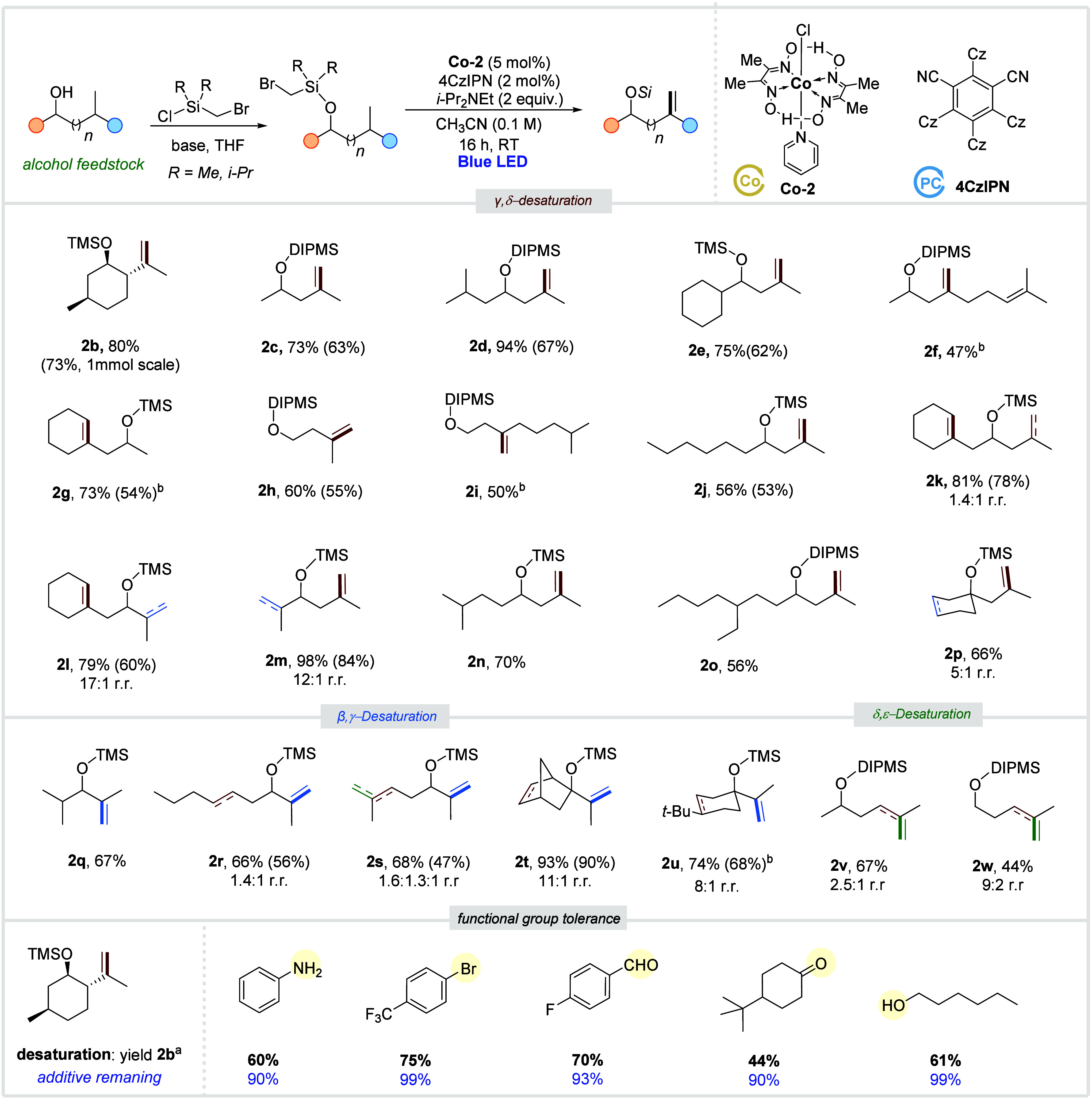
Remote Desaturation of Alcohols **1** (0.2 mmol), **Co-2** (0.01 mmol, 4.0 mg), 4CzIPN (0.004 mmol, 3.2 mg), *i*-Pr_2_NEt (0.2 mmol, 70 μL), CH_3_CN (2 mL), RT, 16 h. NMR yields (1,3,5-trimethoxybenzene as internal standard) are given outside parentheses, and isolated yields are given in parentheses. Contains a minor amount of hydrodehalogenation byproduct. r.r. is the regioisomeric ratio, and DIPMS is diisopropylmethylsilane

After developing a highly regioselective protocol for converting alcohols with Hγ sites to the corresponding homoallylic alcohols, we aimed to extend this method to alcohols lacking Hγ sites. We investigated the desaturation of different secondary and tertiary alcohols bearing isopropyl units to produce the corresponding valuable allylic alcohols **2q**–**2u**. The β-/γ-desaturation process, involving the abstraction of β-sites, occurs via 1,5-HAT. It is worth noting that the hydrogen abstraction from tertiary Hβ site is more favorable than from the secondary Hγ and the tertiary Hδ, as shown in the model examples **2r**–**2u**. Next, we explored the possibility of more challenging distal δ-/ε-desaturation. The secondary and primary alcohols **1v** and **1w** underwent selective δ-/ε-desaturation, yielding desaturated alcohols **2v** and **2w** in moderate yields. Based on the experimental results, our radical desaturation protocol strongly favors the abstraction of tertiary hydrogen atoms at β, γ, and δ over the secondary hydrogen atoms. In addition, the direct competition between the alcohol substrates **1m**, **1n**, and **1s** possessing 3° C–H sites and bearing the same silyl tether enables us to conclude the following preference: 1,6 HAT of Hγ ≫ 1,5 HAT of Hβ > 1,7 HAT.

Finally, an additive robustness study^38^ was conducted on the reaction of **1b** to give **2b** to assess the tolerance of various reactive functional groups. Representative examples demonstrate that the desaturation process uniquely accommodates the presence of aromatic amines, aryl bromides, aldehydes, and ketones with minimal impact on the yield or selectivity (Scheme 3).

To gain deeper insight into the preference for 1,6-HAT regioselectivity, we proceeded with the modeling of saddle points for a series of model substrates that present a varied catalog of HAT events (Figure 1). In this sense, calculations were performed at the PBE0+D3/TZVP//BP91/SVP level of theory in acetonitrile solvent (see the Supporting Information (SI) for details). Cases **a** and **b** show the preference of 1,6-HAT events (red) over 1,5- and 1,7-HAT events (blue), respectively, in tertiary radicals. Since kinetic control determines the preference for the obtained regioisomer in the desaturation process, the transition state (TS) for 1,6- versus 1,5-HAT is favored by 1.0 kcal/mol in case **a**, and the TS for 1,6- versus 1,7-HAT is favored by 2.6 kcal/mol in case **b**, entailing calculated regioisomeric ratios of 84:16 and 99:1, respectively. Case **c** analyzes the competition for HAT between tertiary and secondary radicals, with 1,6-HAT in both cases, showing a preference for 3° Hγ over 2° Hγ by 2.3 kcal/mol, which leads to a theoretically estimated regioisomeric ratio of 98:2. In all cases, calculated Boltzmann populations are in good agreement with the experimental results obtained for these substrates.

**Figure 1:**
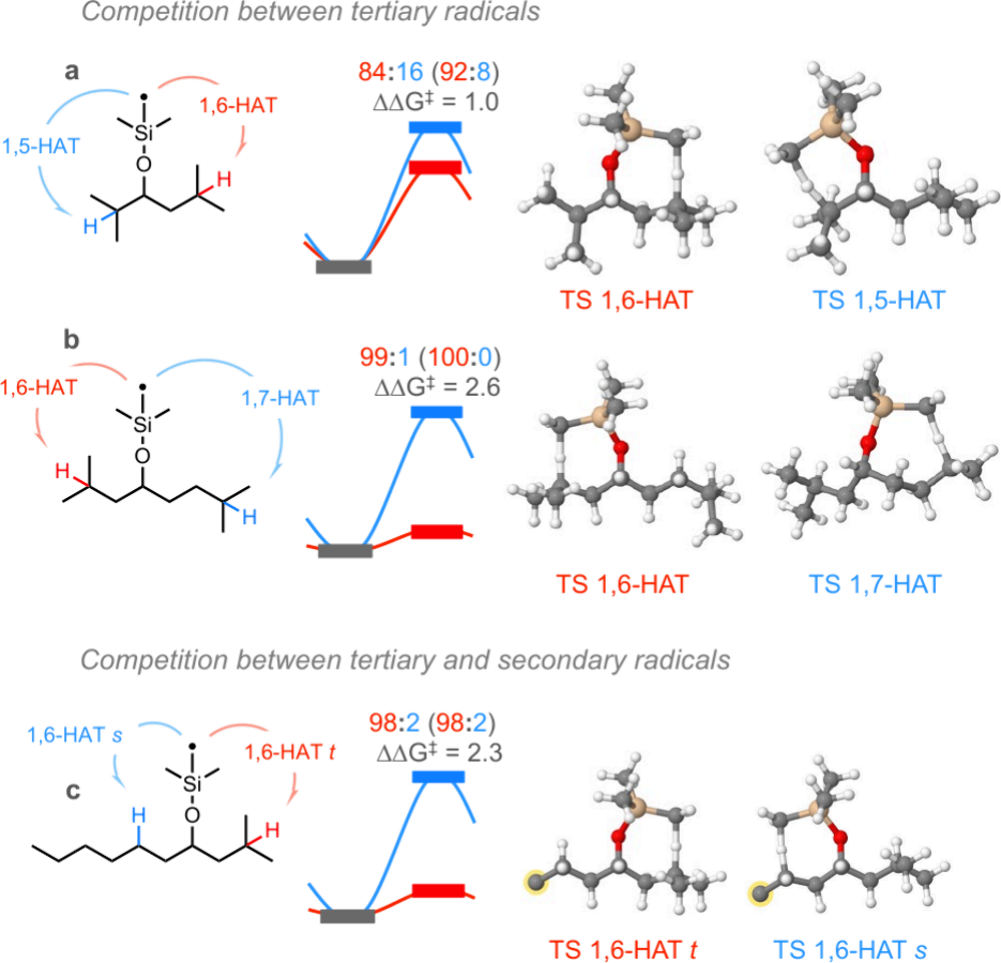
DFT analysis for kinetic control imposing regioselectivity in the desaturation of alcohols. Relative activation free energies, ΔΔ*G*^⧧^, are shown in kcal/mol, and Boltzmann populations, expressed as regioisomeric ratios, were calculated at 25 °C (experimental values are also shown in parentheses for comparison). Optimized structures for TSs are displayed, where carbon atoms highlighted in yellow refer to a CH_2_CH_2_CH_2_CH_3_ moiety, which has been omitted for clarity.

To understand the nature of this stabilization at the thermodynamic level, we observe that in all cases 1,6-HAT presents a lower enthalpy variation than 1,5- and 1,7-HAT (see the SI for details). On the other hand, in cases **b** and **c** we find a more favorable entropy variation for 1,6- versus 1,7-HAT and 3° Hγ versus 2° Hγ, respectively. Although in case **a** a greater disorder is computed for 1,5- versus 1,6-HAT, the TS characterizing 1,5-HAT presents higher angular stress than that for 1,6-HAT. In this sense, the angular stress between 1,6- and 1,7-HAT TSs, whatever the nature of the carbon is (3° or 2°), does not play an important role, as it does in 1,5-HAT.

To gain further insight into the reaction mechanism, a series of UV–vis measurements were performed. Initially, we measured the UV–vis spectrum of the photocatalyst 4CzIPN, which displayed a strong π–π* intraligand transition at 240 nm and a broad band between approximately 320 and 460 nm, representing both localized and delocalized charge transfer regions (Figure 2, black curve).^39^

**Figure 2:**
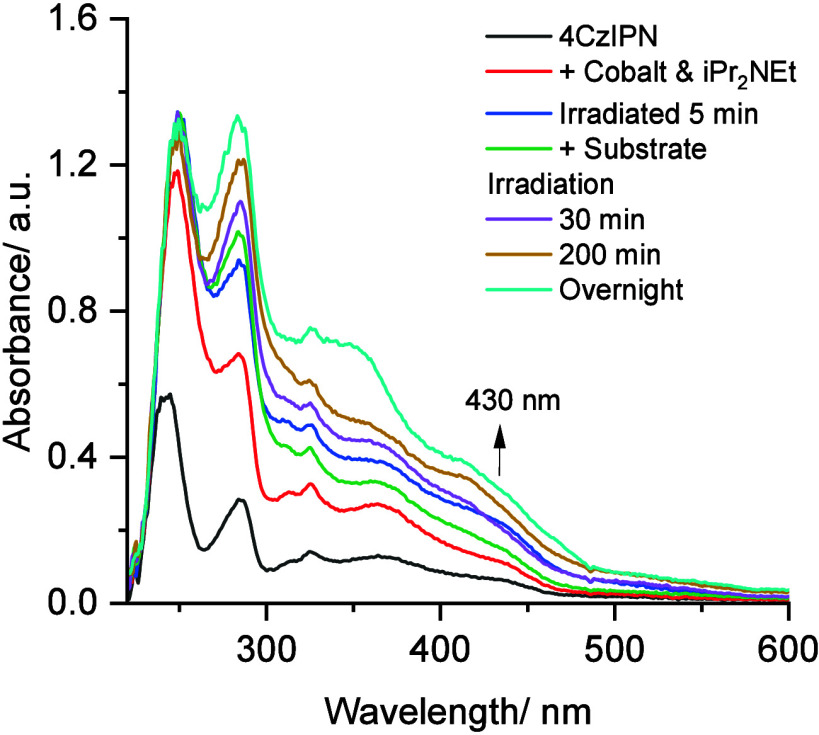
UV–vis spectra of the reaction mixture upon different component additions and irradiation times.

After the addition of Co catalyst and *i*-Pr_2_NEt, the spectrum’s intensity increased due to the appearance of a strong π–π* absorption transition at 250 nm from cobaloxime.^40^ Upon irradiation under blue light, there is a new appearance of a shoulder at 430 nm, which corresponds to the ligand-to-metal charge transfer (LMCT) of [Co]^II^ species (Figure 2, blue curve). The formation of this [Co]^II^ species result from the quenching process of the excited state of 4CzIPN by cobaloxime. The subsequent introduction of a substrate to the mixture led to the disappearance of [Co]^II^ species, indicating the formation of a [Co]^III^–substrate intermediate (Figure 2, green curve). Further irradiation of the complete reaction mixture resulted in stable UV–vis spectra, indicating that the catalyst quickly achieved a steady state, establishing an effective [Co]^III^/[Co]^II^ cycle (Figure 2, purple and yellow curves).

The plausible mechanism is illustrated in Figure 3. The reaction mechanism involves two photocatalytic cycles, two single electron transfer (SET) events, two hydrogen atom transfer (HAT) steps, and a halogen atom transfer (XAT). The reaction starts with the quenching of the excited photocatalyst 4CzIPN* with the cobaloxime catalyst, leading to the formation of the 4CzIPN radical cation and [Co]^II^ species. The XAT of the substrate is enabled by α-amino radical **A**, which is generated in situ from Hünig’s base and the 4CzIPN radical cation.^34^ The carbon-centered radical **C** undergoes internal HAT, leading to the formation of the alkyl radical **D**, and subsequent trapping by the [Co]^II^ results in the formation of the alkylcobalt intermediate **E**. Under visible-light irradiation, the cobalt species **E** undergoes radical-type β-hydrogen elimination, resulting in the formation of the desired unsaturated product and [Co]^III^–H. The reaction between the imine salt **B** and the [Co]^III^–H leads to the regeneration of the [Co]^III^ species and Hünig’s base.

**Figure 3:**
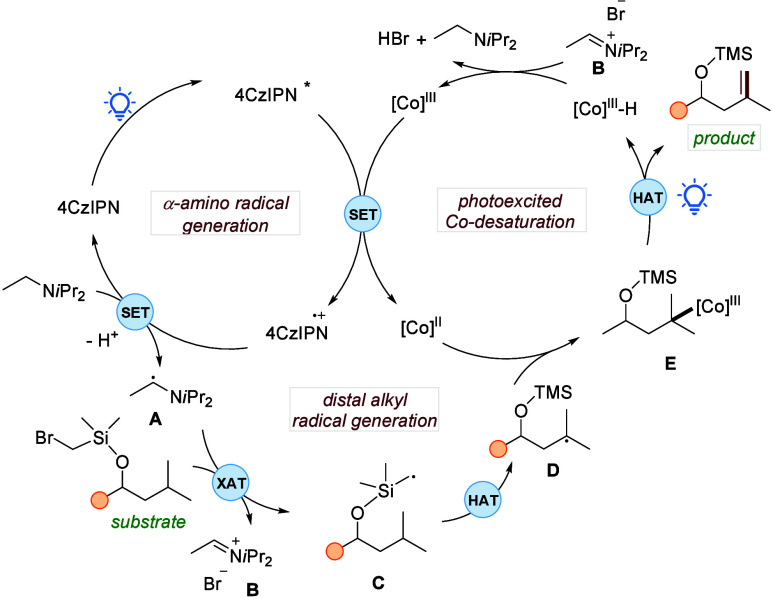
Plausible mechanism.

In summary, we have developed a mild and selective catalytic remote desaturation method for aliphatic alcohols. This approach utilizes two commercially available photocatalysts, 4CzIPN and cobaloxime, eliminating the need for noble metals and complex ligands. DFT calculations indicated a preference for 1,6-HAT over 1,5- and 1,7-HAT in tertiary radicals with energy differences that correlate well with experimental regioisomeric ratios. We believe that this method overcomes limitations of previous approaches, providing a promising new catalytic system for synthesizing valuable allylic and homoallylic alcohols.

## Data Availability

The data underlying this study are available in the published article and its Supporting Information.

## Supporting Information Available

Experimental procedures, analytical data for all new compounds, NMR spectra, and computational information (PDF)

## References

[ref1] Choi J.; MacArthur A. H. R.; Brookhart M.; Goldman A. S. Dehydrogenation and Related Reactions Catalyzed by Iridium Pincer Complexes. Chem. Rev. 2011, 111, 1761–1779. 10.1021/cr1003503. 21391566

[ref2] Giri R.; Maugel N.; Foxman B. M.; Yu J.-Q. Dehydrogenation of Inert Alkyl Groups via Remote C–H Activation: Converting a Propyl Group into a π-Allylic Complex. Organometallics 2008, 27, 1667–1670. 10.1021/om8000444.

[ref3] Baudoin O.; Herrbach A.; Guéritte F. The Palladium-Catalyzed C–H Activation of Benzylic *gem*-Dialkyl Groups. Angew. Chem., Int. Ed. 2003, 42, 5736–5740. 10.1002/anie.200352461. 14661210

[ref4] Zhang X.-M. Homolytic Bond Dissociation Enthalpies of the C–H Bonds Adjacent to Radical Centers. J. Org. Chem. 1998, 63, 1872–1877. 10.1021/jo971768d. 11672334

[ref5] Wang C.; Azofra L. M.; Dam P.; Sebek M.; Steinfeldt N.; Rabeah J.; El-Sepelgy O. Catalytic Desaturation of Aliphatic Amides and Imides Enabled by Excited-State Base-Metal Catalysis. ACS Catal. 2022, 12, 8868–8876. 10.1021/acscatal.2c01723.

[ref6] Čekovió Ž.; Dimttruević L.; Djokić G.; Srnić T. Remote functionalisation by ferrous ion-cupric ion induced decomposition of alkyl hydroperoxides. Tetrahedron 1979, 35, 2021–2026. 10.1016/S0040-4020(01)88972-9.

[ref7] Breslow R.; Baldwin S.; Flechtner T.; Kalicky P.; Liu S.; Washburn W. Remote oxidation of steroids by photolysis of attached benzophenone groups. J. Am. Chem. Soc. 1973, 95, 3251–3262. 10.1021/ja00791a031. 4708826

[ref8] Voica A.-F.; Mendoza A.; Gutekunst W. R.; Fraga J. O.; Baran P. S. Guided desaturation of unactivated aliphatics. Nat. Chem. 2012, 4, 629–635. 10.1038/nchem.1385. 22824894 PMC3405363

[ref9] Hollister K. A.; Conner E. S.; Spell M. L.; Deveaux K.; Maneval L.; Beal M. W.; Ragains J. R. Remote Hydroxylation through Radical Translocation and Polar Crossover. Angew. Chem., Int. Ed. 2015, 54, 7837–7841. 10.1002/anie.201500880. 26014758

[ref10] Nechab M.; Mondal S.; Bertrand M. P. 1,*n*-Hydrogen-Atom Transfer (HAT) Reactions in Which *n* ≠ 5: An Updated Inventory. Chem.—Eur. J. 2014, 20, 16034–16059. 10.1002/chem.201403951. 25345694

[ref11] Sarkar S.; Cheung K. P. S.; Gevorgyan V. C-H functionalization reactions enabled by hydrogen atom transfer to carbon-centered radicals. Chem. Sci. 2020, 11, 12974–12993. 10.1039/D0SC04881J. 34123240 PMC8163321

[ref12] Friese F. W.; Mück-Lichtenfeld C.; Studer A. Remote C–H functionalization using radical translocating arylating groups. Nat. Commun. 2018, 9, 2808 10.1038/s41467-018-05193-6. 30022072 PMC6051993

[ref13] Parasram M.; Gevorgyan V. Silicon-Tethered Strategies for C–H Functionalization Reactions. Acc. Chem. Res. 2017, 50, 2038–2053. 10.1021/acs.accounts.7b00306. 28771325 PMC5724575

[ref14] Wilt J. W.; Lusztyk J.; Peeran M.; Ingold K. U. Absolute rate constants for some intermolecular and intramolecular reactions of α-, β-, and γ-silicon-substituted radicals. J. Am. Chem. Soc. 1988, 110, 281–287. 10.1021/ja00209a045.

[ref15] Parasram M.; Chuentragool P.; Wang Y.; Shi Y.; Gevorgyan V. General, Auxiliary-Enabled Photoinduced Pd-Catalyzed Remote Desaturation of Aliphatic Alcohols. J. Am. Chem. Soc. 2017, 139, 14857–14860. 10.1021/jacs.7b08459. 28992686 PMC5729750

[ref16] Chuentragool P.; Yadagiri D.; Morita T.; Sarkar S.; Parasram M.; Wang Y.; Gevorgyan V. Aliphatic Radical Relay Heck Reaction at Unactivated C(sp^3^)-H Sites of Alcohols. Angew. Chem., Int. Ed. 2019, 58, 1794–1798. 10.1002/anie.201812398. PMC6359925 30462879

[ref17] Kurandina D.; Yadagiri D.; Rivas M.; Kavun A.; Chuentragool P.; Hayama K.; Gevorgyan V. Transition-Metal- and Light-Free Directed Amination of Remote Unactivated C(sp^3^)–H Bonds of Alcohols. J. Am. Chem. Soc. 2019, 141, 8104–8109. 10.1021/jacs.9b04189. 31046256 PMC6873700

[ref18] Cao Z.; Li J.; Sun Y.; Zhang H.; Mo X.; Cao X.; Zhang G. Photo-induced copper-catalyzed alkynylation and amination of remote unactivated C(sp^3^)–H bonds. Chem. Sci. 2021, 12, 4836–4840. 10.1039/D0SC05883A. 34163735 PMC8179574

[ref19] Cao Z.; Li J.; Zhang G. Photo-induced copper-catalyzed sequential 1,*n*-HAT enabling the formation of cyclobutanols. Nat. Commun. 2021, 12, 6404 10.1038/s41467-021-26670-5. 34737326 PMC8569169

[ref20] Wang Y.; Chen S.; Chen X.; Zangarelli A.; Ackermann L. Photo-Induced Ruthenium-Catalyzed Double Remote C(sp^2^)–H/C(sp^3^)–H Functionalizations by Radical Relay. Angew. Chem., Int. Ed. 2022, 61, e202205562 10.1002/anie.202205562. PMC9401009 35527721

[ref21] Wang C.; Azofra L. M.; Dam P.; Espinoza-Suarez E. J.; Do H. T.; Rabeah J.; Brückner A.; El-Sepelgy O. Photoexcited cobalt catalysed endo-selective alkyl Heck reaction. Chem. Commun. 2023, 59, 3862–3865. 10.1039/D2CC06967A. 36883973

[ref22] Dam P.; Zuo K.; Azofra L. M.; El-Sepelgy O. Biomimetic Photoexcited Cobaloxime Catalysis in Organic Synthesis. Angew. Chem., Int. Ed. 2024, 63, e202405775 10.1002/anie.202405775. 38775208

[ref23] Tahara K.; Pan L.; Ono T.; Hisaeda Y. Learning from B12 enzymes: biomimetic and bioinspired catalysts for eco-friendly organic synthesis. Beilstein J. Org. Chem. 2018, 14, 2553–2567. 10.3762/bjoc.14.232. 30410616 PMC6204771

[ref24] Giedyk M.; Goliszewska K.; Gryko D. Vitamin B_12_ catalysed reactions. Chem. Soc. Rev. 2015, 44, 3391–3404. 10.1039/C5CS00165J. 25945462

[ref25] Yu W.-L.; Ren Z.-G.; Ma K.-X.; Yang H.-Q.; Yang J.-J.; Zheng H.; Wu W.; Xu P.-F. Cobalt-catalyzed chemoselective dehydrogenation through radical translocation under visible light. Chem. Sci. 2022, 13, 7947–7954. 10.1039/D2SC02291E. 35865906 PMC9258329

[ref26] Zhou M.-J.; Zhang L.; Liu G.; Xu C.; Huang Z. Site-Selective Acceptorless Dehydrogenation of Aliphatics Enabled by Organophotoredox/Cobalt Dual Catalysis. J. Am. Chem. Soc. 2021, 143, 16470–16485. 10.1021/jacs.1c05479. 34592106

[ref27] West J. G.; Huang D.; Sorensen E. J. Acceptorless dehydrogenation of small molecules through cooperative base metal catalysis. Nat. Commun. 2015, 6, 10093 10.1038/ncomms10093. 26656087 PMC4682047

[ref28] Abrams D. J.; West J. G.; Sorensen E. J. Toward a mild dehydroformylation using base-metal catalysis. Chem. Sci. 2017, 8, 1954–1959. 10.1039/C6SC04607J. 28451310 PMC5384452

[ref29] Cartwright K. C.; Tunge J. A. Decarboxylative Elimination of *N*-Acyl Amino Acids via Photoredox/Cobalt Dual Catalysis. ACS Catal. 2018, 8, 11801–11806. 10.1021/acscatal.8b03282.

[ref30] Huang L.; Ji T.; Zhu C.; Yue H.; Zhumabay N.; Rueping M. Bioinspired desaturation of alcohols enabled by photoredox proton-coupled electron transfer and cobalt dual catalysis. Nat. Commun. 2022, 13, 809 10.1038/s41467-022-28441-2. 35145083 PMC8831637

[ref31] Sun X.; Chen J.; Ritter T. Catalytic dehydrogenative decarboxyolefination of carboxylic acids. Nat. Chem. 2018, 10, 1229–1233. 10.1038/s41557-018-0142-4. 30297751

[ref32] Wang C.; Dam P.; Elghobashy M.; Brückner A.; Rabeah J.; Azofra L. M.; El-Sepelgy O. Biomimetic Dehydroamination of Primary Amines. ACS Catal. 2023, 13, 14205–14212. 10.1021/acscatal.3c04305.

[ref33] Wang X.; Li Y.; Wu X. Photoredox/Cobalt Dual Catalysis Enabled Regiospecific Synthesis of Distally Unsaturated Ketones with Hydrogen Evolution. ACS Catal. 2022, 12, 3710–3718. 10.1021/acscatal.2c00204.

[ref34] Zhao H.; McMillan A. J.; Constantin T.; Mykura R. C.; Juliá F.; Leonori D. Merging Halogen-Atom Transfer (XAT) and Cobalt Catalysis to Override E2-Selectivity in the Elimination of Alkyl Halides: A Mild Route toward contra-Thermodynamic Olefins. J. Am. Chem. Soc. 2021, 143, 14806–14813. 10.1021/jacs.1c06768. 34468137

[ref35] Chan A. Y.; Perry I. B.; Bissonnette N. B.; Buksh B. F.; Edwards G. A.; Frye L. I.; Garry O. L.; Lavagnino M. N.; Li B. X.; Liang Y.; Mao E.; Millet A.; Oakley J. V.; Reed N. L.; Sakai H. A.; Seath C. P.; MacMillan D.W. C. Metallaphotoredox: The Merger of Photoredox and Transition Metal Catalysis. Chem. Rev. 2022, 122, 1485–1542. 10.1021/acs.chemrev.1c00383. 34793128 PMC12232520

[ref36] Ram Bajya K.; Selvakumar S. Dual Photoredox and Cobalt Catalysis Enabled Transformations. Eur. J. Org. Chem. 2022, 2022, e202200229 10.1002/ejoc.202200229.

[ref37] Kojima M.; Matsunaga S. The Merger of Photoredox and Cobalt Catalysis. Trends Chem. 2020, 2, 410–426. 10.1016/j.trechm.2020.01.004.

[ref38] Collins K. D.; Glorius F. A robustness screen for the rapid assessment of chemical reactions. Nat. Chem. 2013, 5 (7), 597–601. 10.1038/nchem.1669. 23787750

[ref39] Brannan A. C.; Beaumont E. F. P.; Phuoc N. L.; Whitehead G. F. S.; Linnolahti M.; Romanov A. S. Organic thermally activated delayed fluorescence material with strained benzoguanidine donor. Beilstein J. Org. Chem. 2023, 19, 1289–1298. 10.3762/bjoc.19.95. 37701304 PMC10494236

[ref40] Kilic A.; Durgun M.; Yorulmaz N.; Yavuz R. The synthesis and investigation of different cobaloximines by spectroscopic methods. J. Mol. Struct. 2018, 1174, 25–31. 10.1016/j.molstruc.2018.03.121.

