# Supplementary material for: Biomimetic Catalytic Remote Desaturation of Aliphatic Alcohols
Source: Org Lett. 2024 Dec 23;27(1):30–5. doi: 10.1021/acs.orglett.4c03623 (PMC11731365; doi:10.1021/acs.orglett.4c03623)
Supplement: Supplementary file 1 — ol4c03623_si_001.pdf [file ol4c03623_si_001.pdf]

Supporting Information

Biomimetic Catalytic Remote Desaturation of Aliphatic alcohols

Kaiming Zuo,[†] Jing Zhu,[†] Faral Akhtar,[†] Phong Dam,[†] Luis Miguel Azofra,[‡] and Osama El-Sepelgy[†]*

[†]Leibniz Institute for Catalysis e.V., Albert-Einstein-Str. 29a, 18059 Rostock, Germany, Germany

[‡]Instituto de Estudios Ambientales y Recursos Naturales (i-UNAT), Universidad de Las Palmas de Gran Canaria (ULPGC), Campus de Tafira, 35017 Las Palmas de Gran Canaria, Spain

Email: Osama.Elsepelgy@Catalysis.de.

Table of Contents

1. General Information 3

2. General procedures 4

3. Characterization data 5

4. Computational details 11

5. Reference 12

7. Optimized XYZ structures 37

1. General Information

All commercial reagents were purchased from commercial suppliers and used without further purification and all solvents were treated according to the general methods. The reactions were monitored by thin layer chromatography (TLC) with aluminum sheets silica gel 60 F_{254} from Merck, and flash column chromatography purifications were performed using silica gel 60 (63-200 μm) from MACHEREY-NAGEL. ^{1}H and ^{13}C NMR spectra were recorded with Bruker AV 300 (300 MHz), AV 400 (400 MHz) or Fourier 300 (300 MHz) NMR spectrometers. Chemical shifts (δ) are given relative to solvent: references for $CDCl_3$ were 7.26 ppm (^{1}H NMR) and 77.16 ppm (^{13}C NMR). And all signals were reported in parts per million (ppm) and spin-spin coupling constants (J) are given in Hz, while multiplicities are abbreviated by s (singlet), d (doublet), t (triplet), q (quartet), br (broad), m (multiplet). All measurements were carried out at room temperature unless otherwise stated.

High-resolution mass spectra (HRMS) were recorded on a sector field mass spectrometer MAT 95-XP (ESI/EI, iron trap). For the products **2d**, **2e**, **2g**, **2n**, **2r** and **2s**, we were able to obtain only low-resolution mass spectrometry (LRMS) data, as high-resolution mass spectrometry (HRMS) was unsuccessful. This limitation arises due to the rapid evaporation of the target molecules under vacuum (starting at approximately 8×10^{-3} mbar in the transfer chamber) and the low stability of the molecular ion, which is sometimes observed at less than 1% in GC-MS data.

For the light-promoted reactions: Use of a blue-LEDs strip (10 W, 21 units, λ max 467, manufacturer: Ledxon). The distance from the light source to the irradiation vessel was about 2 cm, and the reaction vessel was cooled with a fan.

2. General procedures

2.1 General procedure for desaturation of alcohols

In an oven dried 25 mL Schlenk tube equipped with a magnetic stir bar, Si-tethered alcohol (0.2 mmol), 4CzIPN (0.004 mmol 3.2 mg), $Co(dmgH)_2pyCl$[S1] (0.01 mmol, 4 mg) and *i*-Pr_2NEt (0.4 mmol, 70 µL) were added to degassed CH_3CN (2 mL) under the argon atmosphere. Then, the reaction mixture was stirred under blue-LED irradiation at room temperature for 16 h. The residue was then purified by column chromatography on silica gel using hexane as eluent to give pure products.

2.2 Desilylation of the isopulegol derivative 2b

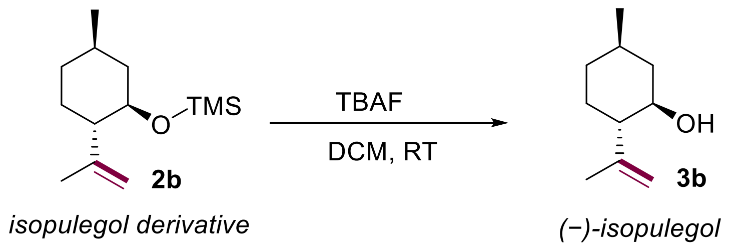

After reaction completion of the desaturation of the substrate **1b**, 10 equiv of TBAF (1 mL, 1M in THF) was added directly to the reaction mixture and stirred for an additional 4h (monitored by TLC). The resulting mixture was diluted with DCM (10 mL), filtered by Celite, and concentrated under reduced pressure. The residue was purified by chromatography column on silica gel using hexane:ethyl acetate (5:1) to produce (-)-isopulegol (**3b**) as pure colorless oil (25.4 mg, 73% yield)

2.3 1 mmol scale desaturation of menthol derivative 1b

In an oven dried 25 mL Schlenk tube equipped with a stir bar, **1b** (1 mmol, 307 mg), 4CzIPN (0.02 mmol 16 mg), $Co(dmgH)_2pyCl$ (0.05 mmol, 20 mg) and *i*-Pr_2NEt (2 mmol, 350 µL) were added to degassed CH_3CN (5 mL) under the argon atmosphere. Then, the reaction mixture was stirred under blue-LED irradiation at room temperature for 16 h. The residue was then purified by column chromatography on silica gel using pentane as eluent to produce **2b** as pure product (164 mg, 73% yield).

3. Characterization data

Trimethyl((5-methyl-2-(prop-1-en-2-yl)cyclohexyl)oxy)silane (2b) [S3]

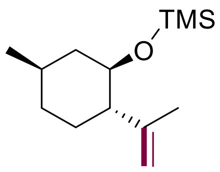

Purification by chromatography (pentane),
Yield: 80% (colorless oil, 33.6 mg)
^{1}H NMR (300 MHz, CDCl$_3$) δ ppm 4.76 – 4.73 (m, 2H), 3.46 (m, 1H), 1.90 – 1.81 (m, 2H), 1.69 (m, 3H), 1.66 – 1.58 (m, 2H), 1.49 – 1.21 (m, 4H), 0.93 – 0.87 (m, 3H), 0.07 (s, 9H).
^{13}C NMR (75 MHz, CDCl$_3$) δ ppm 148.1, 110.9, 73.9, 53.3, 45.3, 34.5, 31.8, 30.6, 22.5, 21.4, -0.6.

diisopropyl(methyl)((4-methylpent-4-en-2-yl)oxy)silane (2c) [S3]

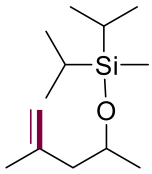

Purification by chromatography (hexane),
Yield: 63% (colorless oil, 27 mg)
^{1}H NMR (300 MHz, CDCl$_3$): δ ppm 4.76 – 4.66 (m, 2H), 4.05 – 3.89 (m, 1H), 2.33 – 2.20 (m, 1H), 2.08 (m, 1H), 1.73 (m, 3H), 1.36 – 1.23 (m, 2H), 1.13 (d, *J* = 6 Hz, 3H), 1.06 – 0.95 (m, 12H), 0.2 (s, 3H).
^{13}C NMR (75 MHz, CDCl$_3$): δ ppm 143.2, 112.8, 67.5, 48.6, 34.3, 23.5, 23.1, 22.5, 17.6, 14.2, 13.5, 13.5, -7.9.

((2,6-dimethylhept-1-en-4-yl)oxy)diisopropyl(methyl)silane (2d)

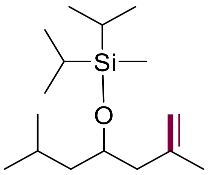

Purification by chromatography (hexane)
Yield: 67% (colorless oil, 49.7 mg)
^{1}H NMR (300 MHz, CDCl$_3$): δ ppm 4.80 – 4.65 (m, 2H), 3.89 (m, 1H), 2.30 – 2.07 (m, 2H), 1.72 (m, 3H), 1.29 (m, 2H), 1.04 – 0.96 (m, 12H), 0.94 – 0.83 (m, 9H), 0.04 (s, 3H).
^{13}C NMR (75 MHz, CDCl$_3$): δ ppm 143.1, 112.9, 69.3, 46.8, 46.4, 24.4, 23.5, 23.2, 22.6, 17.8, 17.8, 13.8, 13.7, -7.3.
LRMS (EI+) calcd. for $C_{16}H_{34}SiO$ [M-H]: 269.23, found: 269.22.

((1-cyclohexyl-3-methylbut-3-en-1-yl)oxy)trimethylsilane (2e)

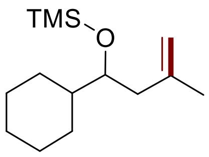

Purification by chromatography (pentane),
Yield: 62% (colorless oil, 30.3 mg)
^{1}H NMR (300 MHz, CDCl$_3$): δ ppm 4.74 (m, 2H), 3.57 (m, 1H), 2.13– 2.04 (m, 1H), 2.17– 2.23 (m, 1H), 1.73 (m, 6H), 1.41 – 0.73 (m, 8H), 0.08 (s, 9H).
^{13}C NMR (75 MHz, CDCl$_3$): δ ppm 143.4, 112.9, 75.5, 43.4, 43.1, 29.9, 27.8, 26.8, 26.7, 26.5, 23.2, 0.7.
LRMS (EI+) calcd. for $C_{14}H_{28}SiO$ [M-H]: 240.19, found: 239.18.

diisopropyl(methyl)((8-methyl-4-methylenenon-7-en-2-yl)oxy)silane (2f) [S3]

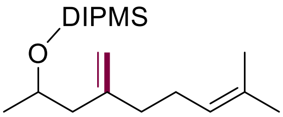

Purification by chromatography (hexane),
Yield: 47% (colorless oil, 33.2 mg)
^{1}H NMR (300 MHz, CDCl$_3$): δ ppm 5.08 (m, 1H), 4.74 (m, 2H), 3.93 (m, 1H), 2.30 – 1.93 (m, 5H), 1.67 (m, 3H), 1.59 (m, 3H), 1.14 – 1.07 (d, J = 6.0 Hz, 3H), 1.03 – 0.91 (m, 15H), 0.03 (s, 3H).
^{13}C NMR (75 MHz, CDCl$_3$) δ ppm 146.9, 131.7, 124.3, 111.7, 67.7, 47.0, 36.5, 26.6, 25.8, 23.6, 17.8, 17.6, 17.6, 13.5, 13.5, -7.9.

((1-(cyclohex-1-en-1-yl)propan-2-yl)oxy)trimethylsilane (2g)

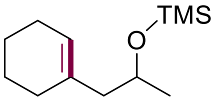

Purification by chromatography (Pentane),
NMR Yield: 54% (colorless oil, 23 mg)
^{1}H NMR (300 MHz, CDCl$_3$): δ ppm 5.42 (m, 1H), 4.00 – 3.78 (m, 1H), 2.17 – 1.85 (m, 6H), 1.72 – 1.49 (m, 4H), 1.11 (m, 3H), 0.10 (s, 9H).
^{13}C NMR (75 MHz, CDCl$_3$): δ ppm 135.2, 123.8, 67.6, 48.6, 29.1, 25.5, 23.8, 23.2, 22.6, 0.4.
LRMS (EI+) calcd. for $C_{12}H_{24}SiO$ [M]: 212.15, found: 212.15.

diisopropyl(methyl)((3-methylbut-3-en-1-yl)oxy)silane (2h) [S3]

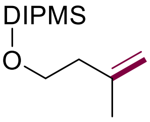

Purification by chromatography (hexane),
Yield: 55% (colorless oil, 47.1 mg)
^{1}H NMR (300 MHz, CDCl$_3$): δ ppm 4.81 – 4.64 (m, 2H), 3.73 (m, 2H), 2.25 (m, 2H), 1.74 (m, 3H), 1.04 – 0.91 (m, 14H), 0.01 (s, 3H).
^{13}C NMR (75 MHz, CDCl$_3$): δ ppm 142.8, 111.2, 62.0, 40.9, 22.6, 17.1, 12.7, -9.0.

diisopropyl(methyl)((7-methyl-3-methyleneoctyl)oxy)silane (2i) [S3]

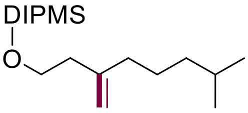

Purification by chromatography (hexane),
NMR yield: 50%.
^{1}H NMR (300 MHz, CDCl$_3$) δ ppm 4.78 – 4.69 (m, 2H), 3.72 (m, 2H), 2.25 (m, 2H), 2.03 – 1.95 (m, 2H), 1.51 (m, 1H), 1.45 – 1.36 (m, 2H), 1.27 – 1.10 (m, 4H), 1.05 – 0.93 (m, 12H), 0.87 (m, 6H), 0.01 (s, 3H).
^{13}C NMR (75 MHz, CDCl$_3$) δ ppm 147.3, 110.4, 62.8, 39.6, 38.9, 37.0, 28.1, 25.7, 22.8, 22.7, 17.6, 17.5, 13.1, -8.5.

trimethyl((2-methyldec-1-en-4-yl)oxy)silane (2j) [S5]

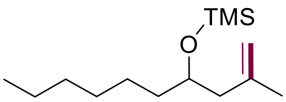

Purification by chromatography (pentane),
Yield: 53% (colorless oil, 23 mg)
^{1}H NMR (300 MHz, CDCl$_3$) δ ppm 4.76 (m, 1H), 4.70 (m, 1H), 3.83 – 3.65 (m, 1H), 2.13 (m, 2H), 1.73 (m, 3H), 1.49 – 1.18 (m, 10H), 0.97 – 0.77 (m, 3H), 0.10 (s, 9H).

^{13}C NMR (75 MHz, CDCl$_3$) δ ppm 143.1, 112.9, 71.4, 46.6, 37.3, 32.0, 29.5, 25.9, 23.2, 22.8, 14.2, 0.6.

((1-(cyclohex-1-en-1-yl)-4-methylpentan-2-yl)oxy)trimethylsilane (2k)

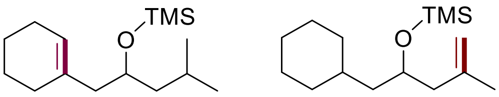

Purification by chromatography (pentane),
Yield: 78% (colorless oil, 40.1 mg)
^{1}H NMR (300 MHz, CDCl$_3$) δ ppm 5.47 – 5.38 (m, 0.46 H), 4.8 – 4.72 (m, 0.38 H), 4.72 – 4.66 (m, 0.37 H), 3.93 – 3.6 (m, 1H), 0.93 – 0.83 (m, 18H), 0.10 (s, 9H).
^{13}C NMR (75 MHz, CDCl$_3$) δ ppm 143.0, 134.9, 124.0, 112.9, 69.6, 68.7, 47.4, 47.1, 46.8, 45.1, 34.4, 34.0, 33.1, 29.1, 26.8, 26.6, 26.4, 25.4, 24.5, 23.7, 23.2, 23.1, 22.6, 22.3, 0.7.
HRMS (ESI) m/z: [M + H]$^+$ Calcd for $C_{15}H_{30}OSi$ 254.2066; Found 254.2060.

((1-(cyclohex-1-en-1-yl)-3-methylbutan-2-yl)oxy)trimethylsilane (2l)

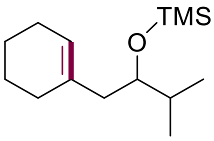

Purification by chromatography (hexane),
Yield: 60% (colorless oil, 35 mg)
^{1}H NMR (300 MHz, CDCl$_3$) δ ppm 5.50 – 5.34 (m, 1H), 3.56 (m, 1H), 2.12 – 1.45 (m, 11H), 0.91 – 0.82 (m, 6H), 0.09 (m, 9H).
^{13}C NMR (75 MHz, CDCl$_3$) δ ppm 135.2, 123.8, 75.8, 43.4, 33.2, 28.9, 25.5, 23.2, 22.6, 19.3, 17.1, 0.7.
HRMS (ESI) m/z: [M + H]$^+$ Calcd for $C_{14}H_{28}OSi$ 240.1909, Found 240.1903.

((2,5-dimethylhex-5-en-3-yl)oxy)trimethylsilane (2m) [S6]

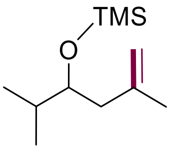

Purification by chromatography (hexane),
Yield: 84% (colorless oil, 41.4 mg)
^{1}H NMR (300 MHz, CDCl$_3$) δ ppm 4.69 – 4.77 (m, 2H), 3.60 (m, 1H), 2.21 – 2.04 (m, 2H), 1.70 – 1.75 (m, 3H), 1.69 – 1.67 (m, 1H), 0.90 – 0.87 (m, 6H), 0.09 (s, 9H).
^{13}C NMR (75 MHz, CDCl$_3$) δ ppm 142.7, 112.2, 75.2, 42.3, 32.5, 22.5, 18.6, 16.5, 0.0.

((2,7-dimethyloct-1-en-4-yl)oxy)trimethylsilane (2n)

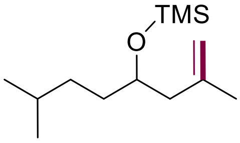

Purification by chromatography (pentane),
NMR yield: 70%
^{1}H NMR (300 MHz, CDCl$_3$) δ ppm 4.77 (m, 1H), 4.70 (m, 1H), 3.81 – 3.67 (m, 1H), 2.15 (m, 2H), 1.73 (m, 3H), 1.53 – 1.06 (m, 5H), 0.88 (m, 6H), 0.10 (s, 9H).
^{13}C NMR (75 MHz, CDCl$_3$) δ ppm 143.1, 112.9, 71.7, 46.5, 35.1(2), 28.2, 23.2, 22.9, 22.7, 0.6.
LRMS (EI+) calcd. for $C_{13}H_{28}SiO$ [M-H]: 227.19, found: 227.18.

((7-ethyl-2-methylundec-1-en-4-yl)oxy)diisopropyl(methyl)silane (2o) [S3]

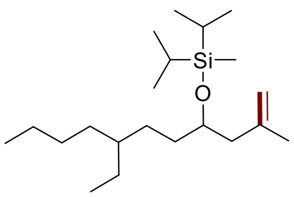

Purification by chromatography (hexane),
Yield: 56% (colorless oil, 38.1 mg)
^{1}H NMR (300 MHz, $CDCl_3$): δ ppm 4.73 (m, 2H), 3.80 (m, 1H), 2.22 – 2.17 (m, 2H), 1.73 (m, 3H), 1.34 – 1.17 (m, 12H), 1.04 – 0.95 (m, 12H), 0.94 – 0.79 (m, 9H), 0.02 (s, 3H).
^{13}C NMR (75 MHz, $CDCl_3$): δ ppm 143.2, 112.8, 71.5, 46.0, 39.2, 39.1, 33.8, 33.7, 33.0, 32.9, 29.1, 28.3, 28.2, 26.2, 26.0, 23.3, 23.2, 17.7, 14.3, 13.7, 13.6, 11.1, 11.0, -7.4.

trimethyl((1-(2-methylallyl)cyclohexyl)oxy)silane (2p) [S7]

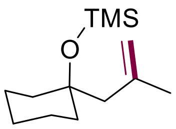

Purification by chromatography (pentane),
Yield: 66% (colorless oil, 29.6 mg)
^{1}H NMR (300 MHz, $CDCl_3$) δ ppm 4.84 (m, 1H), 4.69 (m, 1H), 2.25 (s, 2H), 1.81 (s, 3H), 1.64-1.33 (m, 10H), 0.11 (s, 9H).
^{13}C NMR (75 MHz, $CDCl_3$) δ ppm 143.5, 114.0, 76.3, 48.5, 38.6, 34.6, 25.9, 25.1, 23.4, 3.0.

((2,4-dimethylpent-1-en-3-yl)oxy)trimethylsilane (2q)[S3]

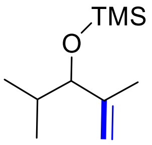

Crude ^{1}H NMR (300MHz, CD_3CN): δ ppm 4.83 (m, 1H), 4.79 (m, 1H), 3.37 (m, 1H), 1.75 – 1.67 (m, 1H), 1.63 (s, 3H), 0.87 – 0.84 (m, 3H), 0.75 (m, 3H), 0.05 (s, 9H).

trimethyl((2-methylnon-1-en-3-yl)oxy)silane (2r)

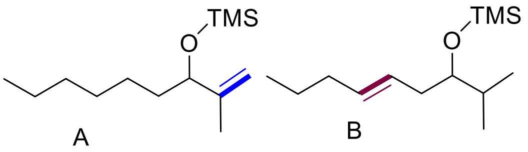

Purification by chromatography (hexane),
Yield: 56% (colorless oil, 25.3 mg)
^{1}H NMR (300 MHz, $CDCl_3$) δ ppm 4.85 (m, 1H), 4.75 (m, 1H), 3.99 (m, 1H), 1.69 – 1.66 (m, 3H), 1.50 – 1.19 (m, 10H), 0.90 – 0.86 (m, 3H), 0.11 – 0.07 (s, 9H).
^{13}C NMR of 2r-A and 2r-B (75 MHz, $CDCl_3$) δ ppm 148.1, 132.5, 127.3, 110.6, 77.8, 77.0, 37.9, 36.3, 35.0, 34.3, 33.0, 32.0, 29.4, 26.0, 22.8, 22.5, 19.1, 17.8, 17.3, 14.3, 0.7, 0.2.
LRMS (EI+) calcd. for $C_{13}H_{28}SiO$ [M]: 228.19, found: 228.19.

((2,6-dimethylhept-1-en-3-yl)oxy)trimethylsilane (2s)

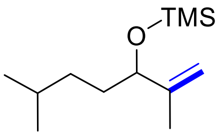

Purification by chromatography (hexane),
Yield: 47% (colorless oil, 41.9 mg)
^{1}H NMR of the major product (300 MHz, $CDCl_3$) δ ppm 4.69 (m, 2H), 3.41 (m, 1H), 2.16 – 1.90 (m, 2H), 1.73 (m, 3H), 1.56 – 1.43 (m, 3H), 0.91 – 0.83 (m, 6H), 0.05 (s, 9H).
LRMS (EI+) calcd. for $C_{12}H_{26}SiO$ [M-H]: 213.17, found: 213.16.

trimethyl(((1S,2S,4R)-2-(prop-1-en-2-yl)bicyclo[2.2.1]heptan-2-yl)oxy)silane (2t) [S3]

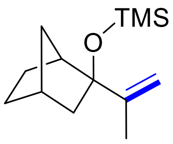

Purification by chromatography (pentane),

Yield: 90% (colorless oil, 40.4 mg)

^{1}H NMR (300 MHz, CDCl$_3$) δ ppm 4.89 – 4.77 (m, 2H), 2.35 (m, 1H), 2.19 – 2.11 (m, 1H), 2.08 – 1.90 (m, 2H), 1.78 (m, 3H), 1.54 – 1.44 (m, 1H), 1.42 – 1.25 (m, 3H), 1.21 – 1.08 (m, 2H), 0.07 (s, 9H).

^{13}C NMR (75 MHz, CDCl$_3$) δ ppm 149.9, 110.2, 84.3, 45.5, 43.2, 37.3, 36.9, 29.6, 22.2, 19.5, 1.9.

HRMS (ESI) m/z: [M $^+$ H]$^+$ Calcd for $C_{13}H_{24}OSi$ 224.1596, Found 224.1590.

(((1s,4s)-4-(tert-butyl)-1-(prop-1-en-2-yl)cyclohexyl)oxy)trimethylsilane (2u) [S3]

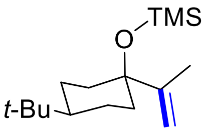

Purification by chromatography (hexane),

Yield: 68% (colorless oil, 36.5 mg)

^{1}H NMR (300 MHz, CDCl$_3$) δ ppm 4.91 (m, 1H), 4.77 (m, 1H), 1.93 – 1.86 (m, 2H); 1.77 (s, 3H), 1.66 –1.26 (m, 7H), 0.86 (m, 9H), 0.09 (s, 9H).

^{13}C NMR (75 MHz, CDCl$_3$) δ ppm 152.1, 109.6, 75.8, 47.8, 37.0, 33.6, 32.3, 27.7, 22.8, 22.3, 19.1, 2.3.

HRMS (ESI) m/z: [M$^+$ H]$^+$ Calcd for $C_{16}H_{32}OSi$ 268.2222, Found 268.2216.

diisopropyl(methyl)((5-methylhex-5-en-2-yl)oxy)silane (2v) [S3]

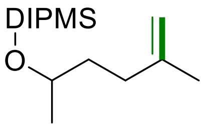

Purification by chromatography (pentane),

Yield: 67% (colorless oil, 32.5 mg)

^{1}H NMR (300 MHz, CDCl$_3$) δ ppm 4.66 (m, 2H), 3.88 – 3.69 (m, 1H), 2.10 – 1.98 (m, 2H), 1.70 (m, 3H), 1.60 – 1.53 (m, 2H), 1.13 (d, J = 6.1 Hz, 3H), 0.97 (m, 14H), 0.00 (s, 3H).

^{13}C NMR (75 MHz, CDCl$_3$) δ ppm 146.1, 109.5, 68.4, 37.8, 33.8, 25.8, 23.6, 22.6, 17.4, -7.9.

diisopropyl(methyl)((4-methylpent-4-en-1-yl)oxy)silane (2w) [S3]

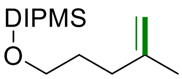

Purification by chromatography (pentane),

Yield: 44% (colorless oil, 20 mg)

^{1}H NMR (300 MHz, CDCl$_3$) δ ppm 4.78 – 4.61 (m, 2H), 3.62 (m, 2H), 2.12 – 1.98 (m, 2H), 1.74 – 1.70 (s, 3H), 1.69 (m, 2H), 0.98 (m, 14H), -0.00 (s, 3H).

^{13}C NMR (75 MHz, CDCl$_3$) δ ppm 145.7, 109.8, 63.0, 34.0, 30.9, 22.5, 17.4, 17.4, 17.4, 13.0, -8.7.

5-methyl-2-(prop-1-en-2-yl)cyclohexan-1-ol (3b) [S4]

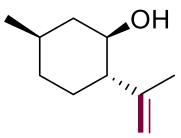

^{1}H NMR (300 MHz, $CDCl_3$) δ ppm 4.94 – 4.82 (m, 2H), 3.53 – 3.34 (m, 1H), 2.09 – 2.00 (m, 1H), 1.98 – 1.78 (m, 2H), 1.72 – 1.70 (m, 3H), 1.55 – 1.23 (m, 5H), 0.96 – 0.93 (m, 3H).

^{13}C NMR (75 MHz, $CDCl_3$): δ ppm 146.7, 112.9, 70.4, 54.2, 42.7, 34.3, 31.5, 29.7, 22.2, 19.2.

4. Computational details

Initial guesses were optimized using the generalized gradient approximation (GGA) Becke–Perdew exchange-correlation functional BP91[S8-S10]and the split-valence double-ξ SVP basis set,[S11] implicitly simulating the solvent effects through the conductor-like screening solvation model (COSMO). In this regard, acetonitrile (ε = 35.688) was employed as solvent. Over these optimized structures, harmonic frequency calculations were performed to confirm the nature of the stationary points, *i.e.*, minima or first-order transition states (TS) with none and one imaginary frequency, in each case. Secondly, single-point energy refinement calculations were carried out at the PBE0[5]/TZVP level of theory in COSMO[S12, S13] that is, making use of the more complete split-valence triple- ξ TZVP basis set.[S14] In all cases, the total energy target accuracy for numeral integration has been set in 10^{-7}, corresponding to a fine grid. All calculations were carried out through the facilities provided by the NWChem package (version 6.8.1).[S15, S16]

Table S1. Free energies (kcal/mol), and comparison of relative enthalpies (kcal/mol) and entropies (cal/mol·K) for the TSs in the models offered at **Figure 1** in the main text. Note: DZ and TZ refer to double- and triple-ξ basis sets, respectively.

**Case a**	Δ*G*(TZ)	ΔΔ*H*(DZ)	ΔΔ*S*(DZ)
Reactant	0.0		
TS 1,5-HAT	10.3	0.2	0.29
TS 1,6-HAT	9.3	0.0	0.00
**Case b**	Δ*G*(TZ)	ΔΔ*H*(DZ)	ΔΔ*S*(DZ)
Reactant	0.0		
TS 1,7-HAT	12.0	3.0	–1.21
TS 1,6-HAT	9.4	0.0	0.00
**Case c**	Δ*G*(TZ)	ΔΔ*H*(DZ)	ΔΔ*S*(DZ)
Reactant	0.0		
TS 1,6-HAT (2º)	11.5	2.2	–0.94
TS 1,6-HAT (3º)	9.3	0.0	0.00

5. Reference.

[S1] Liu, W.-Q.; Lei, T.; Zhou, S.; Yang, X.-L.; Li, J.; Chen, B.; Sivaguru, J.; Tung, C.-H.; Wu, L.-Z. Cobaloxime Catalysis: Selective Synthesis of Alkenylphosphine Oxides under Visible Light. *J. Am. Chem. Soc.* **2019**, *141*, 13941-13947.

[S2] Chlor(pyridin)bis(dimethylglyoximat)cobalt(III) | Sigma-Aldrich (sigmaaldrich.com)

[S3] Parasram, M; Chuentragool, P; Wang, Y; Shi Y; Gevorgyan, V.; General, Auxiliary-Enabled Photoinduced Pd-Catalyzed Remote Desaturation of Aliphatic Alcohols. *J Am Chem Soc*. **2017**,139, 14857-14860.

[S4] Kikukawa, Y.; Yamaguchi, S.; Nakagawa, Y.; Uehara, K.; Uchida, S.; Yamaguchi, K.; Mizuno, N. Synthesis of a Dialuminum-Substituted Silicotungstate and the Diastereoselective Cyclization of Citronellal Derivatives. *Journal of the American Chemical Society* **2008**, *130* (47), 15872-15878.

[S5] Hargaden, G. C.; McManus, H. A.; Cozzi, P. G.; Guiry, P. J. The application of bis(oxazoline) ligands in the catalytic enantioselective methallylation of aldehydes. *Org Biomol Chem* **2007**, *5* (5), 763-766.

[S6] Ratnikov, M. O.; Tumanov, V. V.; Smit, W. A. Lewis acid catalyst free electrophilic alkylation of silicon-capped pi donors in 1,1,1,3,3,3-hexafluoro-2-propanol. *Angew Chem Int Ed Engl* **2008**, *47* (50), 9739-9742.

[S7] Liu, J.; Tong, X.; Chen, M. Allylboration of Ketones and Imines with a Highly Reactive Bifunctional Allyl Pinacolatoboronate Reagent. *J Org Chem* **2020**, *85* (8), 5193-5202.

[S8] Becke, A. D. Density-Functional Exchange-Energy Approximation with Correct Asymptotic Behavior. *Phys. Rev. A* **1988**, *38*, 3098–3100.

[S9] Perdew, J. P.; Chevary, J. A.; Vosko, S. H.; Jackson, K. A.; Pederson, M. R.; Singh, D. J.; Fiolhais, C. Atoms, Molecules, Solids, and Surfaces: Applications of the Generalized Gradient Approximation for Exchange and Correlation. *Phys. Rev. B* **1992**, *46*, 6671–6687.

[S10] Perdew, J. P.; Chevary, J. A.; Vosko, S. H.; Jackson, K. A.; Pederson, M. R.; Singh, D. J.; Fiolhais, C. Erratum: Atoms, Molecules, Solids, and Surfaces: Applications of the Generalized Gradient Approximation for Exchange and Correlation. *Phys. Rev. B* **1993**, *48*, 4978.

[S11] Weigend, F.; Ahlrichs, R. Balanced Basis Sets of Split Valence, Triple Zeta Valence and Quadruple Zeta Valence Quality for H to Rn: Design and Assessment of Accuracy. *Phys. Chem. Chem. Phys.* **2005**, 3297–3305.

[S12] Adamo, C.; Barone, V. Toward Reliable Density Functional Methods without Adjustable Parameters: The PBE0 Model. *J. Chem. Phys.* **1999**, *110*, 6158–6170.

[S13] Klamt, A.; Schüürmann, G. COSMO: A New Approach to Dielectric Screening in Solvents with Explicit Expressions for the Screening Energy and Its Gradient. *J. Chem. Soc., PerkTransactions 2* **1993**, 799–805.

[S14] York, D. M.; Karplus, M. A Smooth Solvation Potential Based on the Conductor-Like Screening Model. *J Phys Chem A* **1999**, *103*, 11060–11079.

[S15] Valiev, M.; Bylaska, E. J.; Govind, N.; Kowalski, K.; Straatsma, T. P.; Van Dam, H. J. J.;

Wang, D.; Nieplocha, J.; Apra, E.; Windus, T. L.; de Jong, W. A. NWChem: A Comprehensive and Scalable Open-Source Solution for Large Scale Molecular Simulations. *Comput Phys Commun* **2010**, *181* (9), 1477–1489.

[S16] Aprà, E.; Bylaska, E. J.; de Jong, W. A.; Govind, N.; Kowalski, K.; Straatsma, T. P.; Valiev, M.; van Dam, H. J. J.; Alexeev, Y.; Anchell, J.; Anisimov, V.; Aquino, F. W.; Atta-Fynn, R.; Autschbach, J.; Bauman, N. P.; Becca, J. C.; Bernholdt, D. E.; Bhaskaran-Nair, K.; Bogatko, S.; Borowski, P.; Boschen, J.; Brabec, J.; Bruner, A.; Cauët, E.; Chen, Y.; Chuev, G. N.; Cramer, C. J.; Daily, J.; Deegan, M. J. O.; Dunning, T. H.; Dupuis, M.; Dyall, K. G.; Fann, G. I.; Fischer, S. A.; Fonari, A.; Früchtl, H.; Gagliardi, L.; Garza, J.; Gawande, N.; Ghosh, S.; Glaesemann, K.; Götz, A. W.; Hammond, J.; Helms, V.; Hermes, E. D.; Hirao, K.; Hirata, S.; Jacquelin, M.; Jensen, L.; Johnson, B. G.; Jónsson, H.; Kendall, R. A.; Klemm, M.; Kobayashi, R.; Konkov, V.; Krishnamoorthy, S.; Krishnan, M.; Lin, Z.; Lins, R. D.; Littlefield, R. J.; Logsdail, A. J.; Lopata, K.; Ma, W.; Marenich, A. V; del Campo, J.; Mejia-Rodriguez, D.; Moore, J. E.; Mullin, J. M.; Nakajima, T.; Nascimento, D. R.; Nichols, J. A.; Nichols, P. J.; Nieplocha, J.; Otero-de-la-Roza, A.; Palmer, B.; Panyala, A.; Pirojsirikul, T.; Peng, B.; Peverati, R.; Pittner, J.; Pollack, L.; Richard, R. M.; Sadayappan, P.; Schatz, G. C.; Shelton, W. A.; Silverstein, D. W.; Smith, D. M. A.; Soares, T. A.; Song, D.; Swart, M.; Taylor, H. L.; Thomas, G. S.; Tipparaju, V.; Truhlar, D. G.; Tsemekhman, K.; Van Voorhis, T.; Vázquez-Mayagoitia, Á.; Verma, P.; Villa, O.; Vishnu, A.; Vogiatzis, K. D.; Wang, D.; Weare, J. H.; Williamson, M. J.; Windus, T. L.; Woliński, K.; Wong, A. T.; Wu, Q.; Yang, C.; Yu, Q.; Zacharias, M.; Zhang, Z.; Zhao, Y.; Harrison, R. J. NWChem: Past, Present, and Future. *J. Chem. Phys.* **2020**, *152*, 184102.

6. NMR Spectra.

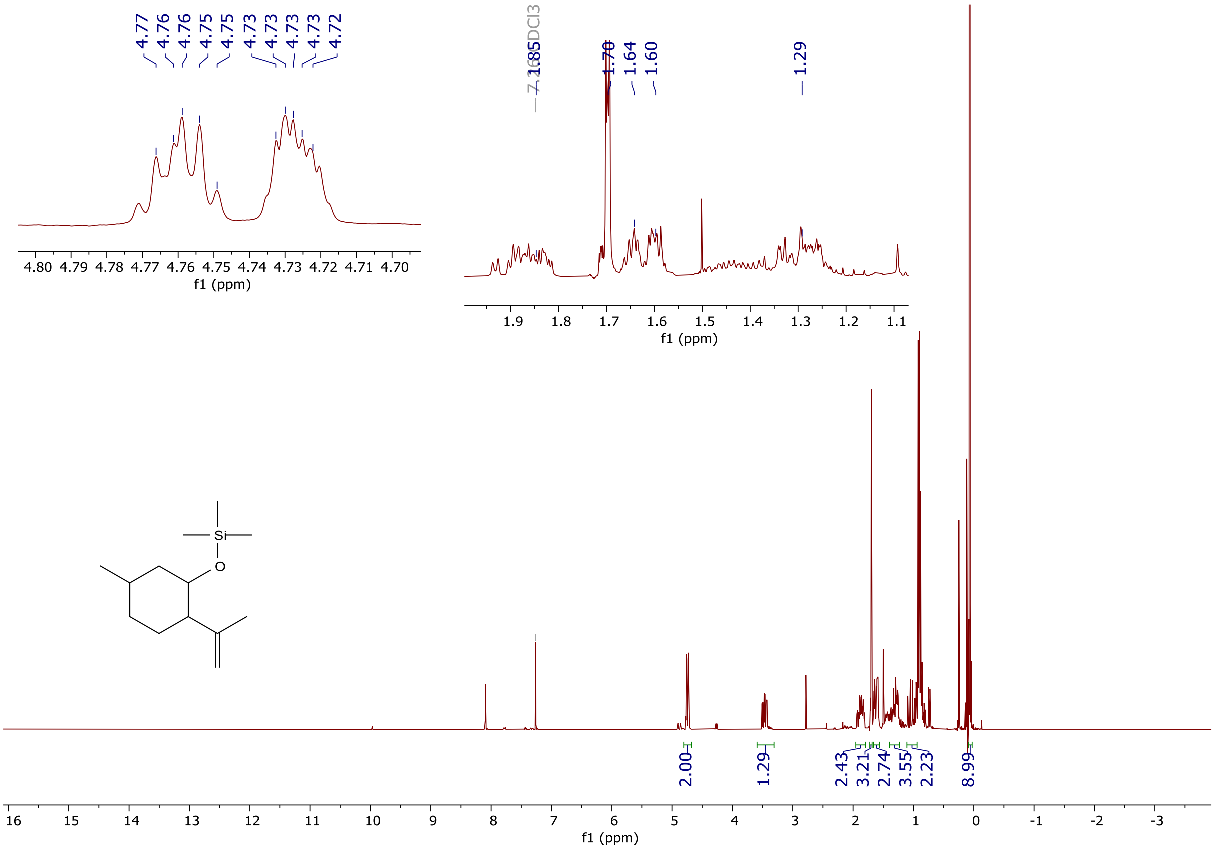

^{1}H NMR Spectrum of **2b** (300MHz, $CDCl_3$)

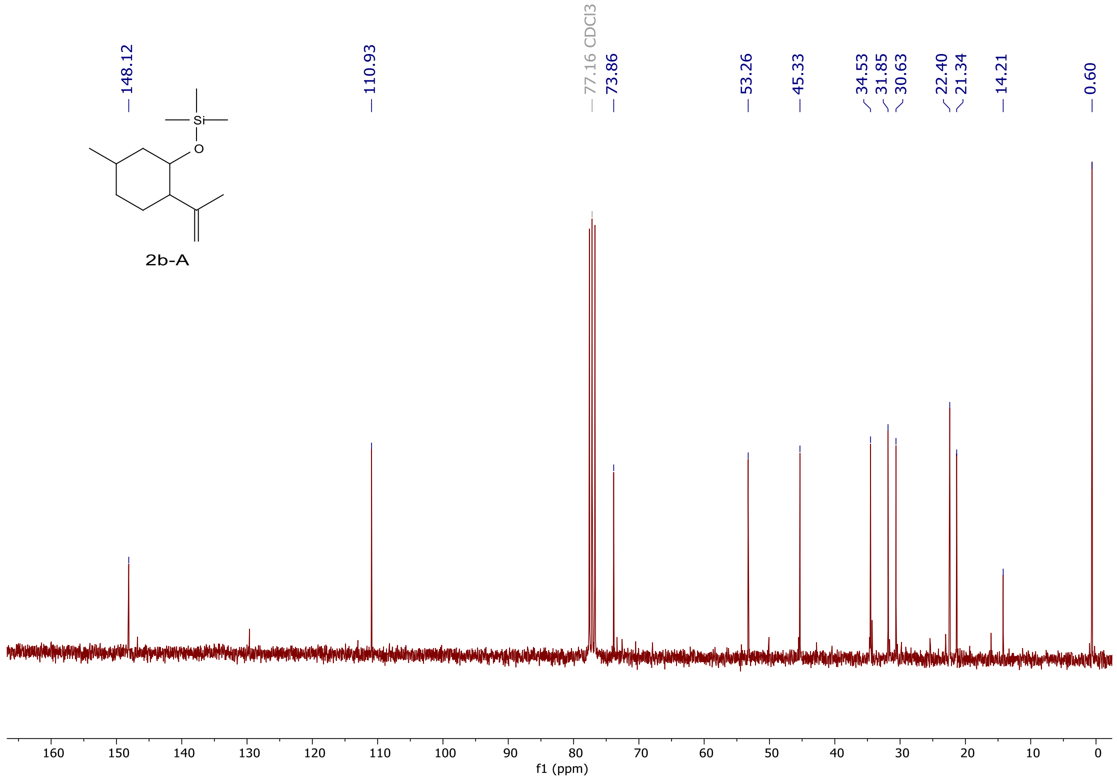

^{13}C NMR Spectrum of **2b** (101MHz, $CDCl_3$)

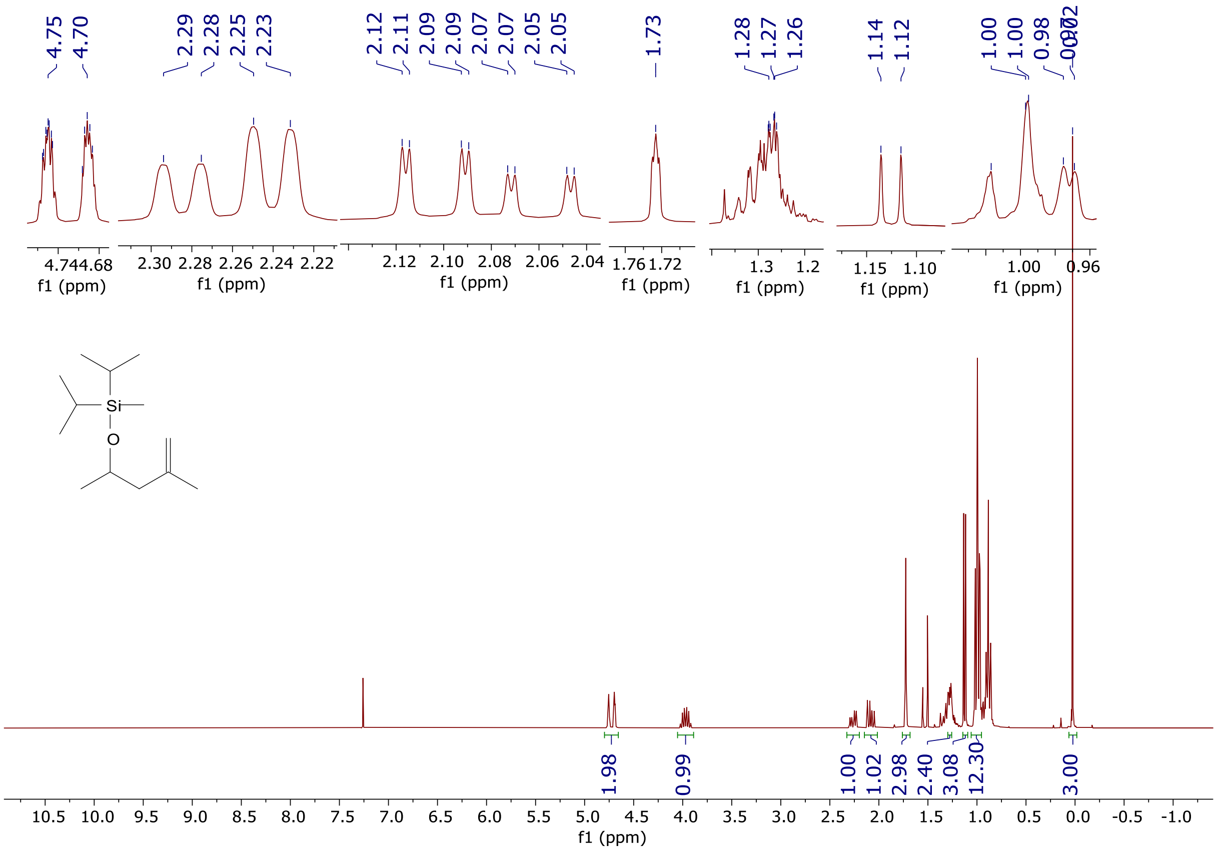

^{1}H NMR Spectrum of **2c** (300MHz, $CDCl_3$)

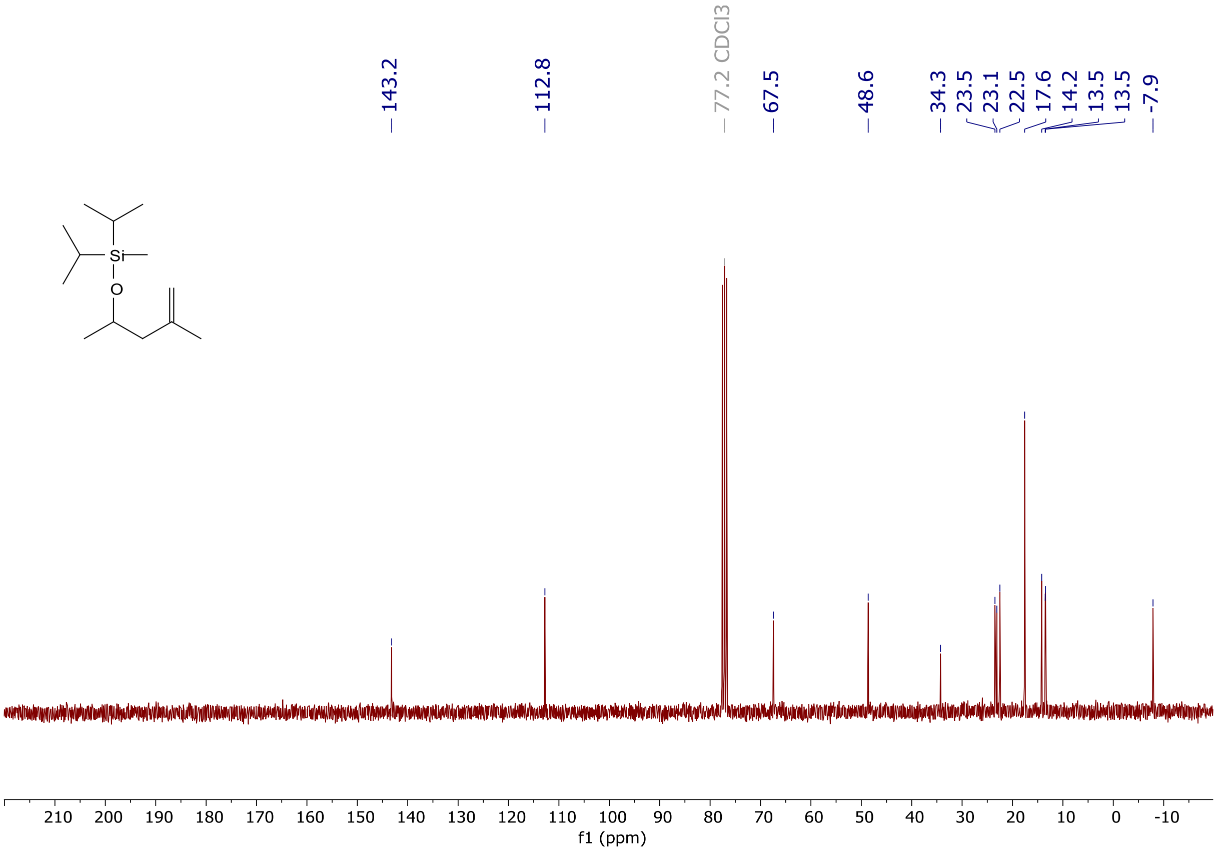

^{13}C NMR Spectrum of **2c (**101MHz, $CDCl_3$)

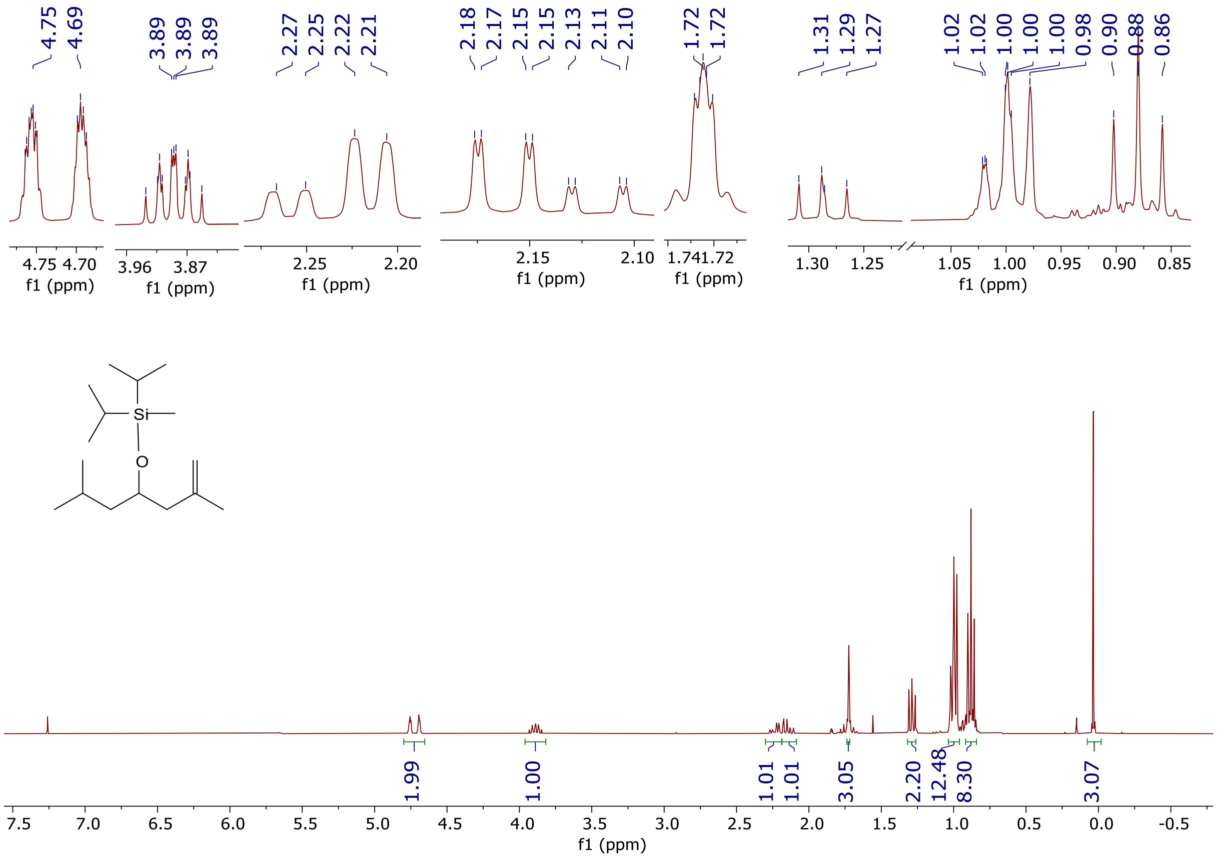

^{1}H NMR Spectrum of **2d** (300MHz, $CDCl_3$)

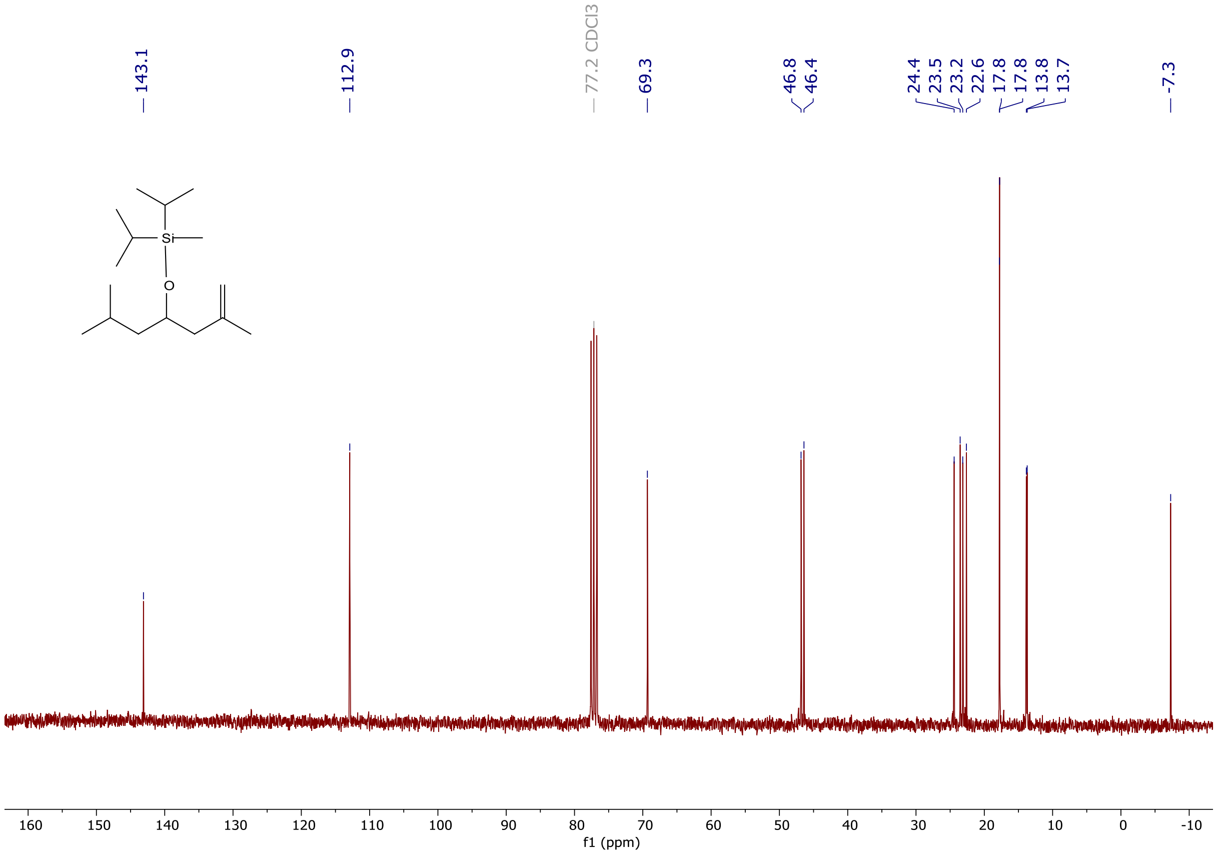

^{13}C NMR Spectrum of **2d (**101MHz, $CDCl_3$)

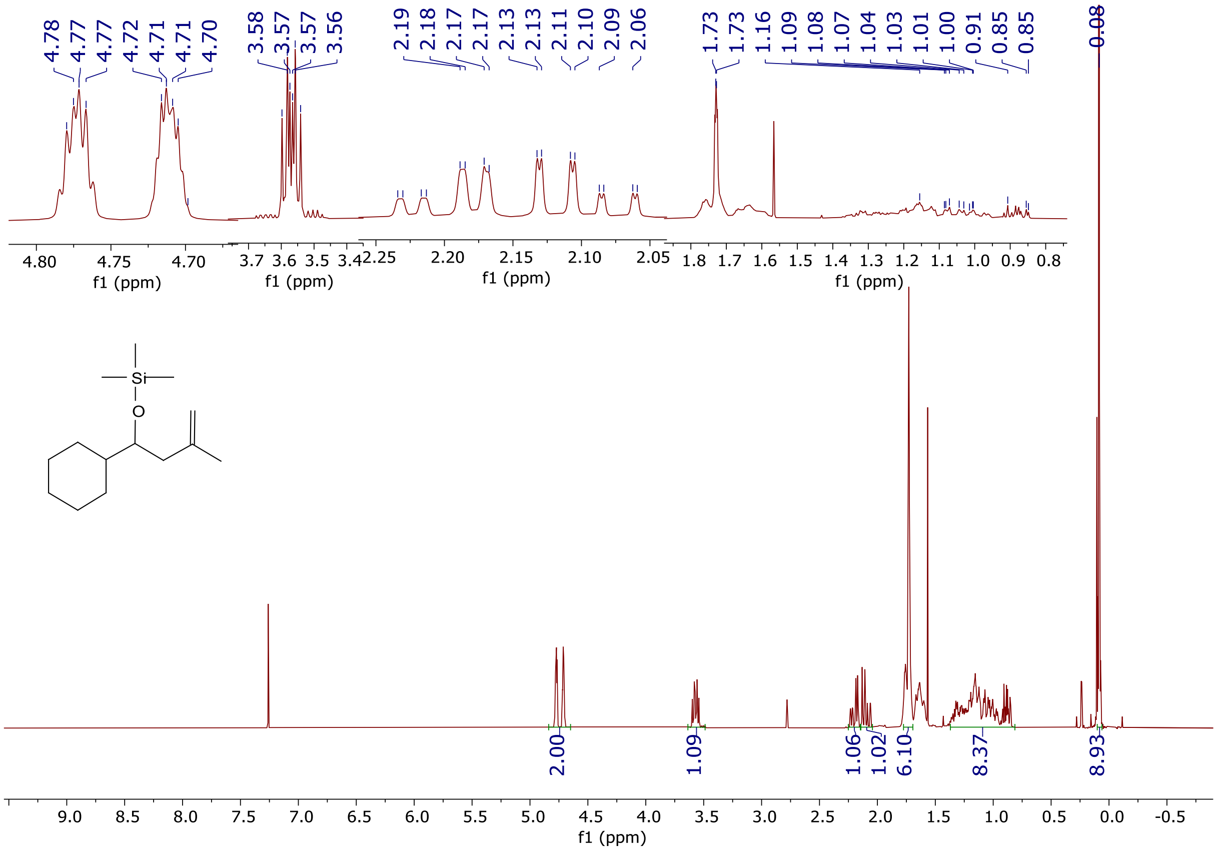

^{1}H NMR Spectrum of **2e** (300MHz, $CDCl_3$)

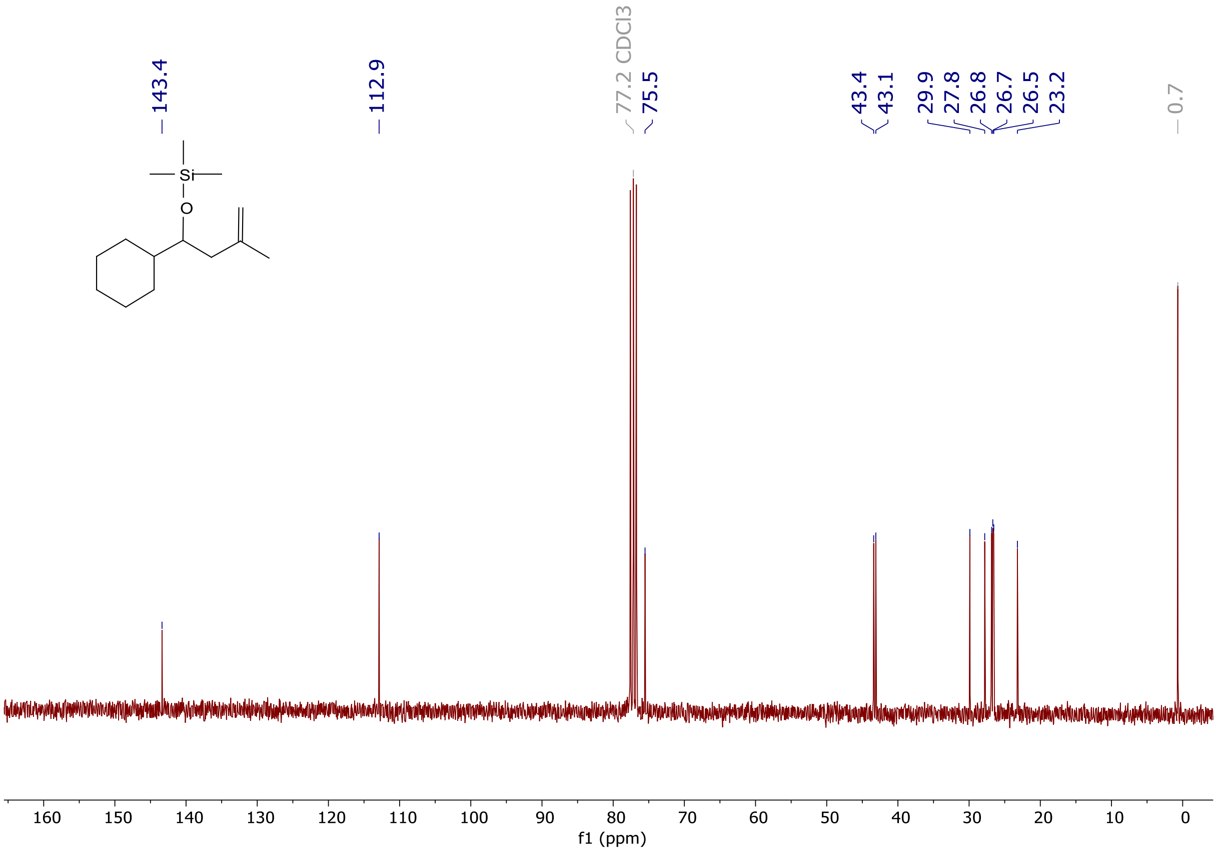

^{13}C NMR Spectrum of **2e** (101MHz, $CDCl_3$)

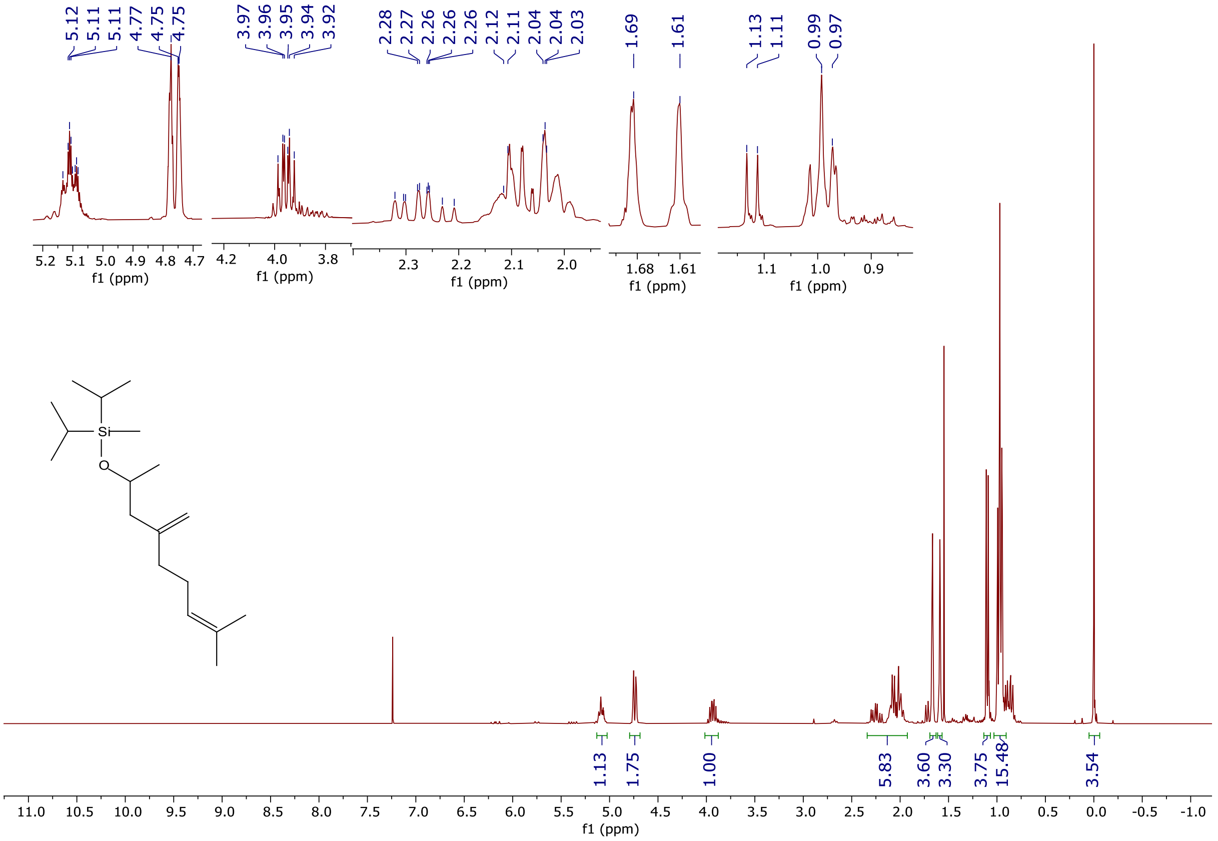

^{1}H NMR Spectrum of **2f** (300MHz, $CDCl_3$, Contains minor amount of hydrodehalogenation byproduct.)

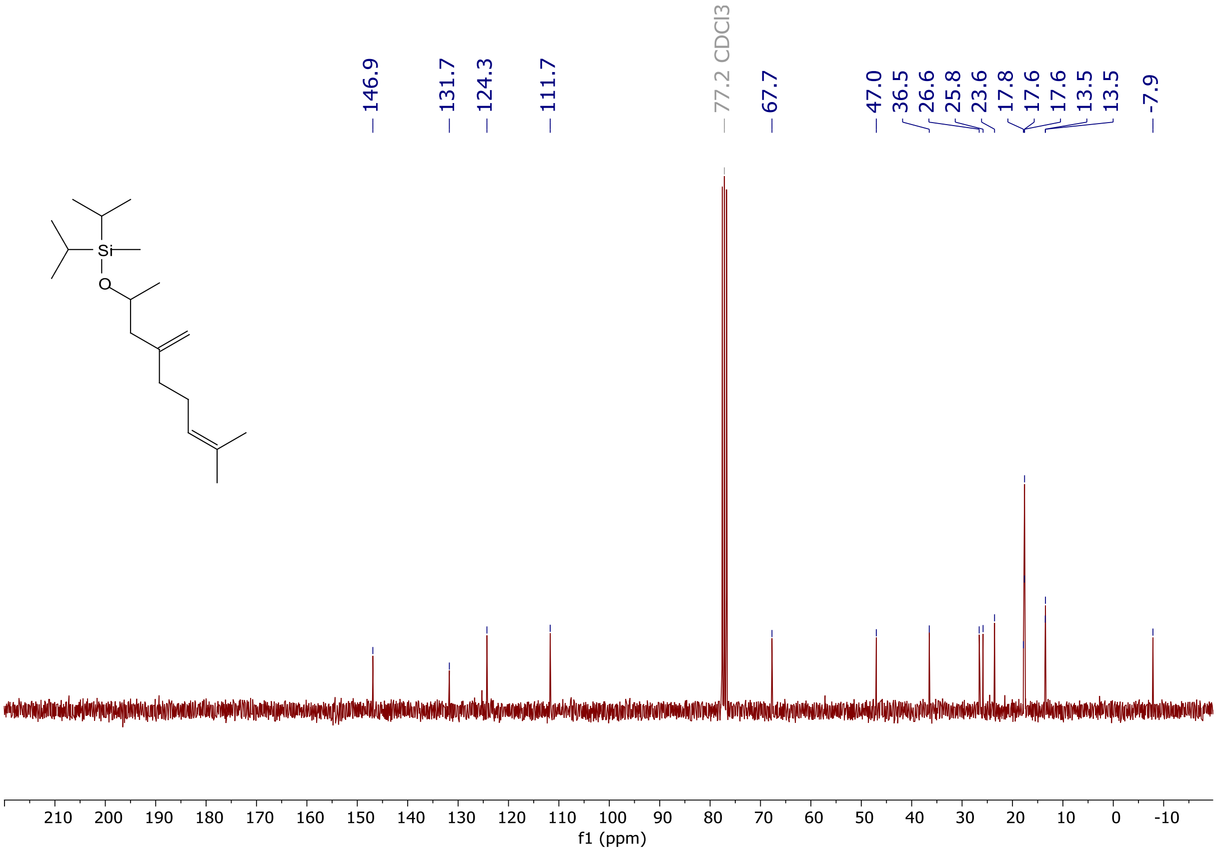

^{13}C NMR Spectrum of **2f** (101MHz, $CDCl_3$)

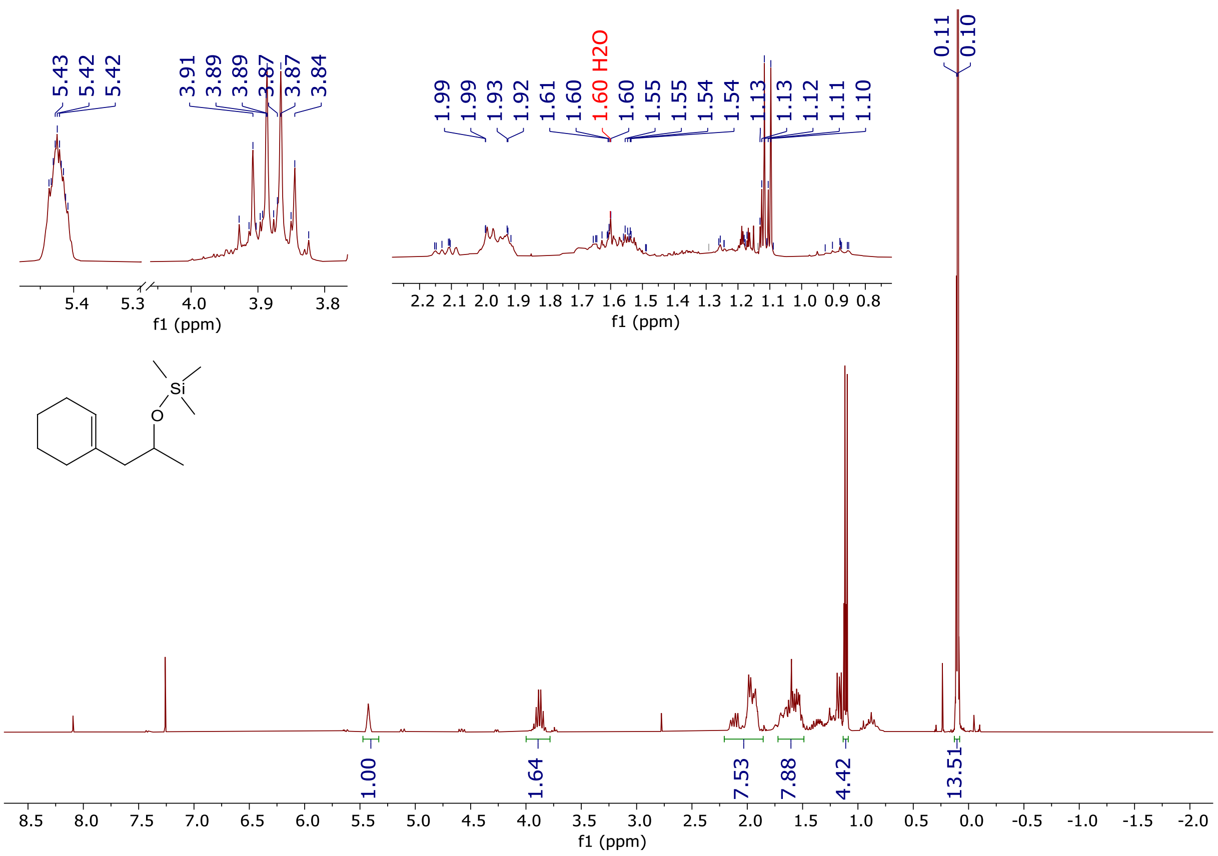

^{1}H NMR Spectrum of **2g** (300MHz, $CDCl_3$, Contains minor amount of hydrodehalogenation byproduct.)

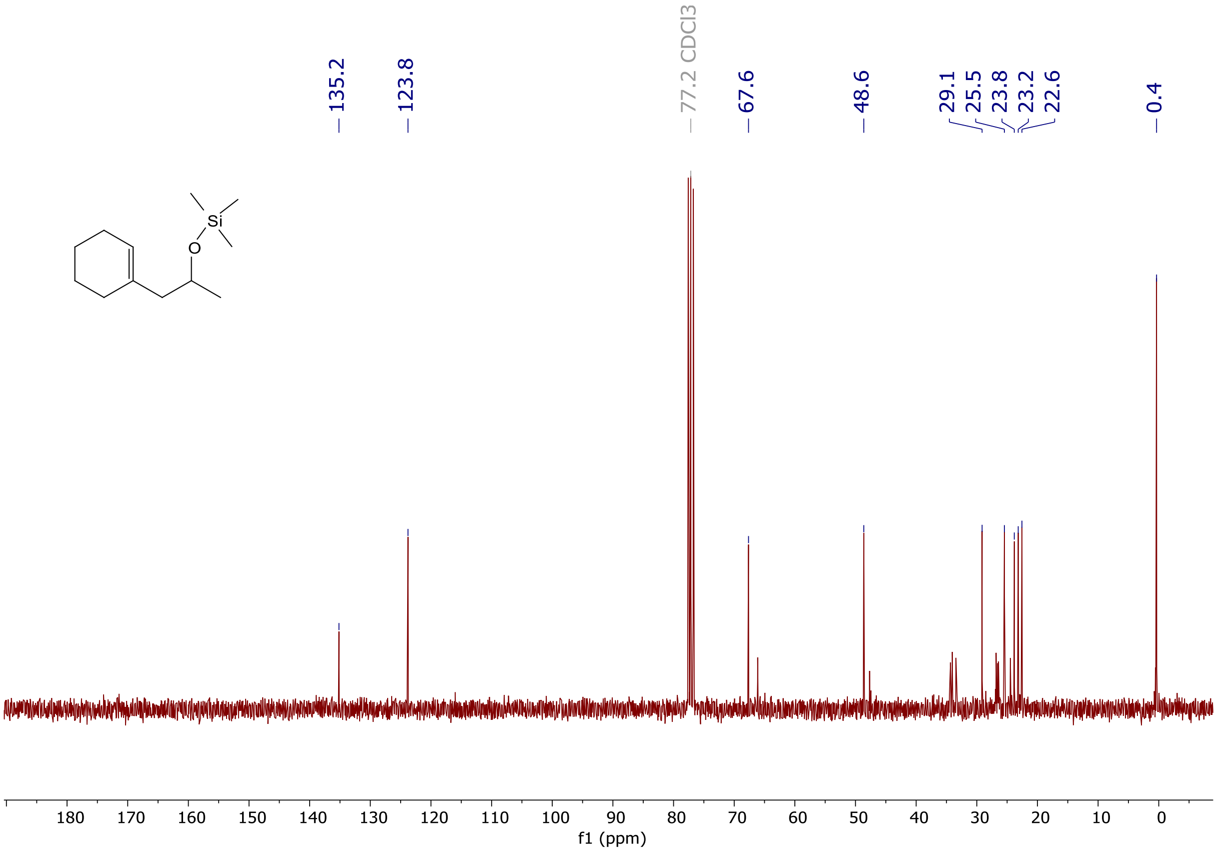

^{13}C NMR Spectrum of **2g (**101MHz, $CDCl_3$)

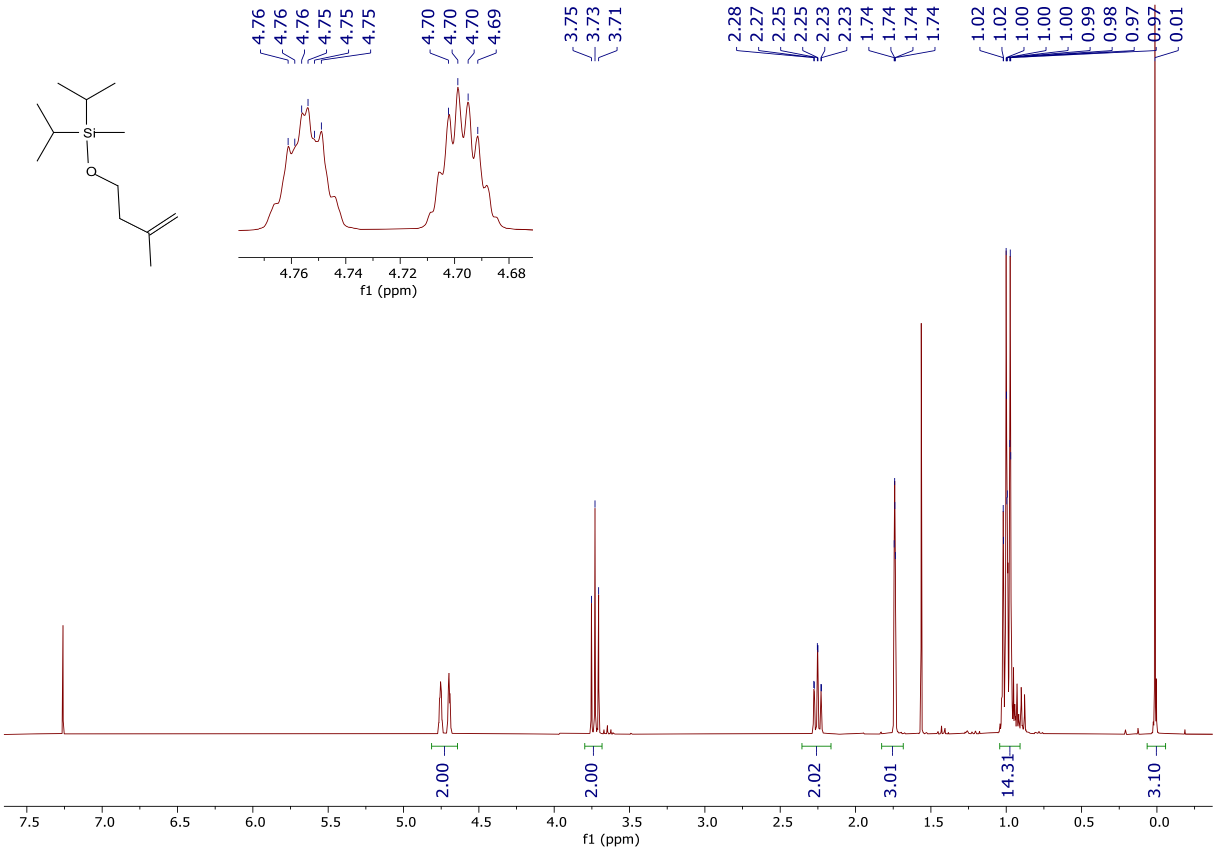

^{1}H NMR Spectrum of **2h** (300MHz, $CDCl_3$)

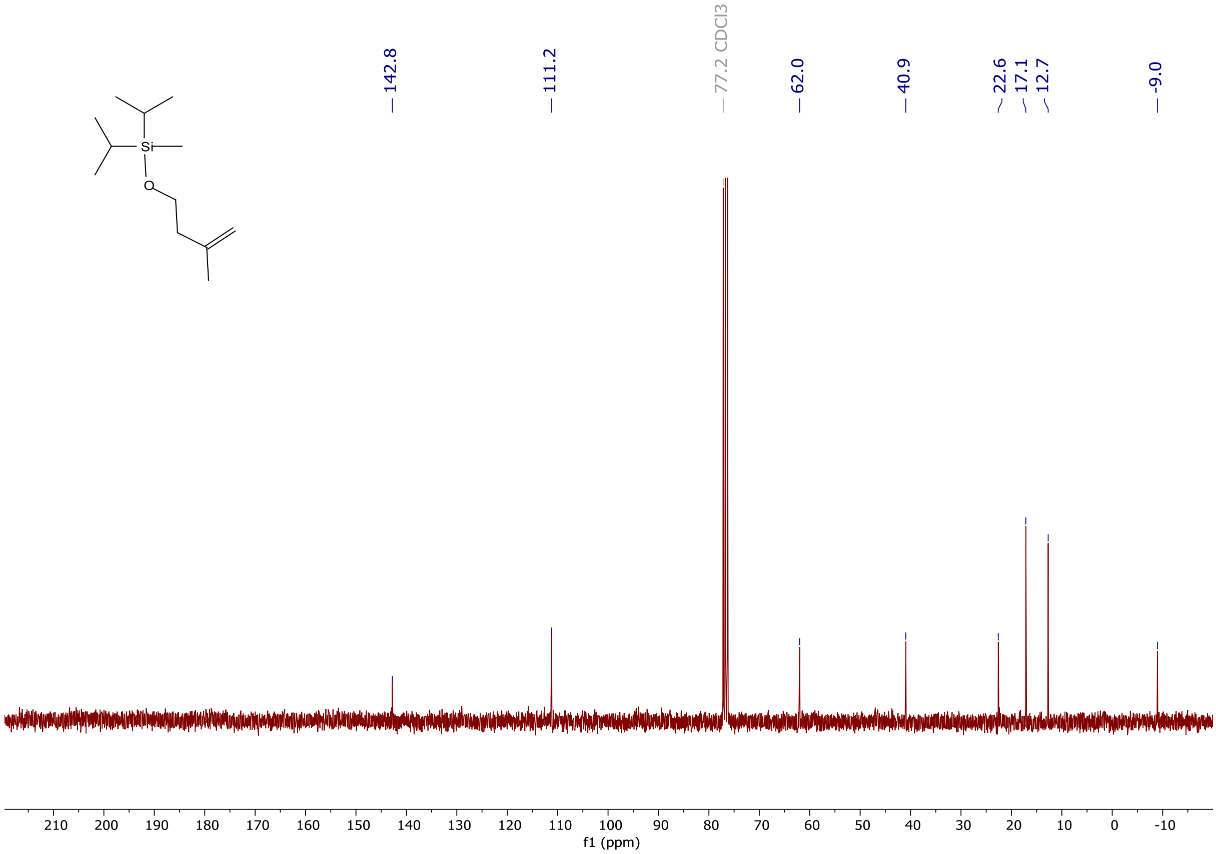

^{13}C NMR Spectrum of **2h (**101MHz, $CDCl_3$)

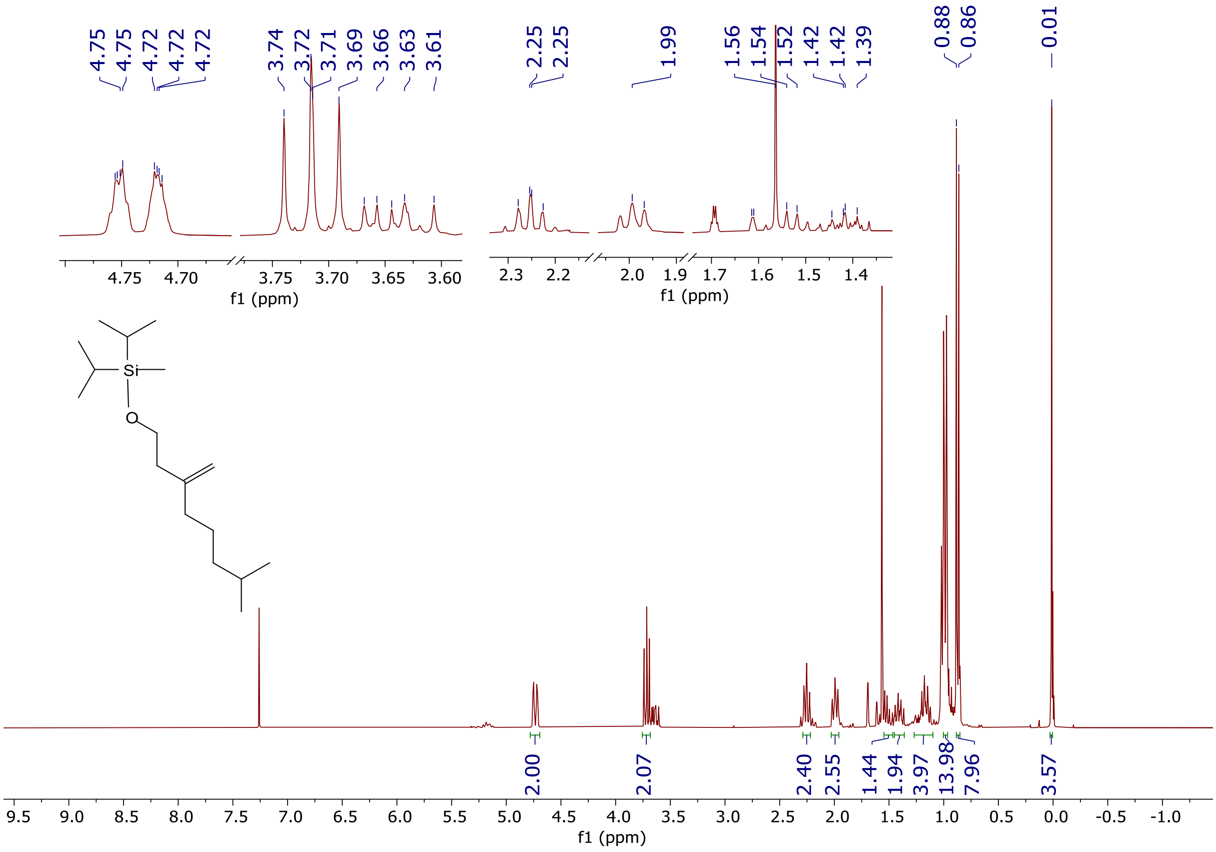

^{1}H NMR Spectrum of **2i** (300MHz, $CDCl_3$, Contains minor amount of hydrodehalogenation byproduct)

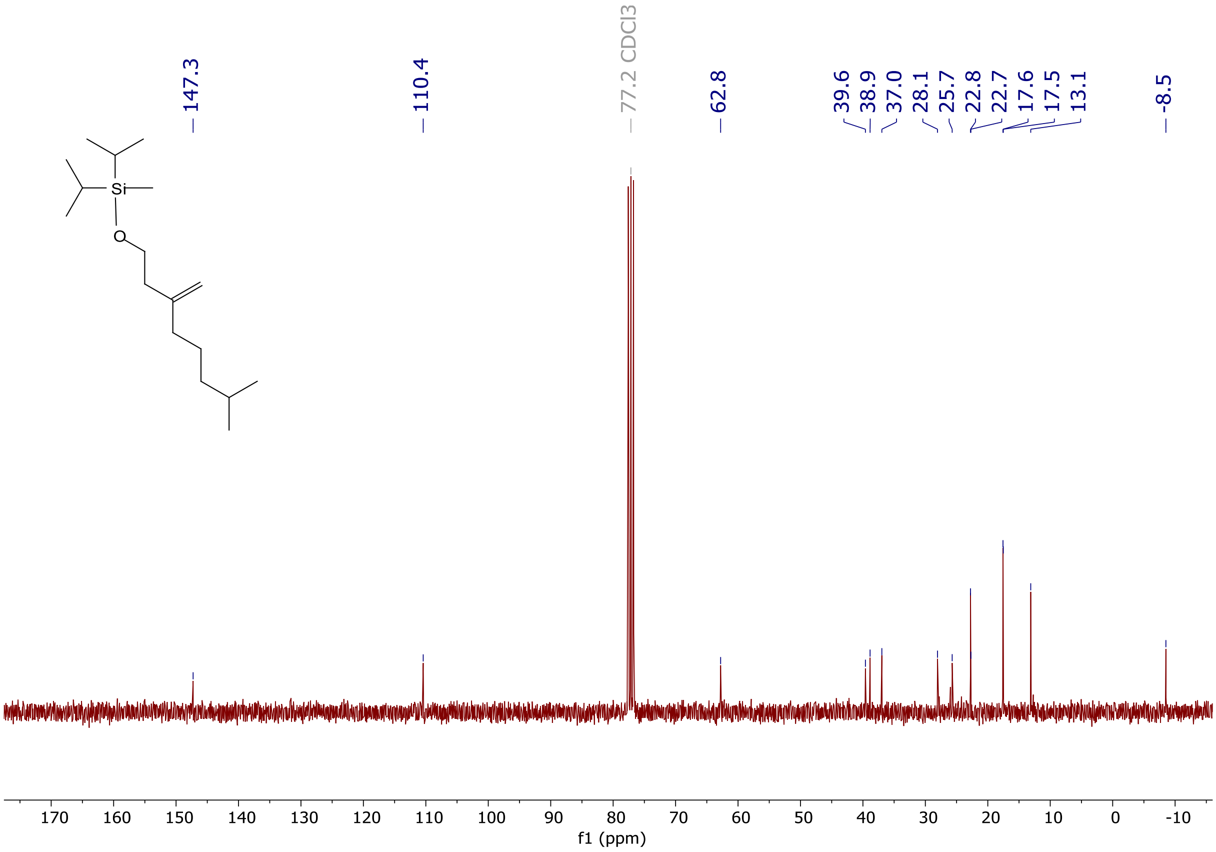

^{13}C NMR Spectrum of **2i** (101MHz, $CDCl_3$)

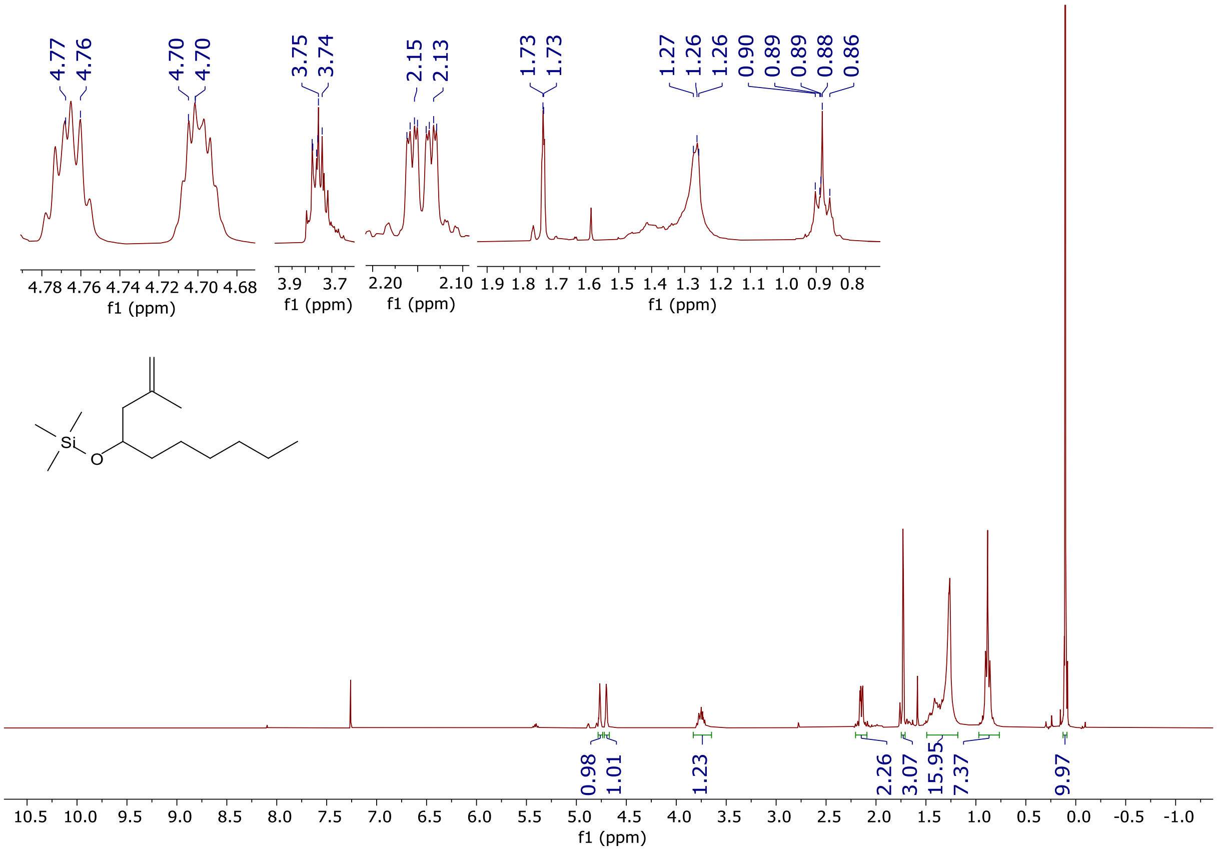

^{1}H NMR Spectrum of **2j** (300MHz, $CDCl_3$)

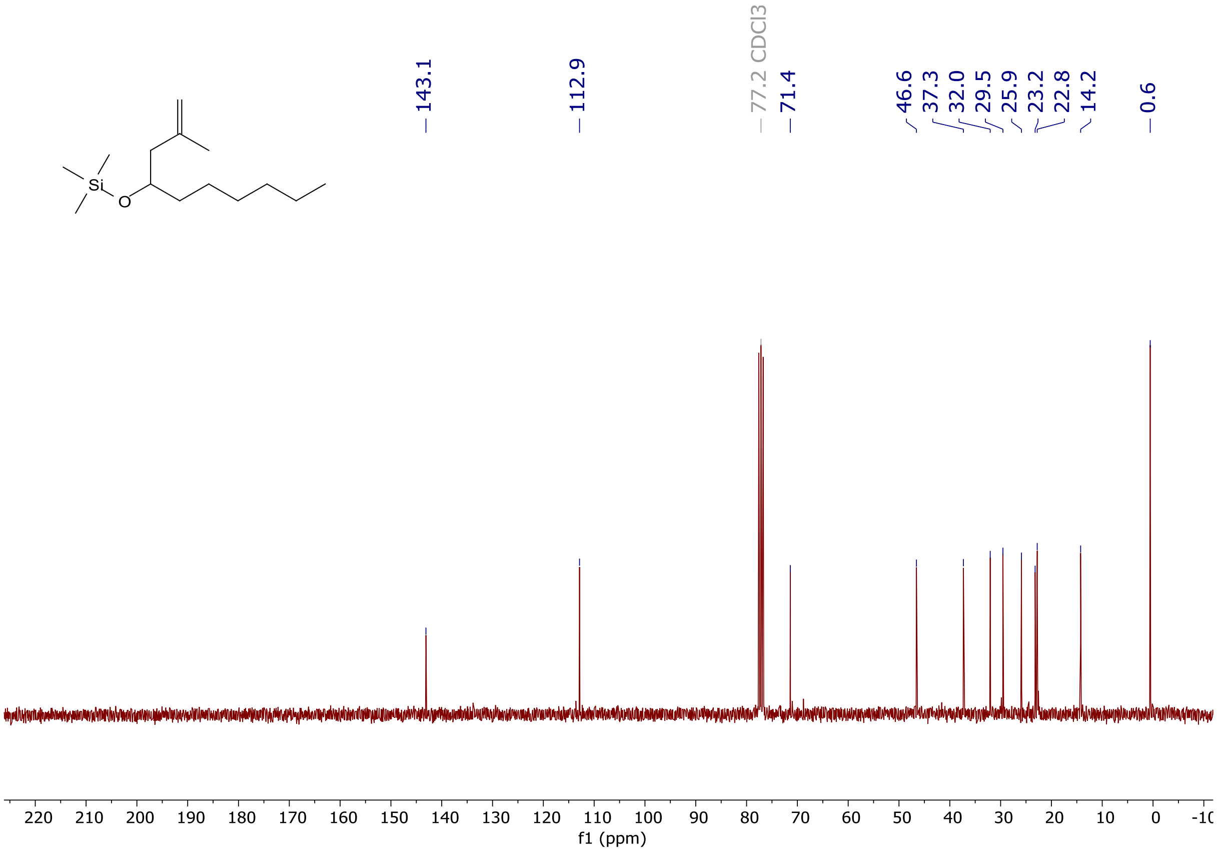

^{13}C NMR Spectrum of **2j** (101MHz, $CDCl_3$)

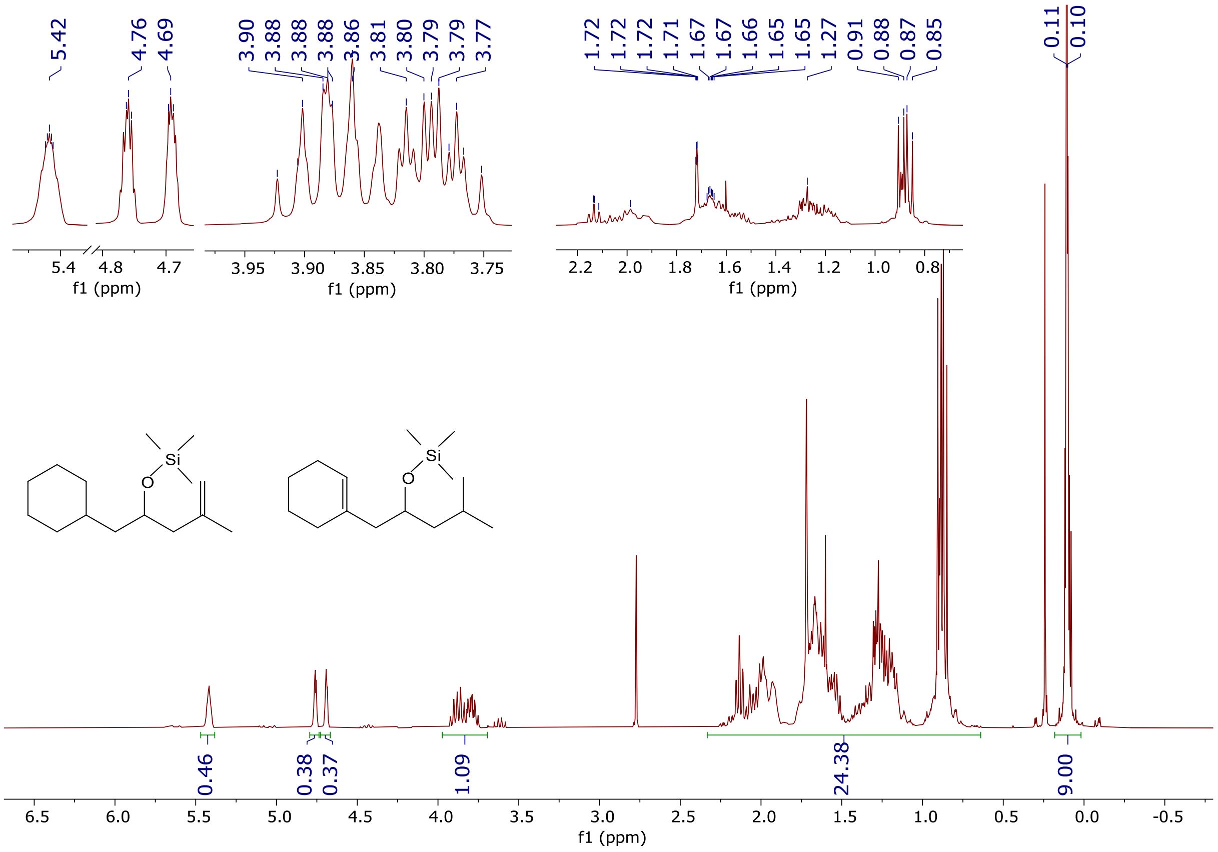

^{1}H NMR Spectrum of **2k** (300MHz, $CDCl_3$)

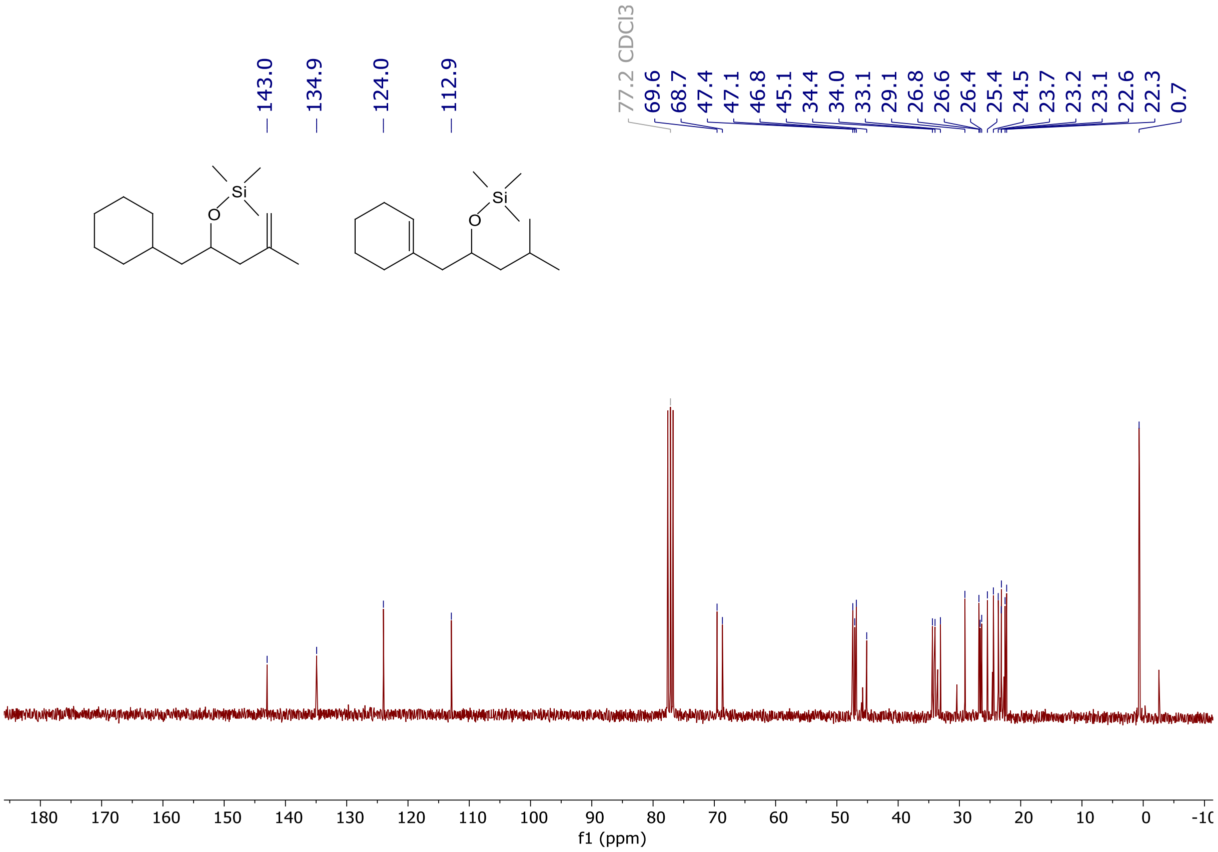

^{13}C NMR Spectrum of **2k (**101MHz, $CDCl_3$)

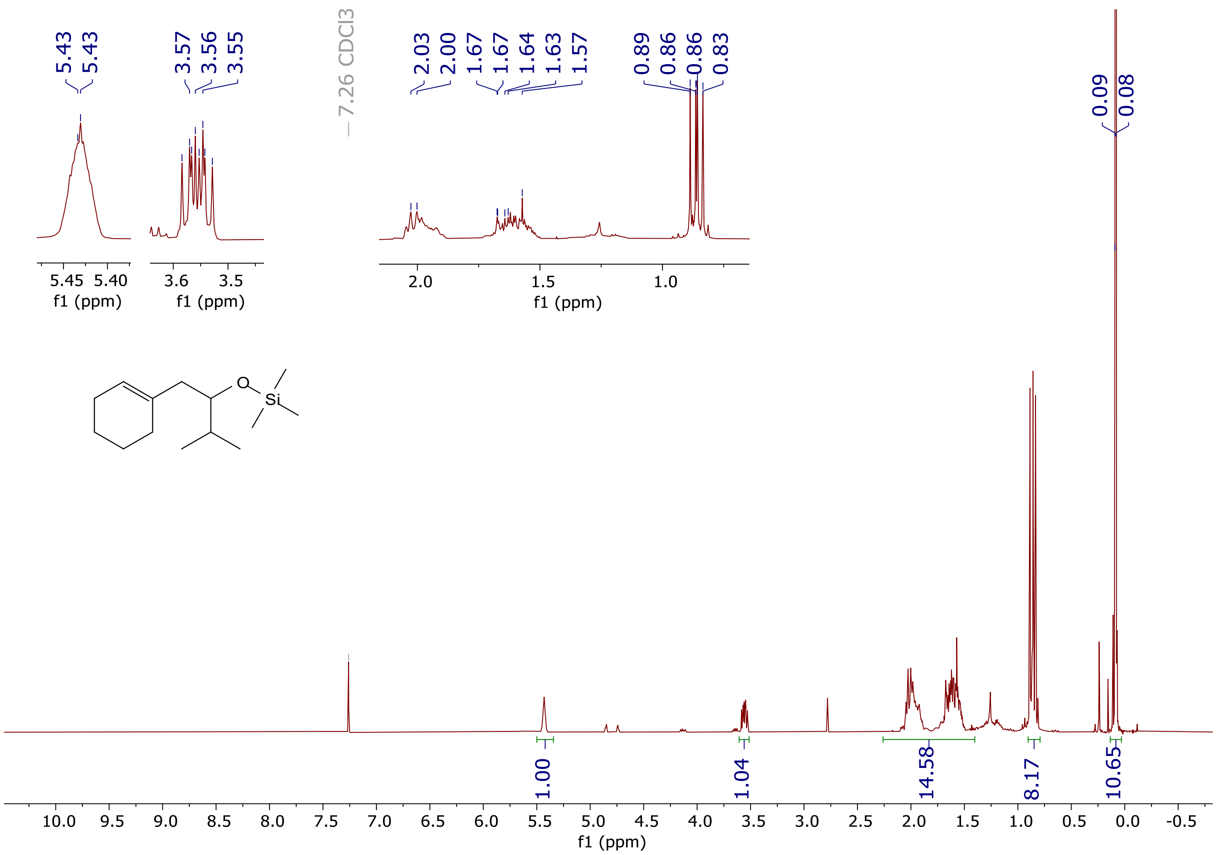

^{1}H NMR Spectrum of **2l** (300MHz, $CDCl_3$)

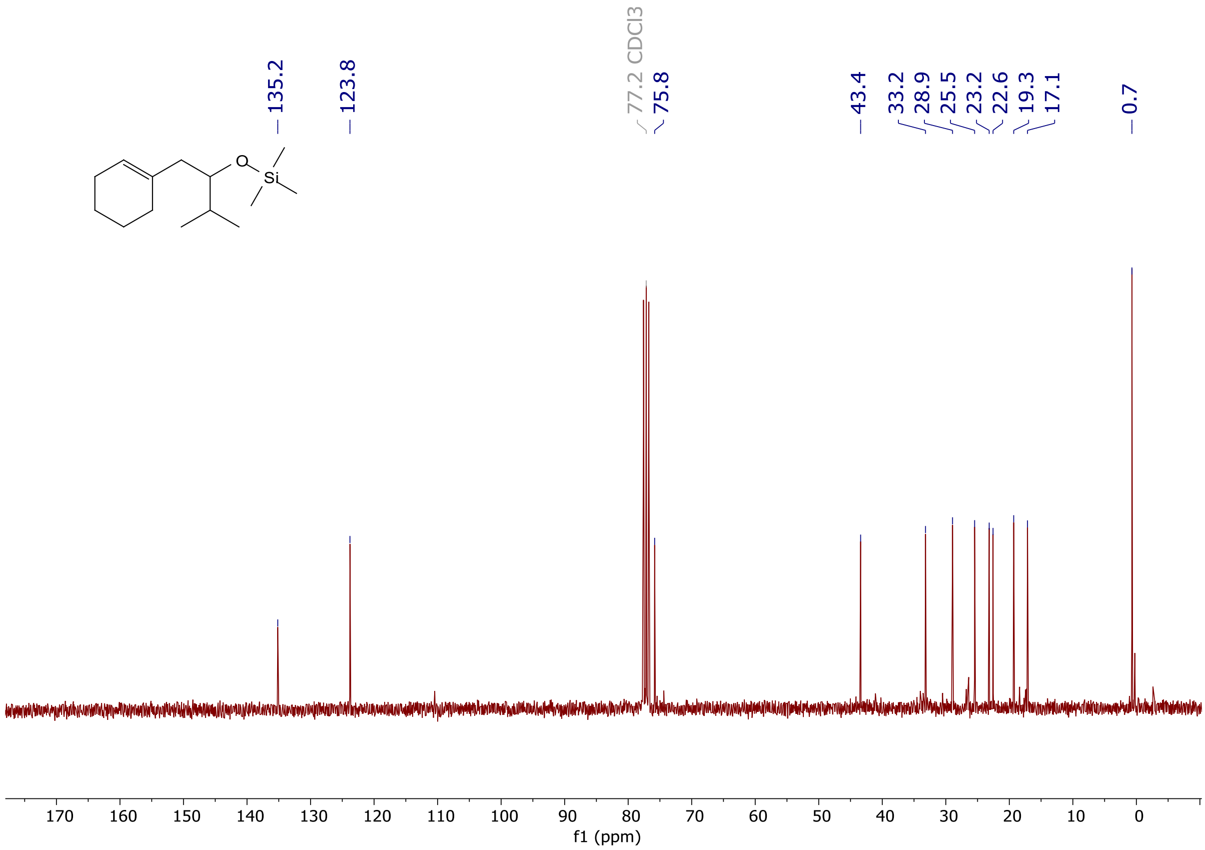

^{13}C NMR Spectrum of **2l** (101MHz, $CDCl_3$)

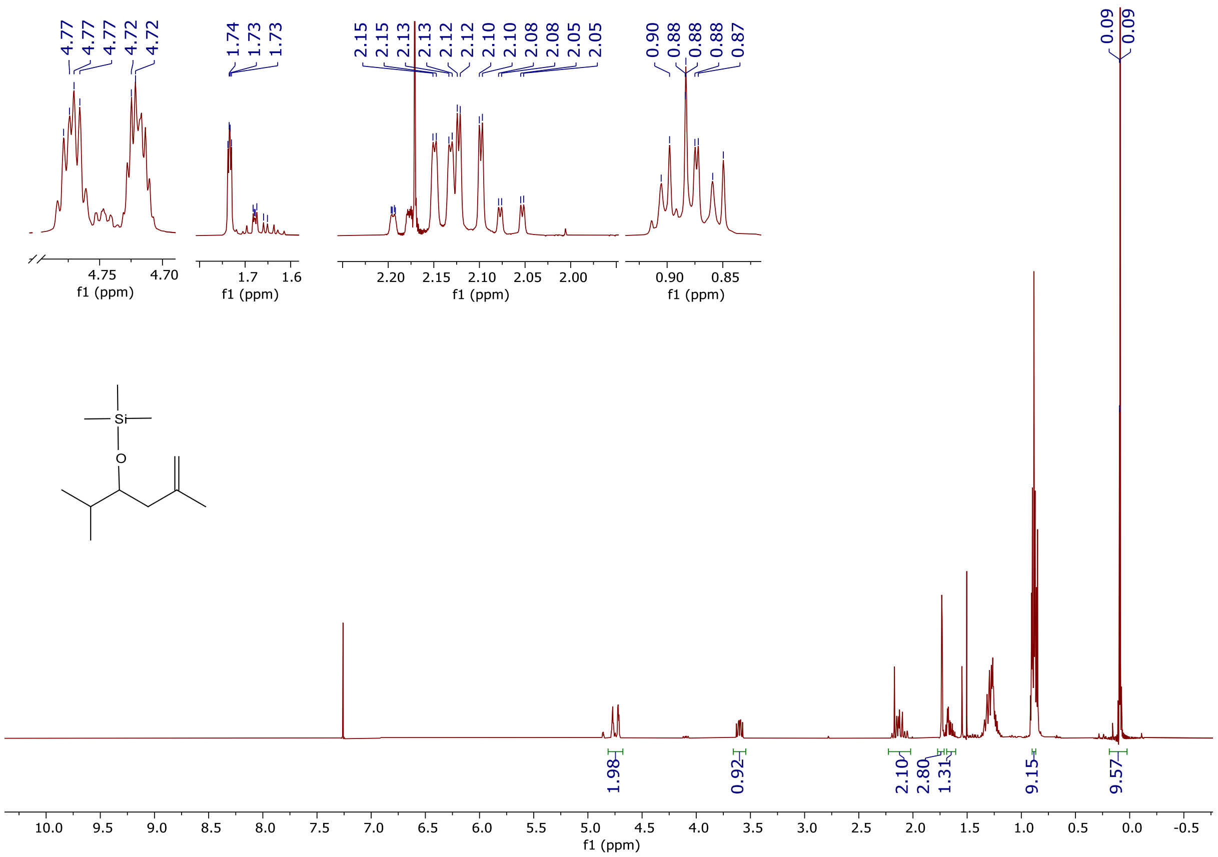

^{1}H NMR Spectrum of **2m** (300MHz, $CDCl_3$)

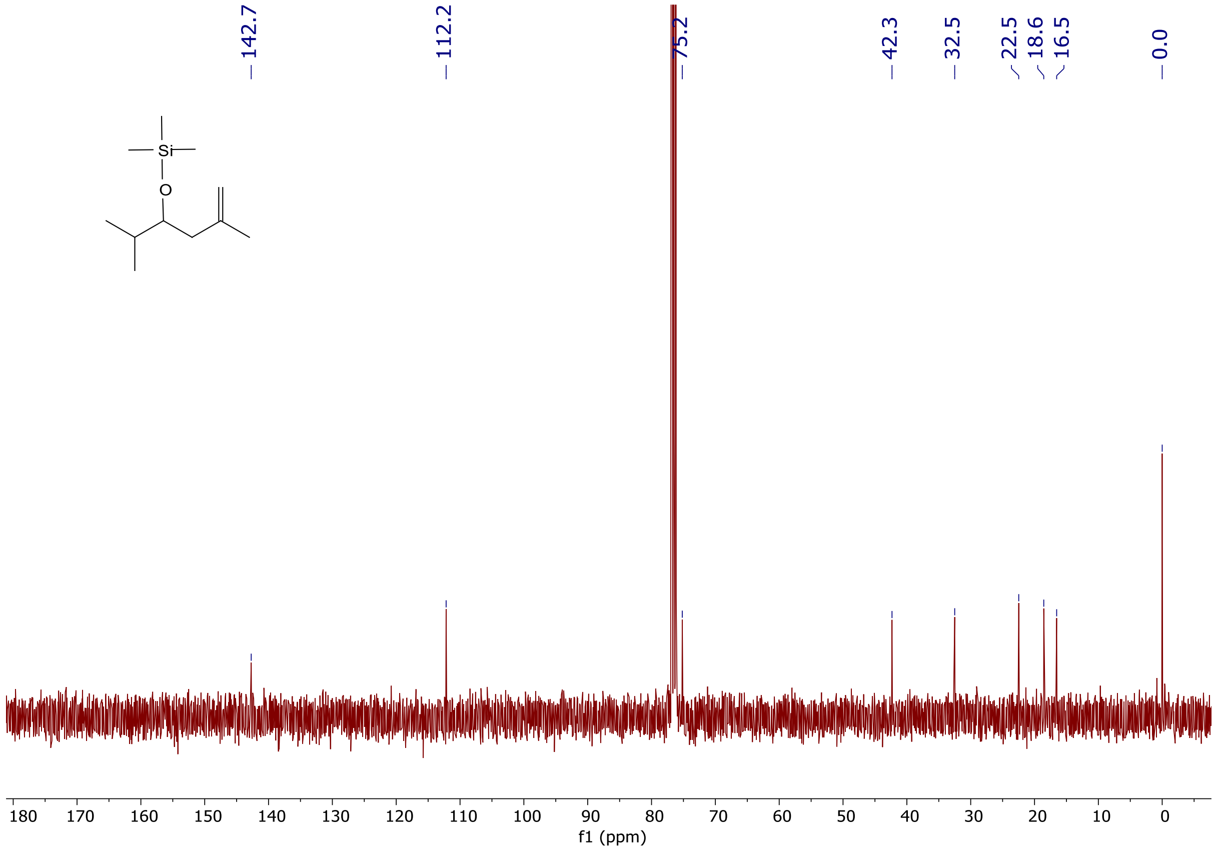

^{13}C NMR Spectrum of **2m** (101MHz, $CDCl_3$)

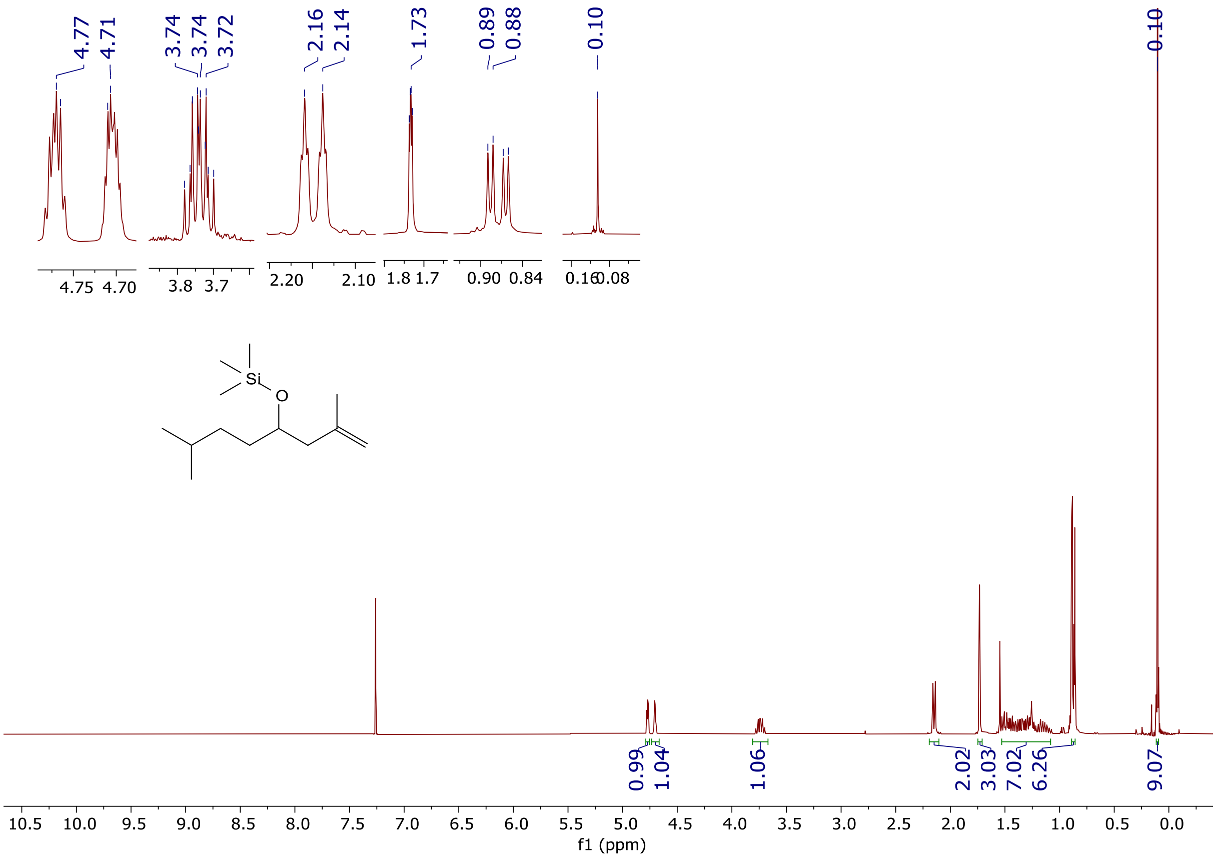

^{1}H NMR Spectrum of **2n** (300MHz, $CDCl_3$)

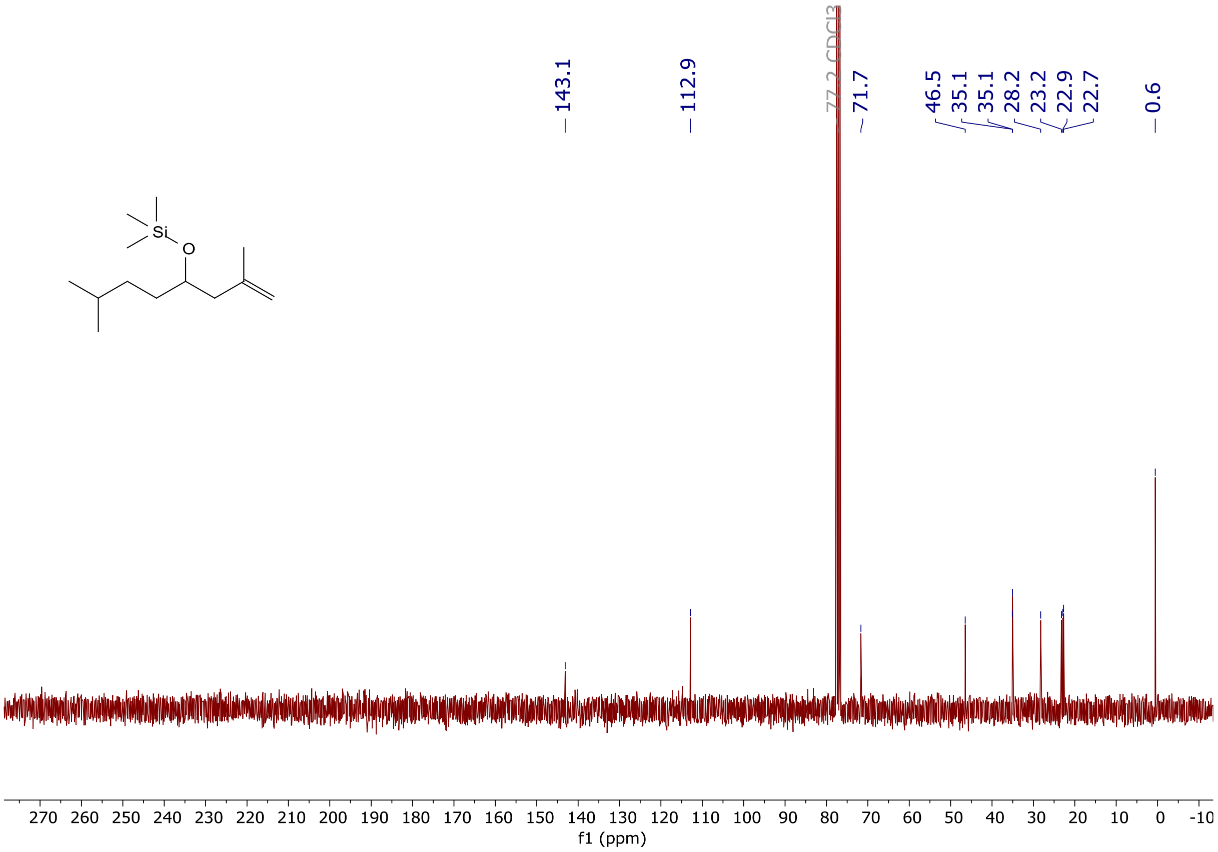

^{13}C NMR Spectrum of **2n** (101MHz, $CDCl_3$)

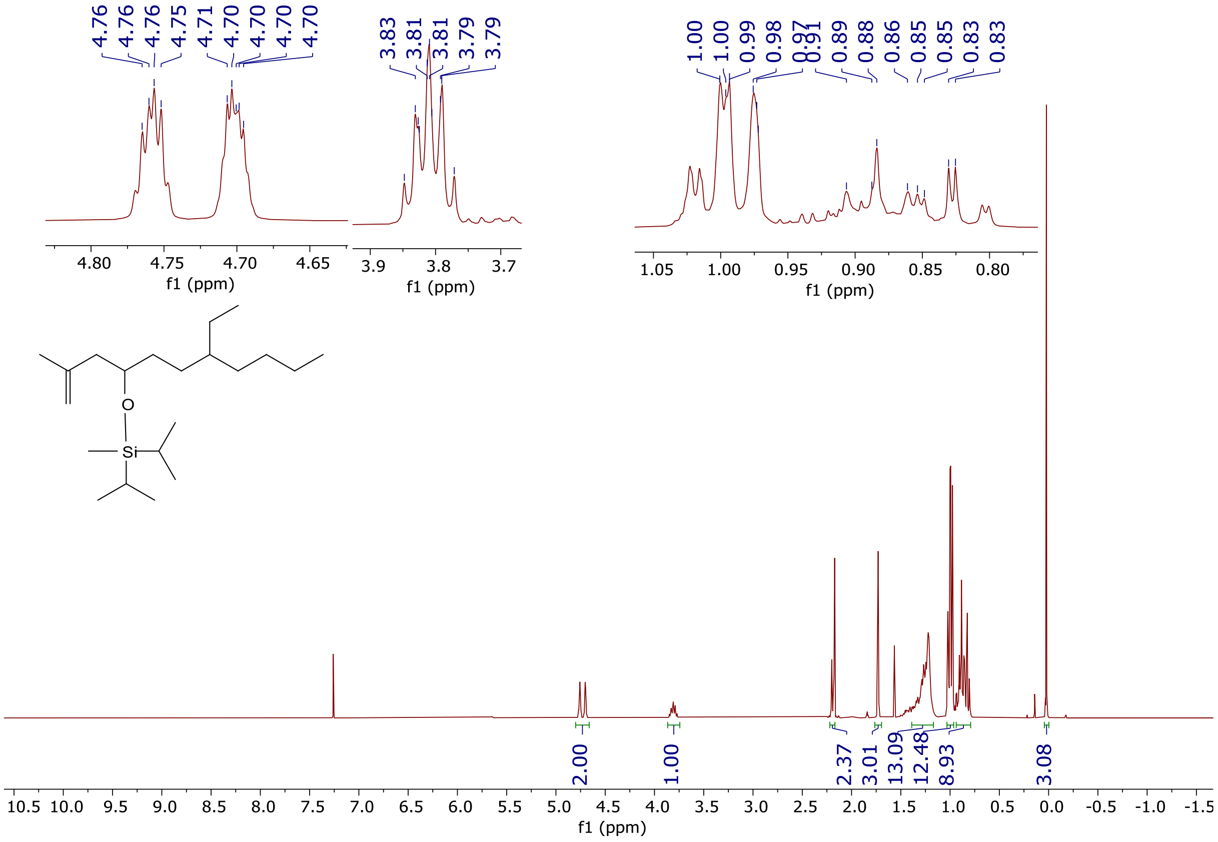

^{1}H NMR Spectrum of **2o** (300MHz, $CDCl_3$)

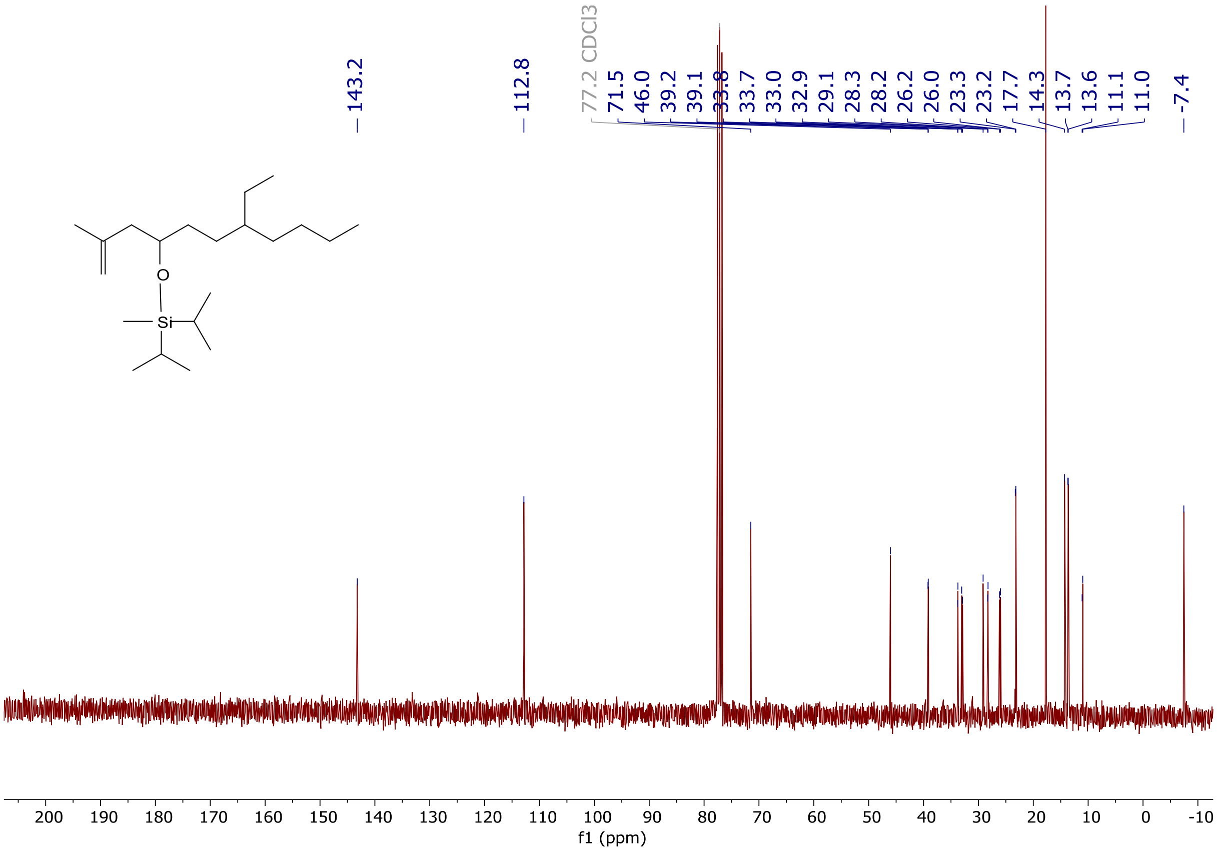

^{13}C NMR Spectrum of **2o** (101MHz, $CDCl_3$)

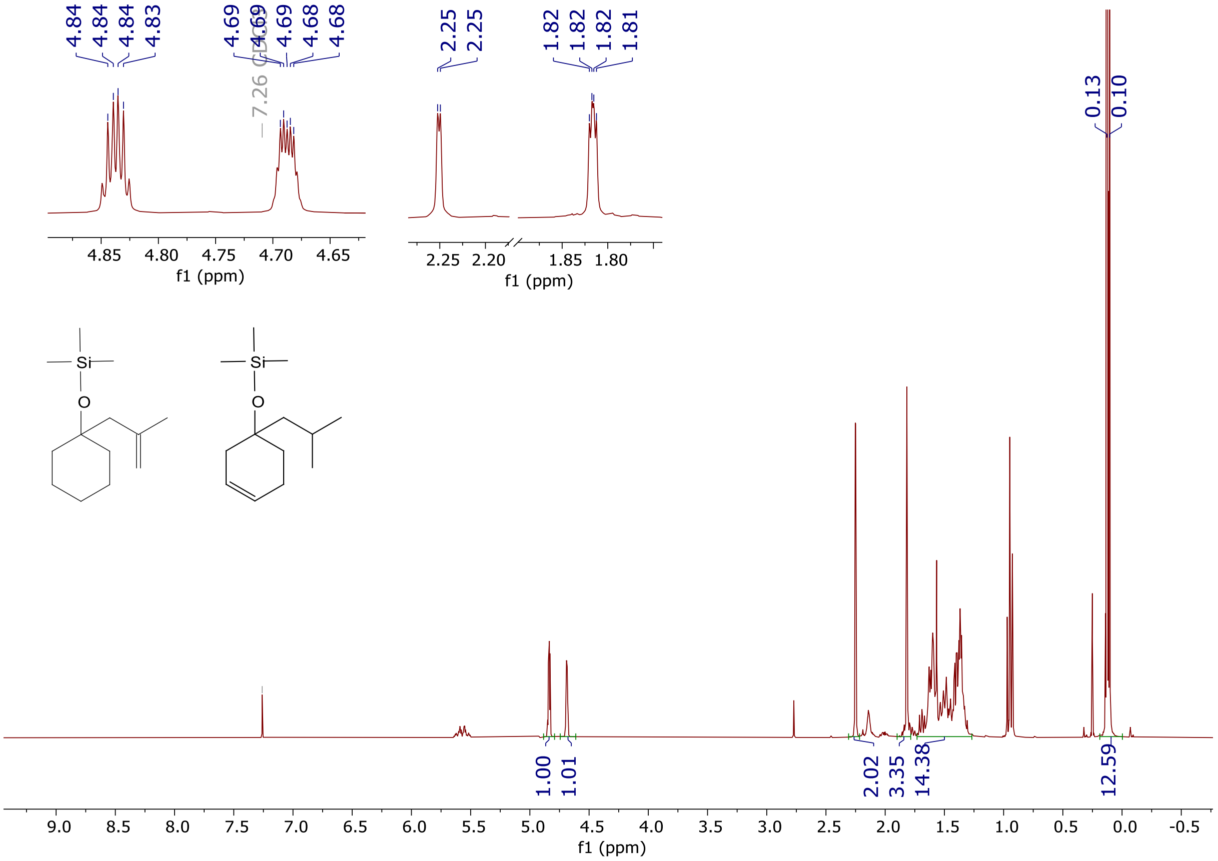

^{1}H NMR Spectrum of **2p** (300MHz, $CDCl_3$, only the peak of the major product is integrated)

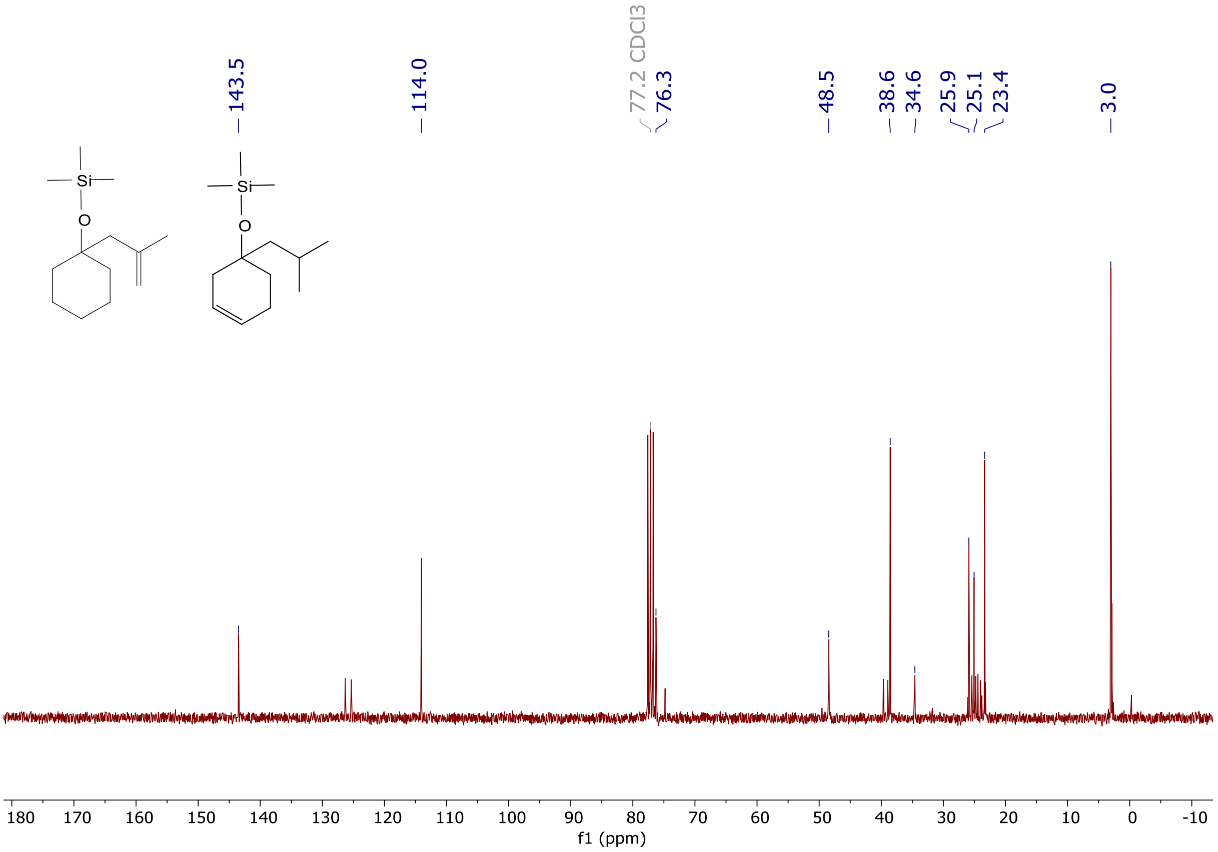

^{13}C NMR Spectrum of **2p** (101MHz, $CDCl_3$)

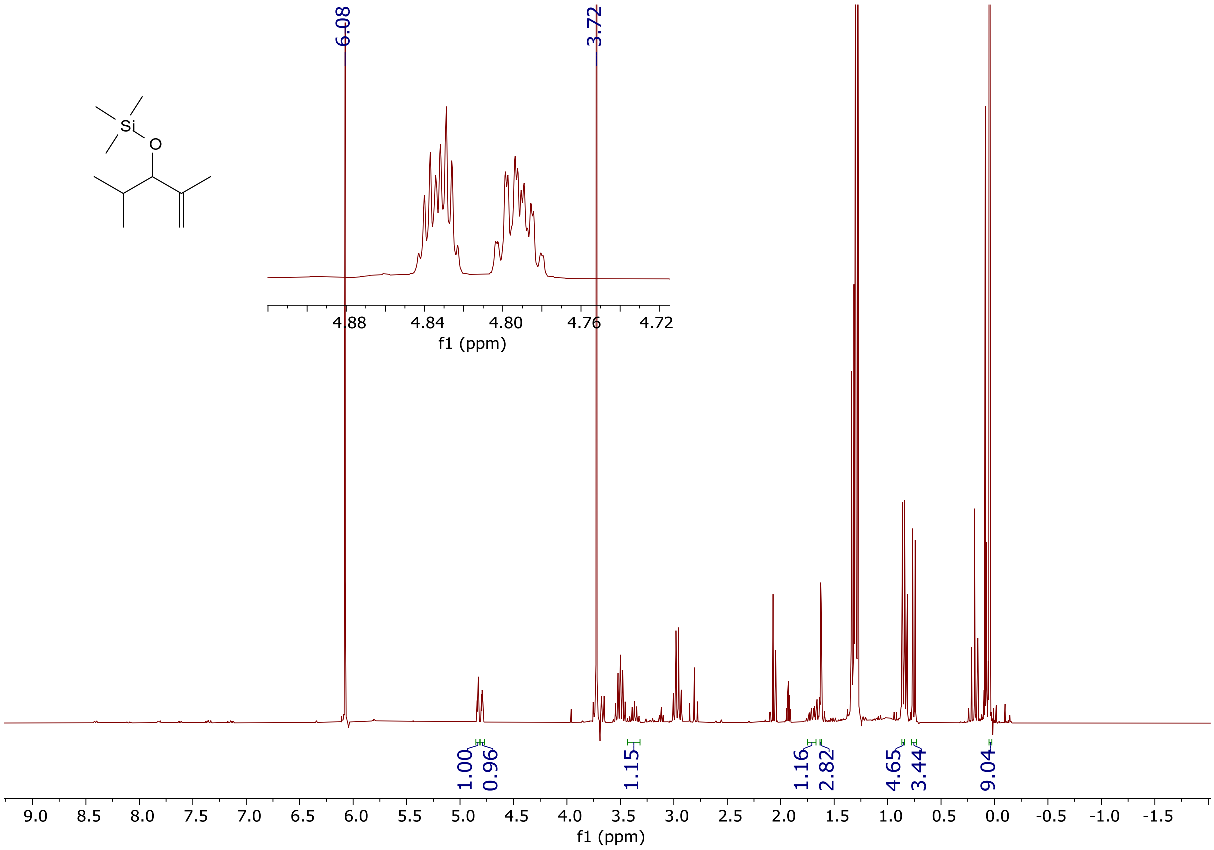

Crude ^{1}H NMR Spectrum of **2q** (300MHz, CD_3CN, with internal standard 1,3,5-trimethoxybenzene)

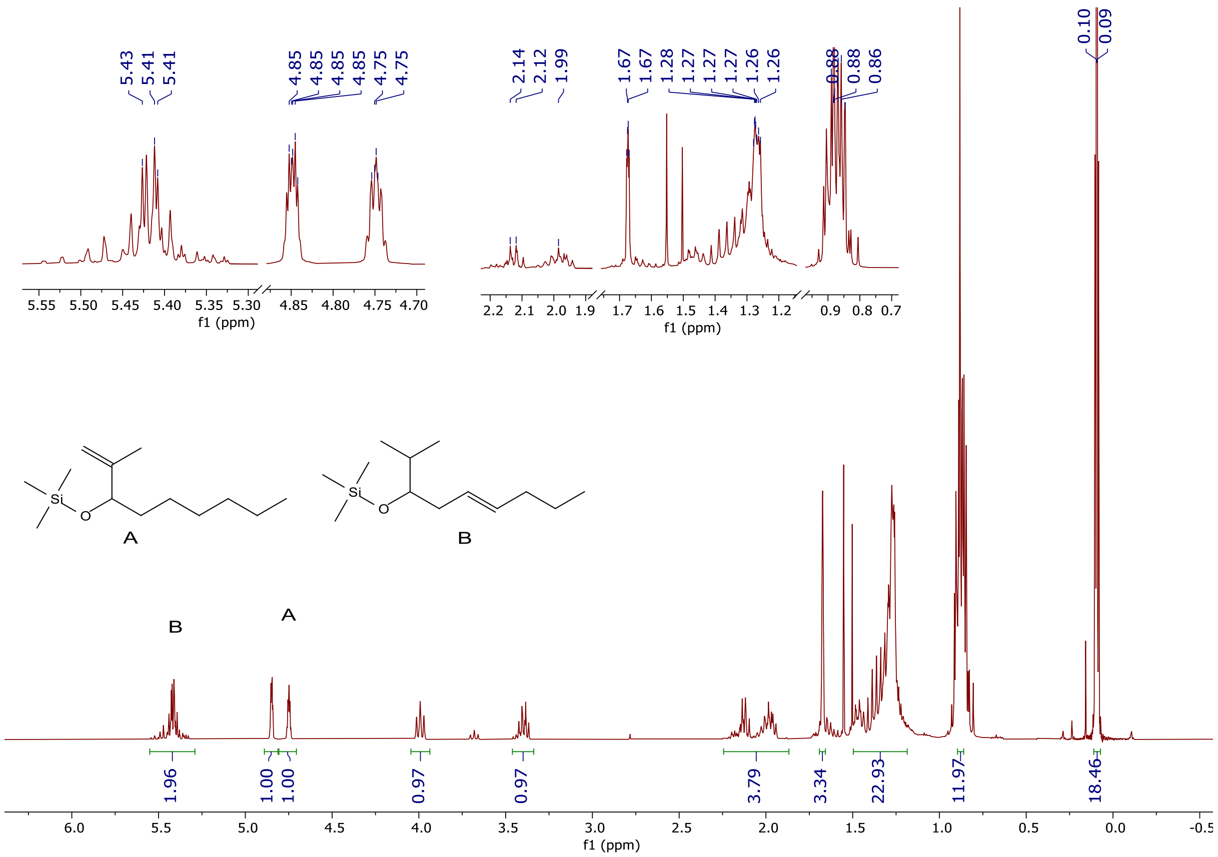

^{1}H NMR Spectrum of **2r** (300MHz, $CDCl_3$)

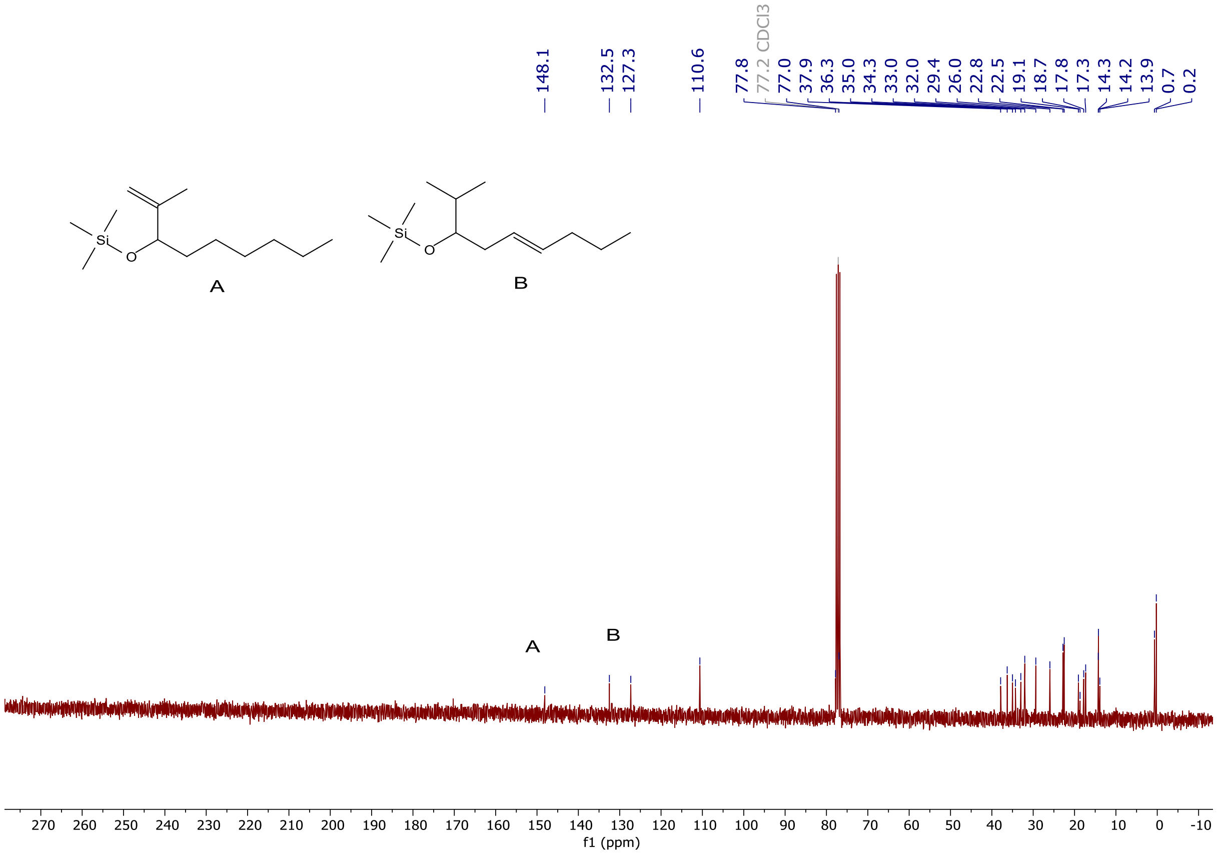

^{13}C NMR Spectrum of **2r** (101MHz, $CDCl_3$)

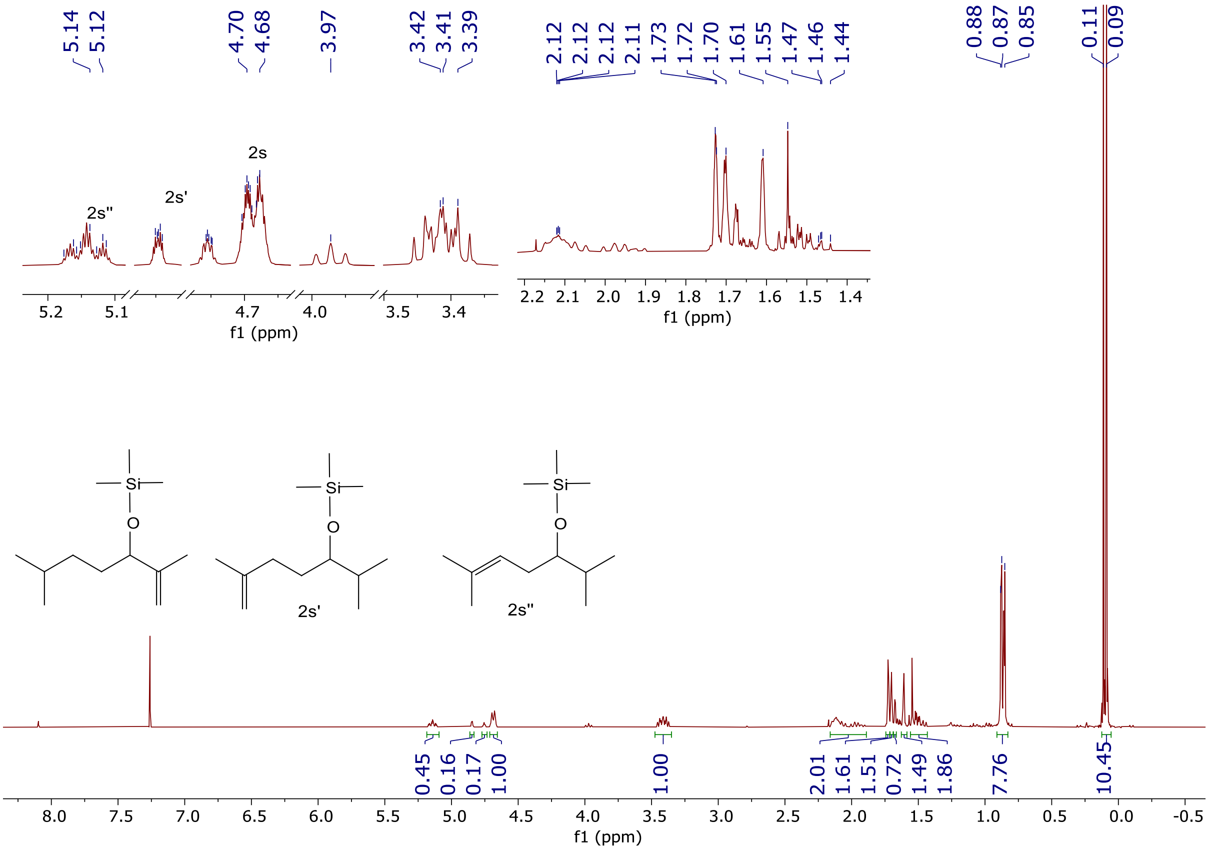

^{1}H NMR Spectrum of **2s** (300MHz, $CDCl_3$)

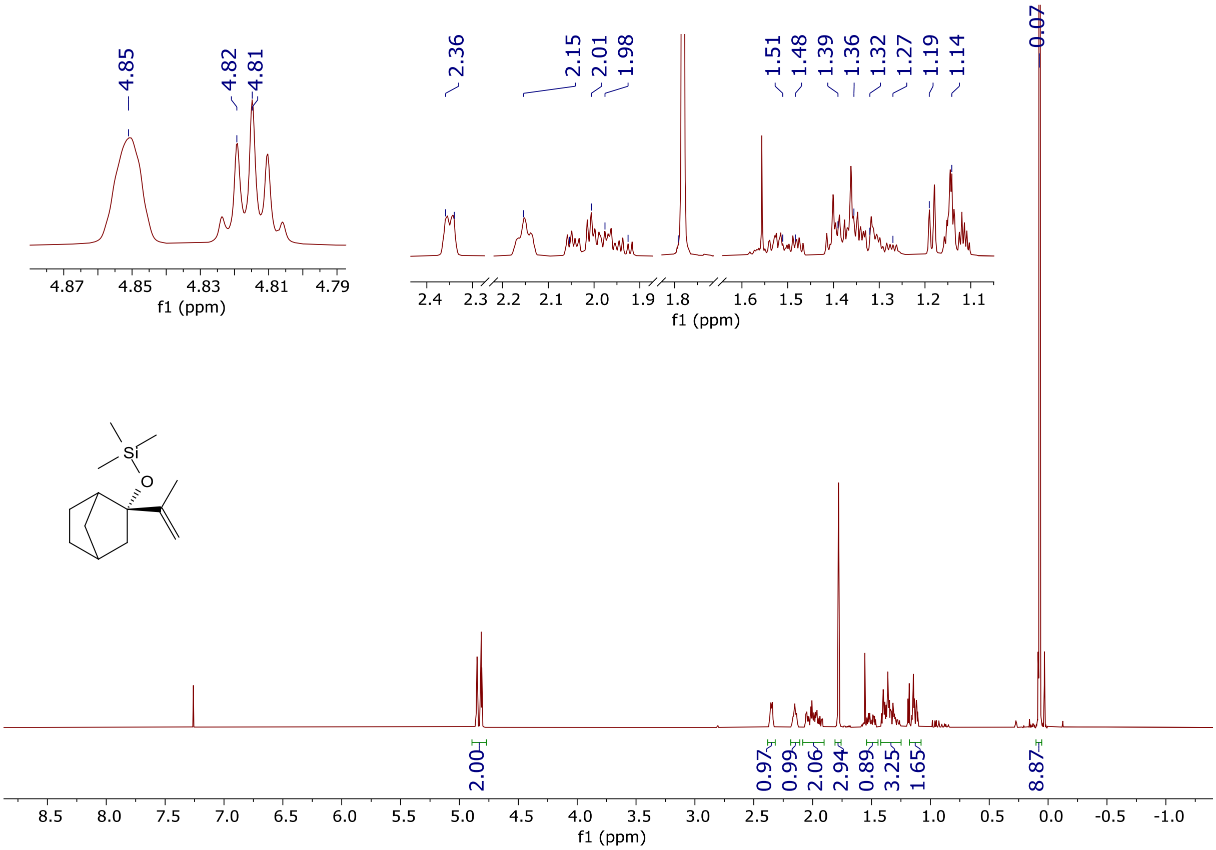

^{1}H NMR Spectrum of **2t** (300MHz, $CDCl_3$)

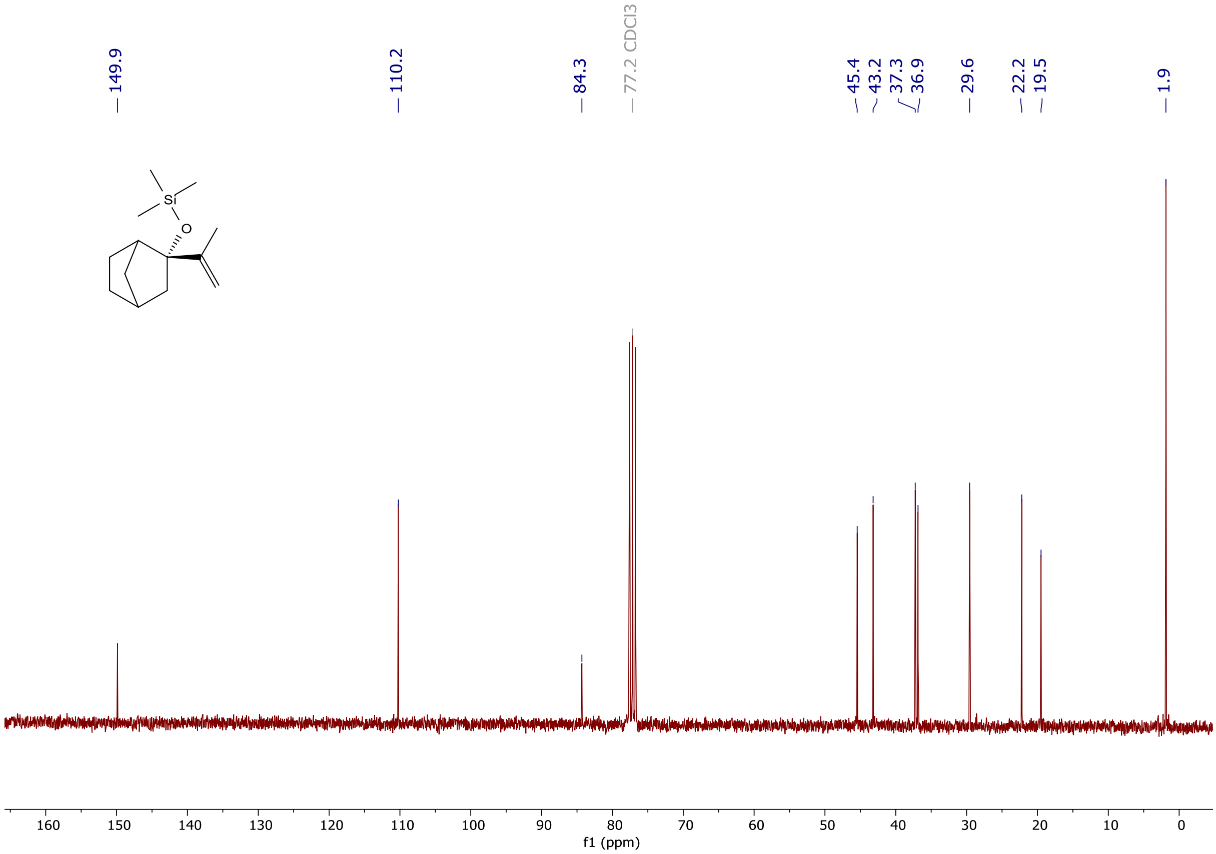

^{13}C NMR Spectrum of **2t** (101MHz, $CDCl_3$)

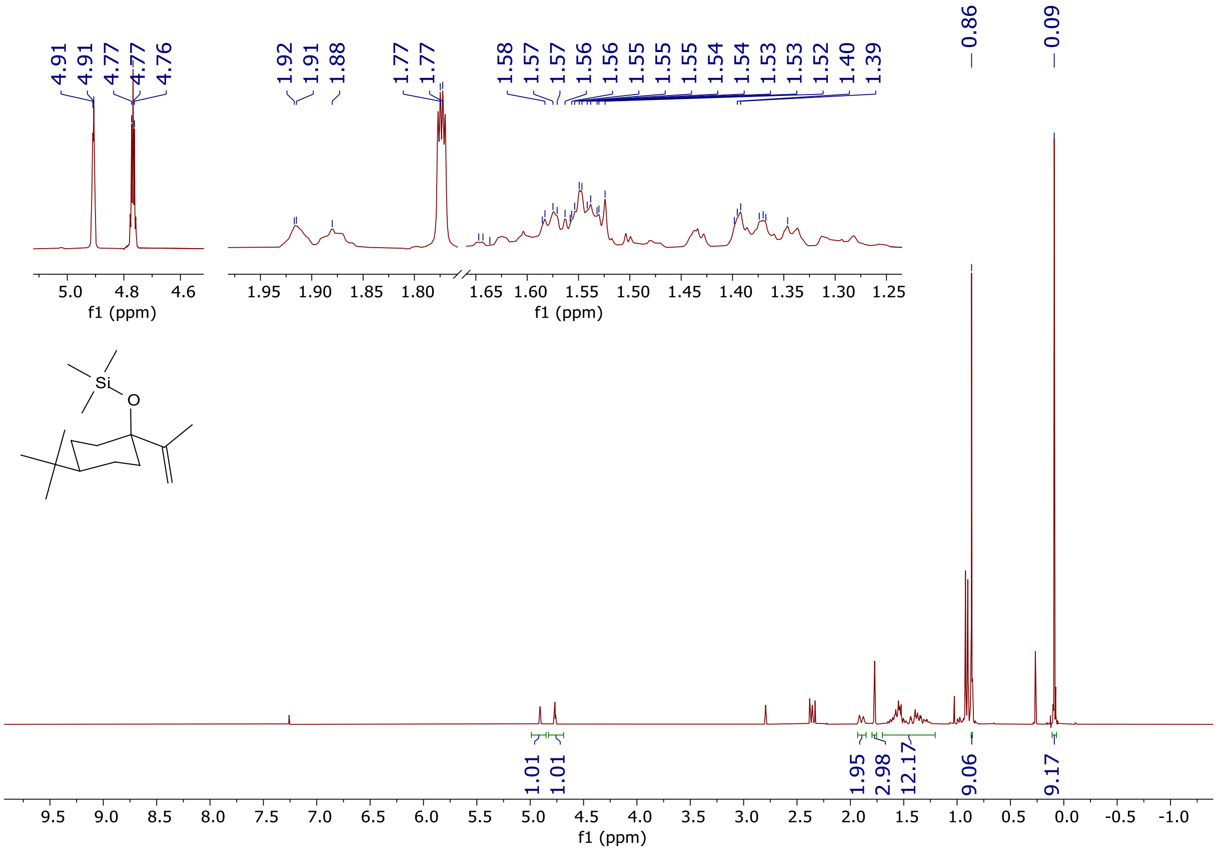

^{1}H NMR Spectrum of **2u** (300MHz, $CDCl_3$, Contains minor amount of hydrodehalogenation byproduct)

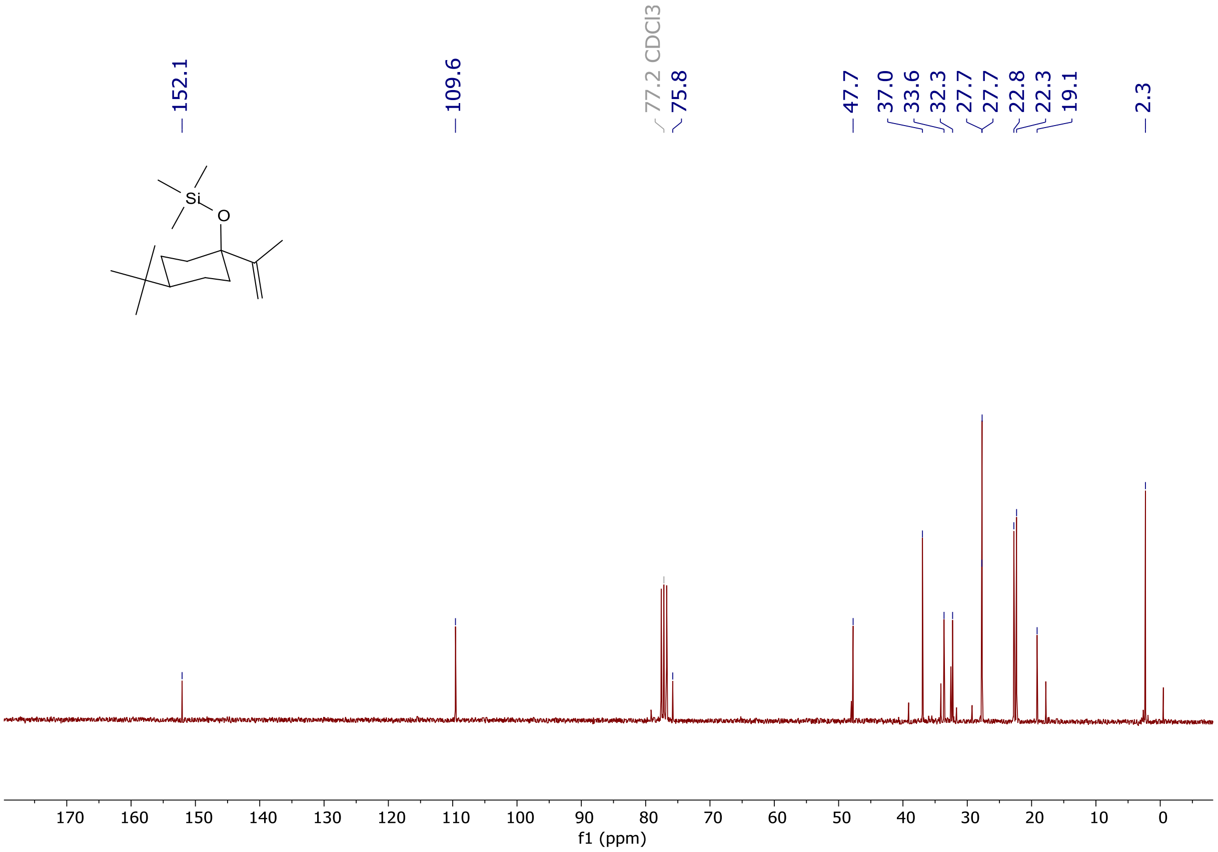

^{13}C NMR Spectrum of **2u** (101MHz, $CDCl_3$)

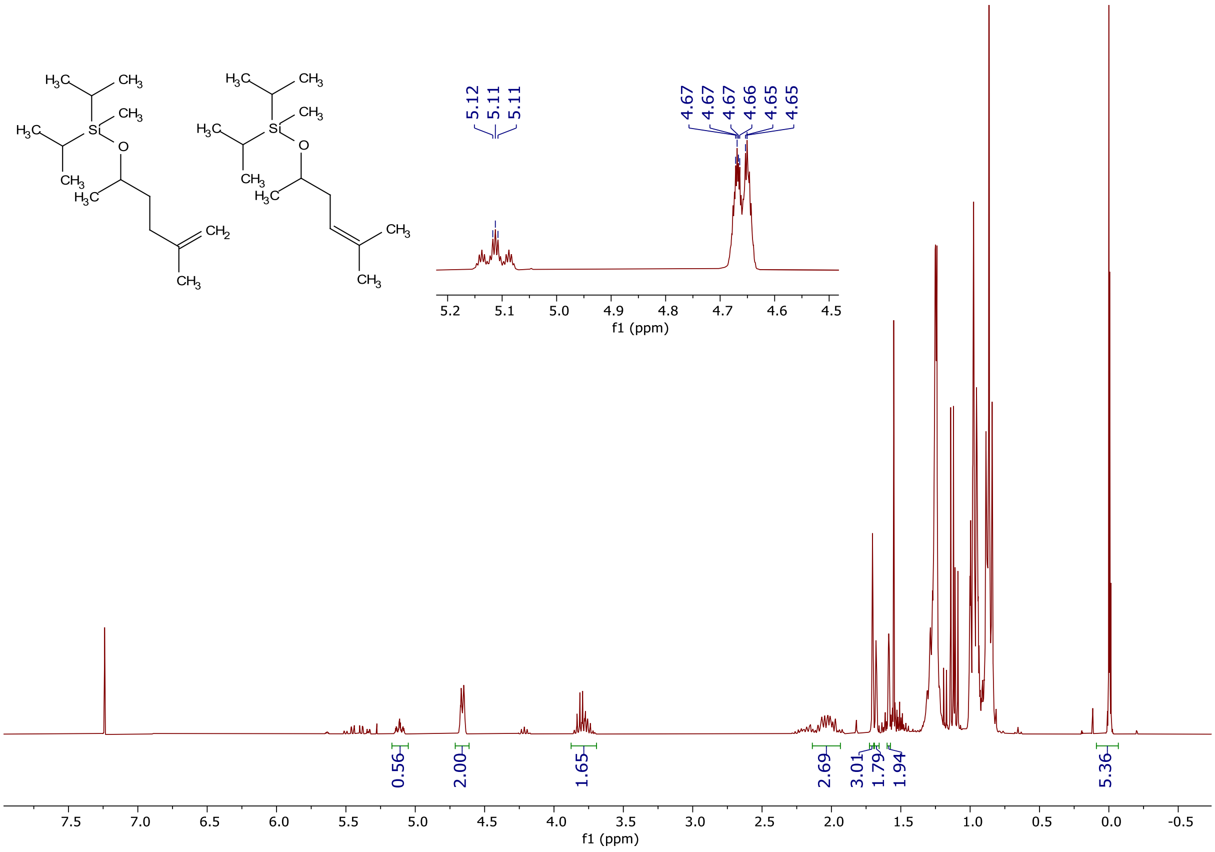

^{1}H NMR Spectrum of **2v** (300MHz, $CDCl_3$)

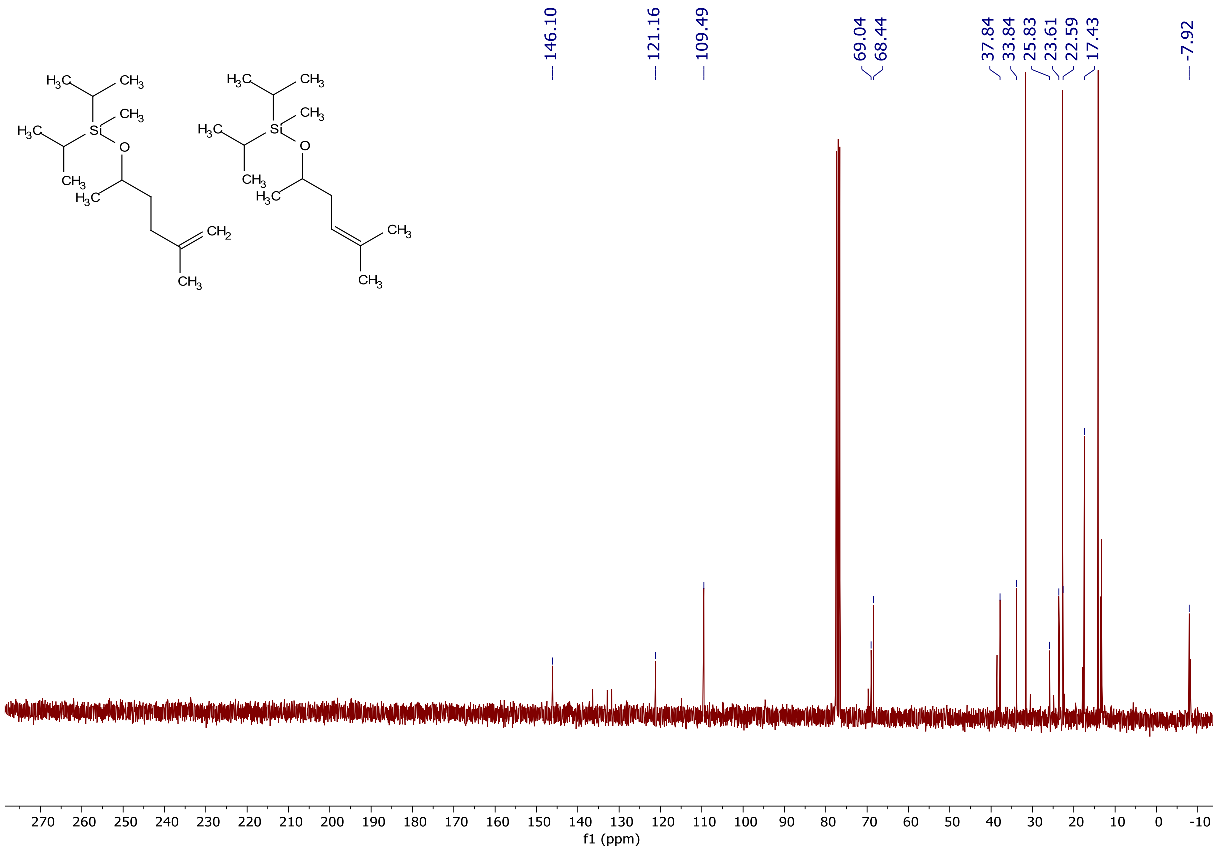

^{13}C NMR Spectrum of **2v** (101MHz, $CDCl_3$)

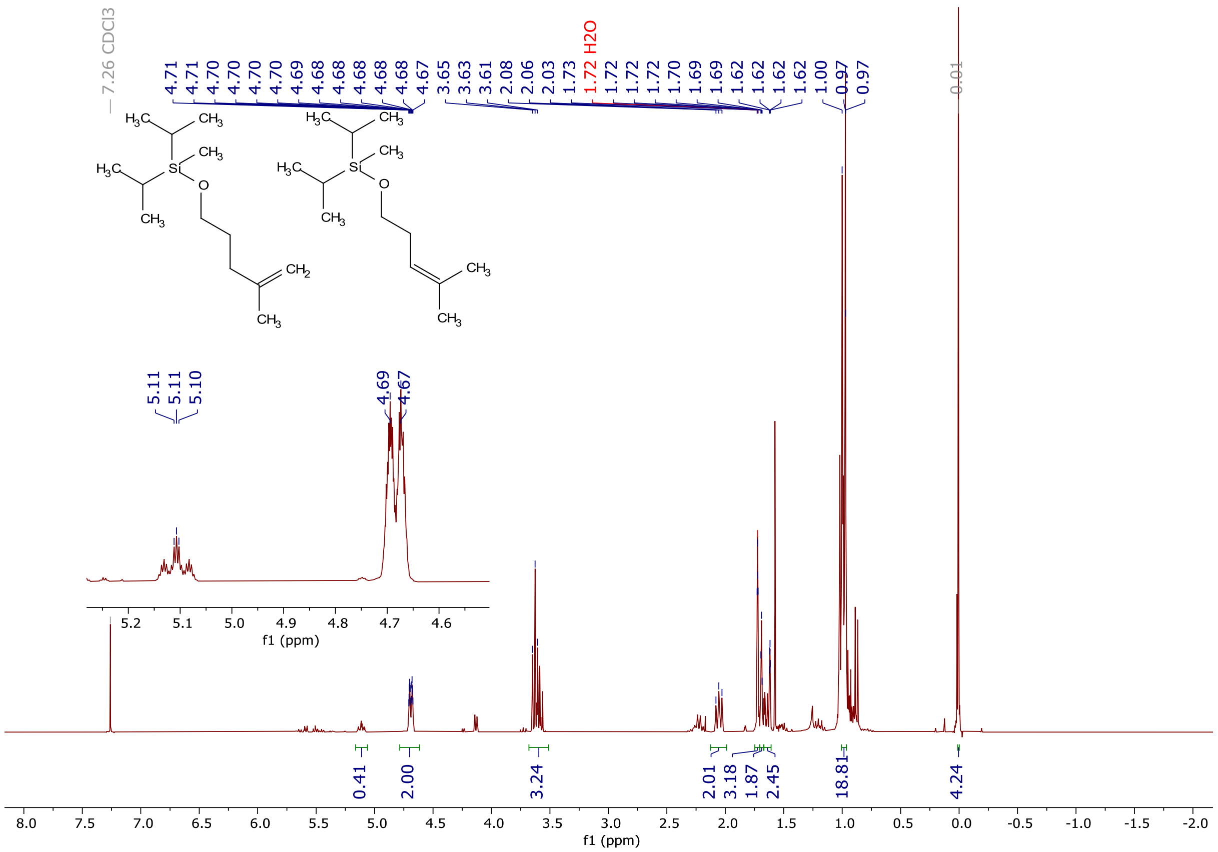

^{1}H NMR Spectrum of **2w** (300MHz, $CDCl_3$)

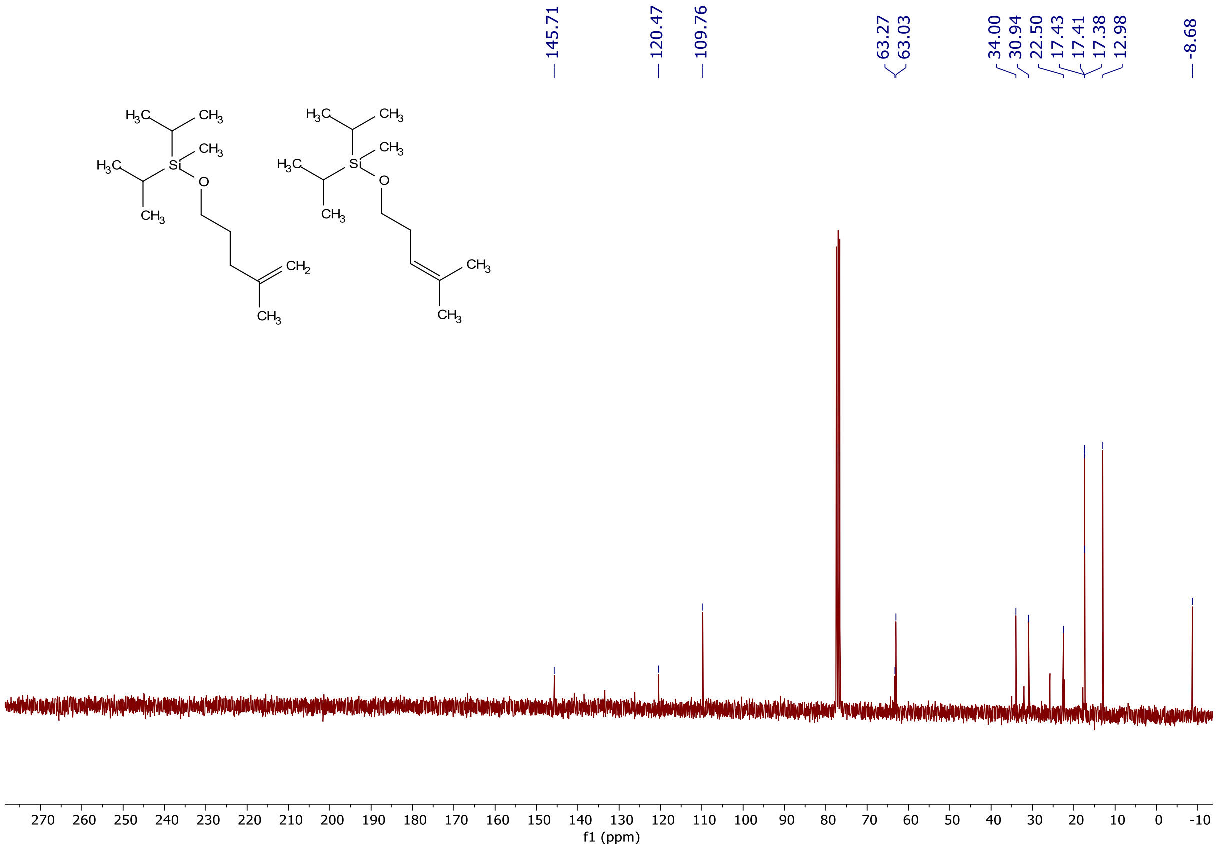

^{13}C NMR Spectrum of **2w (**101MHz, $CDCl_3$)

[1]H Spectrum of **3b**

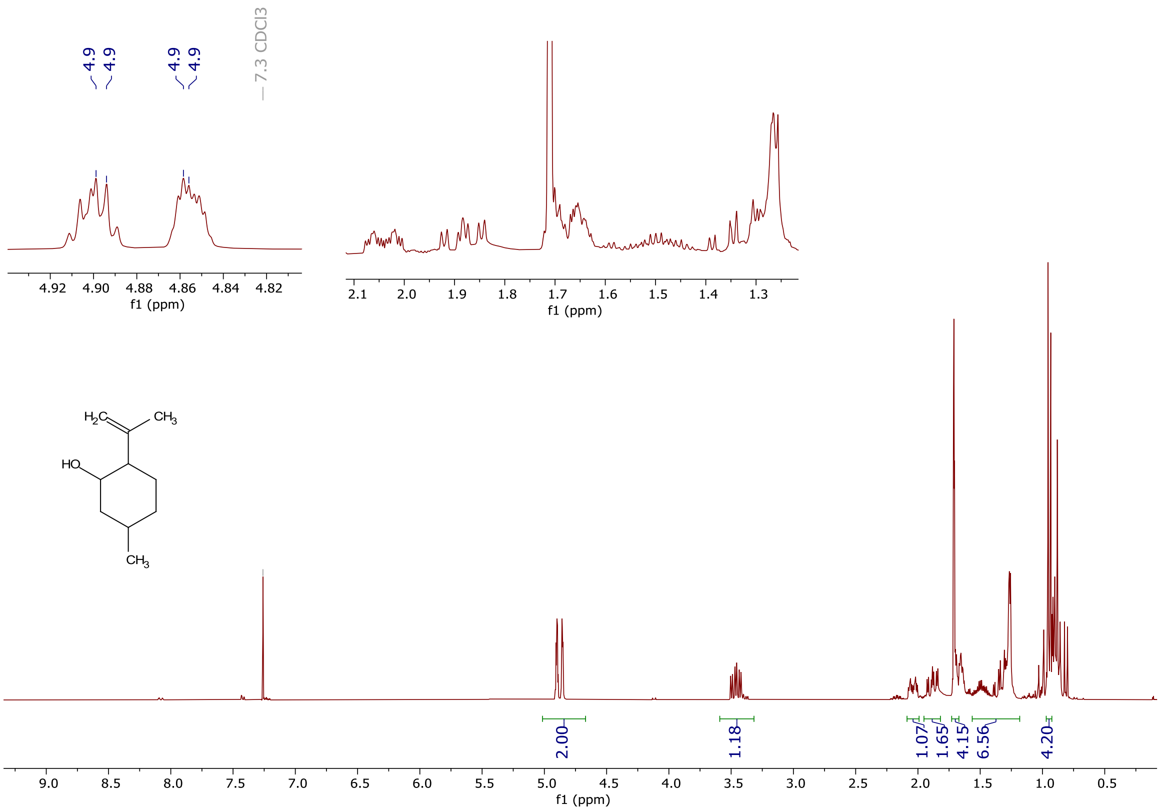

^{1}H NMR Spectrum of **3b** (300MHz, $CDCl_3$)

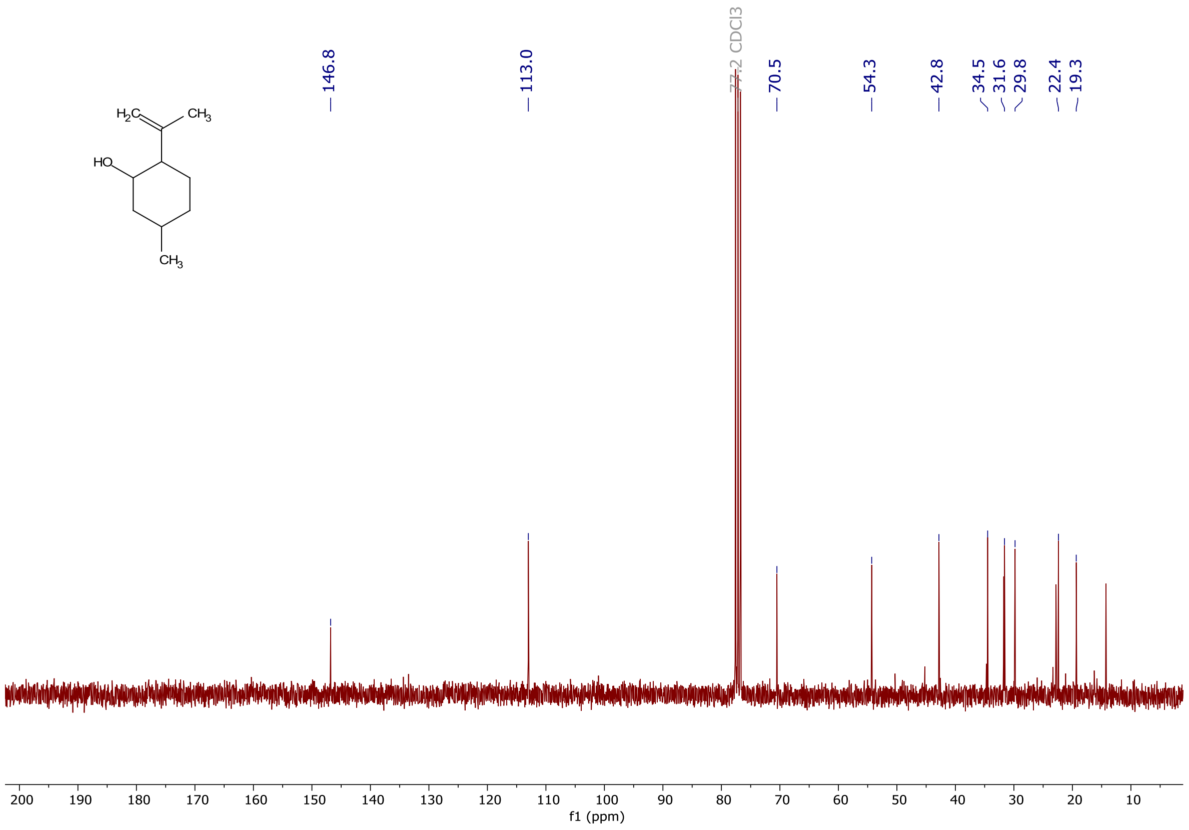

^{13}C NMR Spectrum of **3b** (101MHz, $CDCl_3$)

7. Optimized XYZ structures

Reactant, case a

Si	-0.18469092	2.15611141	-0.13118281
O	-0.24364957	0.50846668	-0.58610444
C	-0.35366883	-0.62573191	0.29175986
C	-1.84673703	-0.91032031	0.62633384
C	0.37940575	-1.79905243	-0.38057901
C	1.91285817	-1.64666171	-0.50409382
C	2.62880901	-1.76306116	0.85345890
C	2.47900967	-2.66781377	-1.50705565
C	-2.68238028	-1.31327731	-0.59953143
C	-2.00314933	-1.91365872	1.78161876
C	-1.74435685	2.66746734	0.76313017
C	1.31282835	2.48392029	0.97478572
C	-0.04694231	3.05099562	-1.78629120
H	0.14905365	-0.39442261	1.25907623
H	-2.23589773	0.06855047	0.98743411
H	-0.05140233	-1.92033379	-1.39612116
H	0.15227983	-2.73631612	0.17043867
H	2.10629373	-0.63046310	-0.91115832
H	3.72067482	-1.60260441	0.74589888
H	2.25646162	-1.02320307	1.58941917
H	2.48153651	-2.77280356	1.29165719
H	2.01862477	-2.55198038	-2.50926377
H	3.57616933	-2.55632133	-1.62400470
H	2.28437136	-3.70771890	-1.16936961
H	-3.76388055	-1.31400544	-0.35623248
H	-2.52544878	-0.60922975	-1.44025406
H	-2.42481158	-2.33149388	-0.95595212
H	-1.41723250	-1.60655040	2.67182213
H	-3.06509463	-1.99230462	2.08964581
H	-1.66960917	-2.93169460	1.49392505
H	-2.70995348	2.79838831	0.24286022
H	-1.79479692	2.83155981	1.85395172
H	2.25381241	2.18747254	0.46958495
H	1.37813057	3.56552473	1.21297413
H	1.24701993	1.93234569	1.93393026
H	0.01488505	4.14747006	-1.63485009
H	0.86085084	2.72860511	-2.33530950
H	-0.92927458	2.84263253	-2.42404310

TS 1,5-HAT, case a

Si	0.65019375	2.00915574	-0.12177917
O	0.50052449	0.34994266	-0.56525303
C	-0.47760371	-0.47296774	0.09808515

C	-1.90974261	0.02581193	-0.19828253
C	-0.23439401	-1.93606126	-0.31526108
C	1.13505522	-2.52711938	0.09131220
C	1.30608884	-2.64653783	1.61619619
C	1.35277599	-3.88983117	-0.59029925
C	-2.35355627	-0.04983898	-1.65156229
C	-2.95947192	-0.41590434	0.80852429
C	-1.10470260	2.59606400	0.15930478
C	1.70328541	2.14663755	1.44294499
C	1.50616485	2.81578229	-1.59636487
H	-0.33601718	-0.39257709	1.20195448
H	-1.69218034	1.30420443	0.01908862
H	-0.33571537	-1.99844952	-1.41877748
H	-1.04602602	-2.56329596	0.11237449
H	1.91259900	-1.82956805	-0.28853028
H	2.30242162	-3.05969914	1.87293673
H	1.21366217	-1.66854476	2.12858701
H	0.54141071	-3.32531234	2.04938261
H	1.27878546	-3.81030593	-1.69382806
H	2.35085616	-4.30737302	-0.34706943
H	0.59285266	-4.62751710	-0.25631757
H	-3.25913307	0.56626934	-1.82518763
H	-1.56189322	0.30513231	-2.34042767
H	-2.61228522	-1.09157105	-1.94371052
H	-2.63138181	-0.23596437	1.85225281
H	-3.91692292	0.12103255	0.65159053
H	-3.18228425	-1.50256393	0.71464264
H	-1.61634036	3.16398026	-0.63941717
H	-1.42486699	2.91129601	1.16911979
H	2.70821728	1.70556670	1.28424193
H	1.83816713	3.21037098	1.72766034
H	1.22884968	1.62514311	2.29834830
H	1.71204758	3.88533569	-1.38837467
H	2.47408959	2.31960026	-1.81309892
H	0.87637094	2.75899976	-2.50647070

TS 1,6-HAT, case a

Si	0.84585017	1.72674121	0.03464697
O	-0.48193929	0.82441458	-0.56364941
C	-0.98777615	-0.40367555	-0.01742476
C	-2.50810498	-0.49604613	-0.33149837
C	-0.18866300	-1.59628385	-0.59206500
C	1.21688206	-1.83296440	-0.02969963
C	1.25530603	-2.26912658	1.43021728
C	2.09076487	-2.68684795	-0.93968018
C	-3.17276799	-1.68951569	0.37979251

C	-3.24587943	0.80499670	0.02965805
C	2.36666280	0.64369940	-0.04356138
C	0.92603244	3.19940291	-1.14475745
C	0.50891627	2.28678027	1.81108015
H	-0.88498486	-0.38971574	1.09383795
H	-2.59966391	-0.64754480	-1.43106077
H	-0.13288853	-1.44388594	-1.69088988
H	-0.77069084	-2.53262209	-0.44256901
H	1.78389078	-0.65275266	-0.03425845
H	2.29648636	-2.32981861	1.80619178
H	0.69646698	-1.57814628	2.09245550
H	0.80128838	-3.27853238	1.55111110
H	2.14640143	-2.26970018	-1.96546812
H	3.12382990	-2.77722500	-0.54724711
H	1.68092467	-3.71861646	-1.02720271
H	-4.25455554	-1.72885154	0.14253738
H	-2.73736209	-2.66453208	0.08752310
H	-3.07896902	-1.59654097	1.48215076
H	-2.82981854	1.67489600	-0.51090679
H	-4.32229881	0.72596459	-0.22268170
H	-3.17469157	1.01322636	1.11806737
H	3.04699790	0.59585428	0.82639919
H	2.92057059	0.60117239	-1.00014412
H	-0.01994492	3.77820311	-1.13139982
H	1.74856346	3.88401932	-0.85431410
H	1.11306511	2.86742109	-2.18593658
H	1.33705735	2.93130665	2.17132900
H	-0.43088602	2.87109872	1.87814174
H	0.42835410	1.42672387	2.50561943
	Reactant, case b		
Si	2.00744083	1.09191367	-0.04348137
O	0.55887023	0.41906124	-0.64325780
C	-0.66030346	0.09264909	0.04761032
C	-1.82988366	0.90031357	-0.54549410
C	-0.93068781	-1.42106305	-0.07017201
C	0.09303987	-2.31788708	0.64199189
C	-0.14386741	-3.84725658	0.54371190
C	-1.77461602	2.43461715	-0.38991841
C	2.22480426	0.71410538	1.77483984
C	3.36197183	0.26966716	-1.07547499
C	2.05820104	2.96301045	-0.30722364
C	-1.45507034	-4.29536061	1.21582465
C	-0.04502099	-4.37590159	-0.89951226
C	-2.88282435	3.09845477	-1.22719875
C	-1.85806549	2.88515922	1.07944382

H	-0.54864227	0.34208850	1.12796558
H	-1.89090142	0.64776580	-1.62649859
H	-0.97988448	-1.66858810	-1.15201481
H	-1.94404381	-1.61216631	0.33881929
H	1.10602685	-2.09942743	0.24443754
H	0.68804015	-4.31452891	1.11626315
H	-0.79848107	2.77244052	-0.79755970
H	2.26605704	1.49582841	2.55321236
H	2.34359141	-0.31375571	2.15986473
H	3.17905189	0.43219786	-2.15767069
H	4.35589372	0.69636940	-0.83186141
H	3.40163285	-0.82283588	-0.89429347
H	3.06053967	3.35154209	-0.03117496
H	1.87547124	3.22076014	-1.36982552
H	1.30678023	3.49082140	0.31205703
H	0.12343889	-2.03750685	1.71753677
H	-2.34626379	-3.91581954	0.67504541
H	-1.53251518	-5.40139285	1.23712816
H	-1.51865840	-3.93446843	2.26264810
H	0.90958451	-4.07513332	-1.37752673
H	-0.09710571	-5.48337638	-0.92123373
H	-0.87156645	-3.99673476	-1.53480291
H	-2.77202645	0.52777420	-0.08756507
H	-3.88912648	2.78680642	-0.87573426
H	-2.83430889	4.20423265	-1.15875647
H	-2.80431445	2.82303127	-2.29847434
H	-1.80315065	3.98959266	1.16091596
H	-2.81669393	2.56364408	1.53888096
H	-1.03658437	2.46827300	1.69514408

TS 1,7-HAT, case b

Si	1.93459527	-0.45145692	-0.07991921
O	0.54129588	0.52173954	-0.30645258
C	-0.45893958	0.80986619	0.68232585
C	-1.40949256	1.88529258	0.12541012
C	-1.21512911	-0.45778490	1.14535917
C	-1.83516609	-1.31336442	0.01082697
C	-1.19206204	-2.68804315	-0.20187972
C	-0.76366567	3.20475725	-0.35274466
C	1.51360203	-2.27248541	-0.25890117
C	3.07659233	0.10436139	-1.48005109
C	2.70028087	-0.10133125	1.61652977
C	-1.38326045	-3.66221908	0.95459938
C	-1.50400306	-3.30080465	-1.56123404
C	-1.83055174	4.12979049	-0.96526683
C	0.01551868	3.92914196	0.75929945

H	0.04047092	1.22921291	1.58615332
H	-1.98352090	1.44929905	-0.72016782
H	-1.99415000	-0.13186028	1.86435811
H	-0.51347643	-1.07796657	1.74164548
H	-2.92083198	-1.47050773	0.20445567
H	0.08838549	-2.43985941	-0.22607512
H	-0.04454017	2.94305441	-1.15898022
H	1.80826194	-2.95234714	0.56265704
H	1.71712671	-2.73301963	-1.24462869
H	3.31473188	1.18419834	-1.39947331
H	4.02900345	-0.46312700	-1.45720578
H	2.60289309	-0.07042598	-2.46735633
H	3.66733714	-0.63661808	1.71120517
H	2.89477312	0.98140974	1.75537303
H	2.04711885	-0.44318199	2.44425396
H	-1.78881374	-0.75524194	-0.94665714
H	-2.45415526	-3.95415431	1.04480223
H	-0.80013706	-4.59326158	0.80444843
H	-1.08051824	-3.22686864	1.92774989
H	-1.23552507	-2.61644726	-2.39141105
H	-0.96403107	-4.25715207	-1.71460750
H	-2.59174562	-3.51981736	-1.65570571
H	-2.15478535	2.11413473	0.91834374
H	-2.58036684	4.43311297	-0.20441518
H	-1.37598552	5.05581185	-1.37220292
H	-2.37700822	3.63007987	-1.79086377
H	-0.64932627	4.18432418	1.61149339
H	0.84771941	3.31468333	1.15553898
H	0.45430025	4.87614424	0.38474838

TS 1,6-HAT, case b

Si	1.91389753	1.24708215	0.09397963
O	0.74776842	0.13061869	-0.47892404
C	-0.56889879	-0.07044198	0.06243181
C	-1.56709664	0.95666102	-0.51901396
C	-1.00240735	-1.51534679	-0.24901905
C	-0.11200295	-2.58733085	0.39889739
C	-0.47458504	-4.06413981	0.09954498
C	-1.48169629	2.39237652	0.00887526
C	1.18948358	2.96470729	-0.05000959
C	2.37364442	0.85747433	1.88770629
C	3.37933831	0.97203270	-1.06543046
C	-1.86261430	-4.46062749	0.63600323
C	-0.32407345	-4.42636601	-1.38970948
C	-2.13895516	3.41290044	-0.91141359
C	-1.87462633	2.55634646	1.47253150

H	-0.53639285	0.04377207	1.17148328
H	-1.43778622	0.95299873	-1.62226695
H	-1.01562406	-1.63403490	-1.35364397
H	-2.05129077	-1.63443281	0.09126593
H	0.93783666	-2.41205916	0.08449087
H	0.27222133	-4.67306184	0.65604686
H	-0.20593093	2.68478888	-0.02144417
H	1.26919633	3.47344781	-1.02923820
H	1.30120561	3.67443357	0.79019423
H	2.73183557	-0.18627045	1.99400232
H	3.18537490	1.53409486	2.22588146
H	1.51354522	0.99812190	2.57246855
H	4.21744519	1.64607893	-0.79552050
H	3.74985050	-0.07186289	-1.00996511
H	3.09982770	1.18366671	-2.11731968
H	-0.12630897	-2.43991177	1.50135114
H	-2.67788799	-3.93311985	0.09935890
H	-2.03992293	-5.54847979	0.51380432
H	-1.96297396	-4.22420151	1.71502230
H	0.68319807	-4.16200239	-1.77147287
H	-0.46892158	-5.51403875	-1.55003190
H	-1.06982722	-3.89962639	-2.01991089
H	-2.59953771	0.58194960	-0.32891809
H	-3.23839113	3.24451354	-0.96822073
H	-1.98557738	4.44927997	-0.54813288
H	-1.74466632	3.34740104	-1.94565687
H	-1.69426131	3.59157628	1.82593465
H	-2.95816991	2.34288854	1.61424109
H	-1.31523523	1.87029388	2.13939139

Reactant, case c

Si	2.02386003	-1.31095333	-0.13379903
O	0.44449434	-1.08826984	-0.73862085
C	-0.78757931	-0.90555573	-0.01955698
C	-1.37430090	0.48216361	-0.34179931
C	-1.78379455	-2.01969916	-0.39054470
C	-1.39407717	-3.46493190	-0.01396727
C	-1.27079643	-3.66985460	1.50632004
C	-2.39531699	-4.46472769	-0.62052734
C	-0.51563666	1.67629112	0.09592427
C	2.15628728	-0.75069764	1.64408568
C	2.53653433	-3.12520089	-0.27217596
C	3.12316076	-0.26445888	-1.26501773
C	-1.15831222	3.03924768	-0.20771215
C	-0.30304917	4.24174603	0.22099358
C	-0.95153420	5.60537129	-0.06716950

C	-0.09063369	6.80163852	0.35893776
H	-0.58224497	-0.94715484	1.07499867
H	-1.55982110	0.53371450	-1.43735369
H	-1.95172397	-1.96457354	-1.48817071
H	-2.75975010	-1.77782460	0.08428427
H	-0.40091351	-3.66960703	-0.46719835
H	-0.98814390	-4.71501305	1.74538349
H	-0.50348509	-3.00988188	1.95733185
H	-2.23526471	-3.45916826	2.01510907
H	-2.45943292	-4.35961735	-1.72263435
H	-2.10630330	-5.51172983	-0.39726870
H	-3.41514100	-4.30572230	-0.21061002
H	0.47529203	1.61993531	-0.40132554
H	-0.31448025	1.60262420	1.18729825
H	1.90067475	0.27177407	1.97220083
H	2.53592583	-1.40245648	2.45025940
H	2.39760584	-3.49702356	-1.30750635
H	3.61076158	-3.23282239	-0.01560333
H	1.95532927	-3.77600314	0.41010704
H	4.19505930	-0.42076671	-1.02883449
H	2.96515844	-0.54926600	-2.32584981
H	2.90100673	0.81615752	-1.16214527
H	-2.37063656	0.55171929	0.14476040
H	-2.14950826	3.09737658	0.29458428
H	-1.37104158	3.11016904	-1.29755510
H	0.68424114	4.19001484	-0.28971614
H	-0.08145882	4.16521767	1.30886888
H	-1.93648601	5.65547021	0.44665184
H	-1.17727951	5.67886164	-1.15354927
H	-0.59078751	7.76538903	0.13551584
H	0.12019000	6.78061331	1.44818407
H	0.88689967	6.80359564	-0.16614878

TS 1,6-HAT (2º), case c

Si	1.98196342	-0.84487027	-0.13784405
O	0.45919371	-1.46907851	-0.61279270
C	-0.82004489	-1.17153091	-0.02983174
C	-1.34376423	0.21846997	-0.45902652
C	-1.80023180	-2.28058769	-0.45245979
C	-1.41117208	-3.72292636	-0.06465514
C	-1.37225976	-3.93789346	1.45896501
C	-2.35919356	-4.73358017	-0.73476418
C	-0.67040028	1.41808013	0.18771583
C	1.95330938	1.00665130	-0.39401964
C	2.33090869	-1.25305851	1.67681121
C	3.19061989	-1.73994498	-1.28086684

C	-1.09212020	2.78823047	-0.31521494
C	-0.39178498	3.96769330	0.37894222
C	-0.84217205	5.34596852	-0.13037215
C	-0.13168859	6.51853649	0.55799774
H	-0.72985845	-1.17486213	1.08179759
H	-1.27648417	0.28819351	-1.56700118
H	-1.91498096	-2.22268454	-1.55684500
H	-2.79756514	-2.04434729	-0.02224706
H	-0.38929830	-3.90185004	-0.46357492
H	-1.08145329	-4.97874651	1.70700672
H	-0.64896678	-3.26479405	1.96012810
H	-2.36951486	-3.75297161	1.91133207
H	-2.35963788	-4.62265139	-1.83813801
H	-2.06799056	-5.77746630	-0.50011853
H	-3.40377929	-4.59246219	-0.38521695
H	0.63594852	1.28104192	-0.07468017
H	-0.66392429	1.35137041	1.29625125
H	2.01419959	1.37367179	-1.43646788
H	2.52973167	1.64807011	0.29816614
H	2.28544453	-2.34570856	1.85868833
H	3.34658311	-0.90372505	1.95518203
H	1.60860873	-0.75906649	2.35720686
H	4.22988468	-1.41206035	-1.07588588
H	3.14582546	-2.83901248	-1.13812344
H	2.97023853	-1.52092356	-2.34515301
H	-2.43195426	0.25640200	-0.22050602
H	-2.19635691	2.89535238	-0.18580927
H	-0.92492893	2.84699776	-1.41407674
H	0.70781410	3.87055172	0.24777243
H	-0.56987374	3.90468408	1.47517502
H	-1.94123974	5.44419757	0.00699889
H	-0.67253224	5.40187500	-1.22784266
H	-0.48155363	7.49509096	0.16706835
H	-0.31081490	6.51395952	1.65303351
H	0.96632466	6.47207994	0.40473613

TS 1,6-HAT (3º), case c

Si	1.87143187	-1.74954032	0.05600317
O	0.63324545	-0.73732295	-0.55687093
C	-0.70282188	-0.61747900	-0.04088105
C	-1.24207545	0.77245095	-0.42372564
C	-1.61290842	-1.74150687	-0.58752958
C	-1.42800154	-3.14449763	-0.00081454
C	-1.81914929	-3.27860785	1.46631366
C	-2.00213225	-4.24633584	-0.88233942
C	-0.44525292	1.95433241	0.14622389

C	1.27760588	-3.51969284	-0.03437784
C	3.32382014	-1.40288970	-1.10021487
C	2.28539758	-1.26993551	1.83953961
C	-1.00144685	3.32574952	-0.26812393
C	-0.19801944	4.51661065	0.27711472
C	-0.75482079	5.88807840	-0.13773006
C	0.05693795	7.07360398	0.40017215
H	-0.67843578	-0.67895868	1.07249204
H	-1.27066796	0.83886556	-1.53388355
H	-1.46955635	-1.77275851	-1.68862802
H	-2.67284978	-1.43786345	-0.42391801
H	-0.13476632	-3.34333763	-0.01565775
H	-1.56848423	-4.28385721	1.86094495
H	-1.31544370	-2.52888669	2.10856139
H	-2.91619497	-3.13768927	1.59418397
H	-1.60825300	-4.19151440	-1.91736518
H	-1.77327300	-5.25333366	-0.47835696
H	-3.11076707	-4.16344905	-0.94835920
H	0.61284832	1.87281186	-0.17865279
H	-0.42835796	1.88393866	1.25649837
H	1.43898345	-4.19044090	0.82956077
H	1.39808200	-4.05333040	-0.99599350
H	3.60855086	-0.33117067	-1.08074927
H	4.20929205	-1.99773288	-0.79734507
H	3.07452355	-1.67458329	-2.14584323
H	3.15102158	-1.86262931	2.20077070
H	2.55051778	-0.19604878	1.91476864
H	1.43678537	-1.46434478	2.52554958
H	-2.29644708	0.83917348	-0.08171399
H	-2.05875589	3.41136135	0.06757222
H	-1.03549154	3.38554766	-1.37865392
H	0.85810821	4.43168641	-0.06281324
H	-0.16000586	4.45752589	1.38771300
H	-1.80857905	5.97264220	0.20711169
H	-0.79909555	5.94202464	-1.24757390
H	-0.37492834	8.04341629	0.08089680
H	0.08892606	7.07225434	1.50936259
H	1.10628114	7.04120868	0.04092691
